# Genetic dissection of plant architecture and yield-related traits in *Brassica napus*

**DOI:** 10.1038/srep21625

**Published:** 2016-02-16

**Authors:** Guangqin Cai, Qingyong Yang, Hao Chen, Qian Yang, Chunyu Zhang, Chuchuan Fan, Yongming Zhou

**Affiliations:** 1 National Key Laboratory of Crop Genetic Improvement, Huazhong Agricultural University, Wuhan 430070, China; 2 Key Laboratory of Rapeseed Genetics and Breeding of Agriculture Ministry of China, Huazhong Agricultural University, Wuhan 430070, China

## Abstract

An optimized plant architecture (PA) is fundamental for high-yield breeding but the genetic control of the important trait is largely unknown in rapeseed. Here plant architecture factors (PAFs) were proposed to consist of main inflorescence length proportion (MILP), branch height proportion (BHP), and branch segment proportion (BSP). Comparison of different genotypes in a DH population grown in diverse environments showed that an optimized PAF performance with MILP and BHP between 0.3–0.4 was important for high yield potential. In total, 163 unique quantitative trait loci (QTLs) for PA- and plant yield (PY)-related traits were mapped onto a high-density genetic map. Furthermore, 190 PA-related candidate genes for 91 unique PA QTLs and 2350 PY epistatic interaction loci-pairs were identified, which explain 2.8–51.8% and 5.2–23.6% of phenotypic variation, respectively. Three gene categories, transcription factor, auxin/IAA, and gibberellin, comprise the largest proportions of candidate genes for PA-related QTLs. The effectiveness of QTL candidate genes prediction was demonstrated by cloning of three candidate genes, *Bna.A02.CLV2*, *Bna.A09.SLY2*, and *Bna.C07.AHK4*. The study thus outlines a gene network for control of PA-related traits and provides novel information for understanding the establishment of ideal PA and for developing effective breeding strategies for yield improvement in rapeseed and other crops.

Plant architecture (PA) refers to the morphological characteristics and spatial arrangement of the plant corpus and is perhaps the most visible change that occurs during crop domestication. PA modifications have shown significant impacts on crop adaptation and yield potential^1^,^2^. A well-known example is the introduction of dwarf genes into wheat and rice, which resulted in reduced plant height and modified PA and, thus, greatly enhanced seed yield^3^,^4^. PA is also one of the most important and direct characteristics to distinguish various species. Therefore, a comprehensive understanding of the genetic control of PA in crops can not only provide insights into crop domestication history, morphogenesis and development, but also lay a foundation for designing efficient breeding strategies to improve crop yield^5^,^6^,^7^.

An optimal PA can improve leaf area index, photosynthetic efficiency, fertilizer tolerability, and harvest index, thus leading to increased seed yield. Therefore, breeding for an optimized PA has drawn great attention in major crops. For instance, improvements of maize PA, especially leaf angle, have significantly contributed to increase yield^8^,^9^,^10^. An ideal plant architecture (IPA) in rice has been proposed to reduce unproductive tillers, and to yield thicker and more sturdy stems and more grains per panicle than current rice varieties^6^. Various parameters used to quantitatively describe the suitable PA or IPA in different crops, but some common characteristics are often considered in breeding practices, such as (semi-)dwarfed and compact plant, rigid stem, and up-right and dark green leaves^2^. The majority of PA-related traits are quantitative traits that are affected by environmental factors and growth conditions, such as light, temperature, nutrition, and plant density. The genetic control of PA is complicated as the final establishment of a PA in a given species involves a series of developmental processes, such as the initiation and development of shoot apical meristem (SAM) and axillary meristem (AM; tillers, branches), stem and internode elongation, and the differentiation of floral organs in inflorescences^1^,^2^.

Over the last two decades, a number of genes involved in PA formation have been identified through reverse genetics and map-based cloning, which can be categorized into a wide array of biological pathways. Strikingly, many of these genes are involved in hormone biosynthesis and regulation. Genes controlling plant height are related to gibberellin (GA) metabolism and signal transduction^4^,^11^. Several genes that work as a master switch of SAM initiation and development, are related to auxin, indole acetic acid (Aux/IAA) biosynthesis and signal transduction^12^,^13^,^14^. More recently, genes for cytokinin (CK) metabolic regulation and transport, brassinosteroid (BR) biosynthesis and signal transduction, and strigolactone (SL) metabolism regulation and signal transduction have been demonstrated to be important in various PA-related traits, such as *CKX*^15^, *KN1*^16^, *BKI1*^17^, *BRI1*^18^, *D14*^19^, *D27*^20^. Moreover, several transcription factors (TFs) and cell cycle (CC) genes affect PA establishment and maintenance^5^,^21^,^22^,^23^. Although a large number of genes related to PA regulation and development have been identified in different species, our understanding of crop morphogenesis and developmental regulation is still limited. The lack of mutants linked to all aspects of plant morphogenesis is a bottleneck that must be relieved to fully define the network^2^,^24^.

Rapeseed (*Brassica napus*, AACC, 2n = 4x = 38) is one of the three major oil crops in the world, and it provides edible oil and raw materials for bio-energy applications. *B. napus* was formed approximately 7,500 years ago through the natural hybridization of its two progenitor diploids, *B. rapa* (AA, 2n = 2x = 20) and *B. oleracea* (CC, 2n = 2x = 18)^25^,^26^,^27^. These three species and Arabidopsis are thought to share a common ancestor^25^,^27^,^28^. *B. napus*, a relatively young species in terms of its evolutionary age, has a short history of artificial domestication (Approximately 400–500 years)^27^,^29^,^30^. To date, few studies on rapeseed PA have been reported, which described the composition^31^, properties of PA characters^32^, and genetic model and combining abilities of some PA-related traits^33^,^34^. More recently, two genes related to plant height, *dwf2* in *B. rapa*^35^ and *Bnrga-ds* in *B. napus*^36^, were identified as *AtRGA* homologs. However, the genetic control of PA in this important crop is largely unknown.

The current study was aimed to systematically dissect the genetic architecture of PA in rapeseed using a doubled haploid (DH) population, which was derived from two parents with different PA structures genotyped using *B. napus* 6 K SNP (single nucleotide polymorphism) arrays. The parameters called plant architecture factors (PAFs) in rapeseed were introduced to dissect complex PA traits into simple components that are more stable across environments and genotypes. Quantitative trait loci (QTLs) and candidate genes involved in the genetic control of PA and their interactions with plant yield (PY) were identified. Our results provide novel information about the genetic architecture of PA in *B. napus*, which is important for improving yield by manipulating PA-related traits.

## Results

### Quantitation of plant architecture with plant architecture factors derived from primary morphological traits

**Figure 1:**
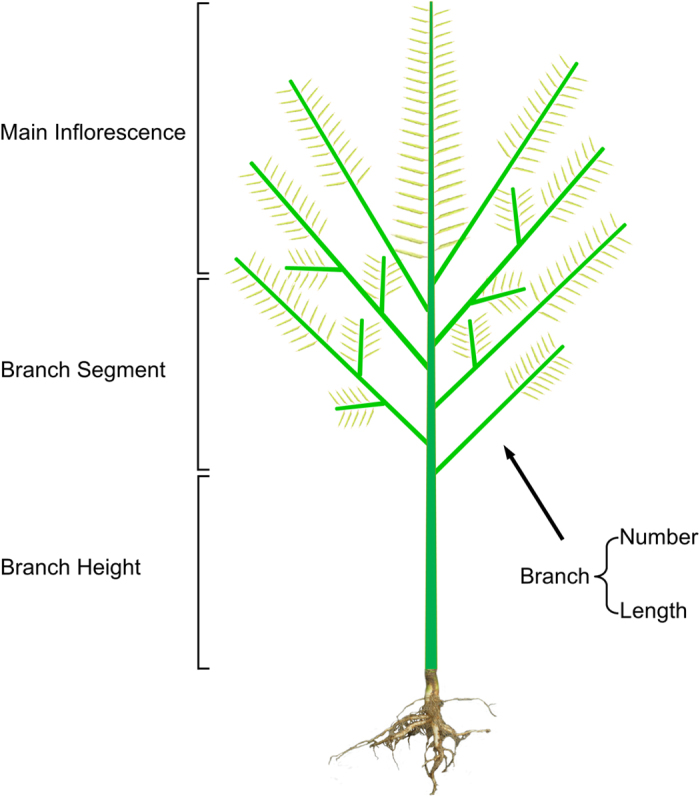
Schematic diagram of rapeseed plant architecture (PA).

To quantify variations in PA-traits, we divided a plant into three segments (Fig. 1): main inflorescence length (MIL), branch height (BH), and branch segment (BS). These three segment measurements were simply divided by plant height (PH) so as to obtain three proportion values: MIL proportion (MILP), BH proportion (BHP) and BS proportion (BSP). These three proportion parameters were termed plant architecture factors (PAFs) and used them to describe the PA components in the following analyses (Fig. 1). Similarly, a series of secondary parameters were obtained based on the measurements of primary morphological traits, including branch average length (BAL = BTL/BN), total number of plant silique (PSN = MISN + BSN), PY (MIY + BY), MISN proportion (MISN/PSN), and MIY proportion (MIY/PY). Together, there are 12 primary traits (from direct measurement) and 8 secondary traits (calculated from primary traits), which can be grouped into 10 PA-related traits and 10 PY-related traits.

The MIL, MILP, BN, BAL, SSN, BY, MIY, PY, and TSW between the two parents (Hua_5 and J7005) were significantly different in all environments examined (Supplementary Table S1). Furthermore, majority of 20 traits showed continuous distributions in the DH population in four environments (Supplementary Table S1, Figs S1–S4). All of the traits were significantly different in terms of genotype (G), environment (E), and genotype by environment (G × E) based on ANOVA results (Supplementary Table S2). The broad-sense heritabilities (*h*^*2*^) of all traits varied from 50–92.8%, with higher percentages associated with PA-related traits compared to PY-related traits (Supplementary Table S2). The correlations between PY- and PA-related traits based on different environments were highly significant (Supplementary Table S3).

### Optimized plant architecture factors are important components for high yield potential

To evaluate the effects of PAFs on PY and PY-related traits, the DH lines with significant differences in PY were grouped into two pools (i.e., high-yield and low-yield pools) in each environment (Table 1). The average performances of the other traits were compared between these two pools. The high-yield pools had similar PY levels as Hua_5, a high-yielding control cultivar grown under the same conditions. However, the yield components, i.e., PSN, SSN and TSW varied significantly. The high-yield pools in all or most of the environments had higher PSN and SSN but similar TSW compared to those in the low-yield pools, while Hua_5 exhibited a similar yield level to the high-yield pool but with more SSN and heavier TSW (Table 1). The results suggested that high-yield genotypes may have different combinations of yield components, which is consistent with previous studies^37^,^38^. However, the MILPs of the high-yield pools and Hua_5 were stable, with a range of 0.35–0.4 in all winter-type rapeseed environments, while the MILPs of the low-yield pools were higher than 0.4 (Table 1). Similarly, the BHPs of high-yield pools were between 0.32–0.38 across environments, while the BHPs of the low-yield pools were higher than 0.4 or lower than 0.3 (Table 1).

The population and control in spring-growing environment (GS11) performed differently due to dramatic differences in ecological and farming conditions and had a relatively low yield level compared to the same genotypes in winter-type rapeseed environments, suggesting that the DH population and Hua_5 are not optimized to be spring-growth ecotypes. Accordingly, the PA parameters did not distribute into the optimal norm as they did in winter-growing conditions, although we observed similar trends in the high- and low-yield pools (Table 1).

Together, these results indicate that the MILP and BHP in different high-yield genotypes should be at an appropriate range to reach a certain yield level. Compared the three PY components (PSN, SSN, and TSW), PAFs may function as a stable and easy-to-use measurement in selecting high-yield genotypes.

To verify the above results, we further analyzed the performance of three high-yield cultivars used as check (CK) varieties in the national rapeseed regional trial in China during 2006–2010, which was conducted for winter-type rapeseed across major growth regions^37^. All cultivars were high-yield and widely grown in large scales in the corresponding ecological areas (Table 2). The PY components varied greatly from year to year in terms of growth density, but the PAFs were much more consistent (Table 2). For example, PSN and BN ranged from 222.7 to 483.5 and 5.9 to 9.6, respectively, while all MILPs were between 0.3–0.4 in different regions, years and growth densities (Table 2). The results further indicated that optimized PAF performance was important to achieve high yield potential.

### PA-related QTLs are more stable than PY-related QTLs

**Figure 2:**
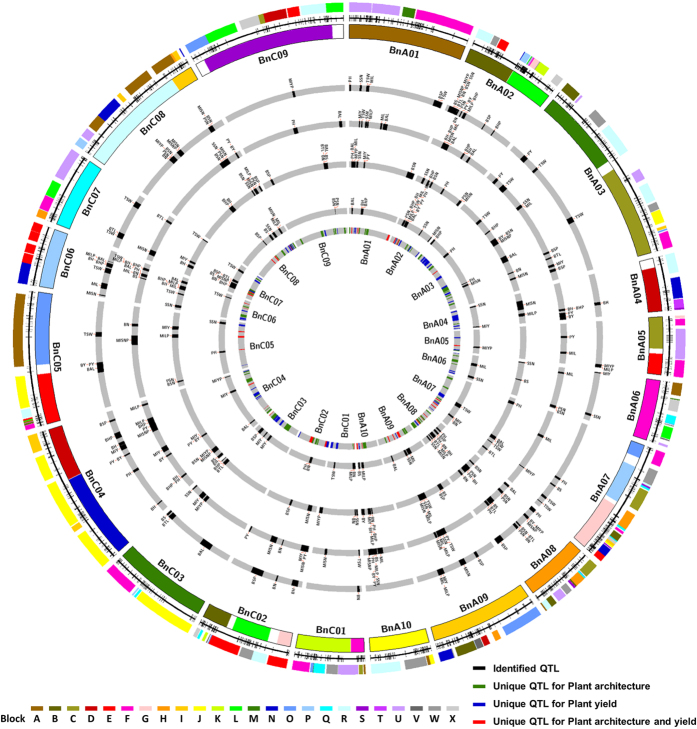
Mapping quantitative trait loci (QTLs) for PA- (plant architecture) and yield- related traits onto the *Brassica napus* genome. The colored blocks in the outermost circle represent the conserved Arabidopsis blocks aligned with the *B. napus* genome identified through single-nucleotide polymorphism/simple sequence repeat (SNP/SSR) markers in the *B. napus* genetic linkage groups, which are represented by the second outer circle. The third circle represents the *B. napus* genome reconstructed with fragments of homoeologous exchanges defined by Chalhoub *et al.*^27^. The fourth to seventh circles represent QTLs identified in WH09, WH10, HG10, and GS11 environments, respectively. The black blocks represent the confidence intervals of the identified QTLs. The inner (eighth) circle represents unique QTLs. The green, blue, and red blocks represent confidence intervals of unique QTLs of PA, PY, or both.

A total of 346 QTLs for 20 examined traits were identified in four environments. These QTLs were evenly mapped in the A and C rapeseed genomes (Table 3, Fig. 2, and Supplementary Table S4) and could explain 2.8–51.8% (10.3% per QTL on average) of the phenotypic variation (*R*^*2*^), with an average confidence interval of 4.19 cM. Among 167 PY-related trait QTLs identified, 99 (59.3%) exhibited additive effects from Hua_5, and 68 (40.7%) exhibited additive effects from J7005 (Table 4). Among the 179 PA-related trait QTLs identified, 73 (40.8%) exhibited additive effects from Hua_5, and 106 (59.2%) exhibited additive effects from J7005 (Table 4). Such distributions were consistent with the phenotypic performances of the two parental lines, i.e., Hua_5 being a high-yield cultivar and J7005 being a cultivar with special PA component traits (Fig. 1 and Supplementary Table S1).

Compared with PY-related trait QTLs, the log likelihood of the odds (LOD) scores of the PA-related trait QTLs (LOD = 7.45, *R*^*2*^ = 10.3% on average) were higher than those of PY-related traits (LOD = 6.48, *R*^*2*^ = 10.3% on average). Although the majority of the above QTLs showed minor effects on phenotypic variations with *R*^*2*^ < 10% (Supplementary Table S4), 47 QTLs had *R*^*2*^ > 20% in one environment (26 QTLs) or > 10% in any two environments (21 identified QTLs). We referred to such QTLs as major QTLs, and they accounted for 13.6% of all identified QTLs. There were 24 PA-related trait major QTLs (LOD = 19.39, *R*^*2*^ = 27.4% on average) and 23 PY-related trait major QTLs (LOD = 11.57, *R*^*2*^ = 20.1% on average). We identified a major QTL for the PA-related trait MIL, with an *R*^*2*^ of up to 51.8% (LOD = 27.4) (Supplementary Table S4). We also repeatedly identified 41 QTLs in two or more environments, 22 of which were PA-related trait QTLs with an average LOD = 7.98 and *R*^*2*^ = 10.0%, and 19 of which were PY-related trait QTLs with an average LOD = 6.83 and *R*^*2*^ = 10.5%. The above results indicated that PA-related trait QTLs may be more stable than PY-related trait QTLs.

The PAFs (BHP, MILP, and BSP) are secondary traits derived from the four primary traits PH, BH, MIL, and BS. Notably, there were 42.9% (9/21, BHP), 66.7% (14/21, MILP), and 86.7% (13/15, BSP) QTLs for secondary traits belonging to new QTLs not identified by the four primary traits (Supplementary Table S4). Moreover, 2 of the newly identified QTLs were major QTLs (*R*^*2*^ > 20%), and the *R*^*2*^ values of 9 QTLs were > 10%. These results suggested that PAFs as PA measurements may be less affected by environment, and more suitable for the identification of PA-related QTLs.

### Unique PA-related QTLs have pleiotropic effects on yield and yield components

The above identified QTLs were integrated into unique QTLs with a two-round strategy by QTL meta-analysis. First, 301 consensus QTLs (same traits in different environments) were generated by merging 346 identified QTLs. Second, the overlapping consensus QTLs of different traits were pooled into 163 unique QTLs (Table 3 and Supplementary S5). By doing so, the average confidence interval dropped to 3.19 cM. Among the unique QTLs, 69 (Fig. 2, blue sites on innermost circle; Supplementary Table S5), 58 (red sites on innermost circle) and 36 (green sites on innermost circle) were related to PA-traits, PY-traits, or both, respectively. Moreover, 94 (57.7%) unique QTLs were responsible for a single trait, with 51 related to PA traits and 43 related to PY traits. The remaining 69 unique QTLs (42.3%) were pleiotropic, affecting two or more traits, with 18 (26.1%) and 15 (21.7%) related to PA- and PY-traits, respectively, and 36 (52.2%) related to both (Table S5). These 36 pleiotropic QTLs simultaneously controlled PA- and PY-related traits, which corresponded to 145 identified QTLs with 68 PA-related QTLs (LOD = 7.82, *R*^*2*^ = 10.6% on average) and 77 PY-related QTLs (LOD = 6.54, *R*^*2*^ = 10.3% on average). The results clearly indicate that PA can affect PY-related traits to a large extent. Moreover, the effect of PA-related traits on PY was similar to the effect of PY components (PSN, SSN, and PY) on PY.

To estimate the influence and effect of the PAFs on PY, pleiotropic QTLs of PAFs, PY, and PY components traits were selected to analyze the relationship and effects between PAFs and PY. Four pleiotropic QTLs simultaneously controlled BHP and MILP traits (Supplementary Table S5), two of which were major QTLs and were repeatedly identified as QTLs for both BHP and MILP. Interestingly, the additive (A) effects of these four BHP QTLs were opposite of those of MILP. Meanwhile, three pleiotropic QTLs simultaneously controlled PAFs and PY, one of which was a major QTL that controlled PY (*R*^*2*^ = 21.9% in WH09, and *R*^*2*^ = 14.6% in GS11). Similarly, 8 pleiotropic QTLs controlled both PAFs and PY components. These results clearly suggest that plants with optimized PAFs have better yield potential at the QTL level.

### Identification of candidate genes for PA-related QTLs

To identify candidate genes for unique QTLs, we collected 256 genes that are involved in PA development and regulation in Arabidopsis and other species (Table 5 and Supplementary S6). These genes can arbitrarily be categorized into 8 groups based on their assigned functions and metabolic pathways, namely, Auxin/IAA- (40), CK- (22), GA- (42), BR- (25), and SL- (22) regulated pathways, TFs (37), CC genes (26), and other types of genes (42) (Table 5 and Supplementary Table S6). A total of 1080 homologous genes were identified in the *B. napus* genome. However, there were 40 plant architecture genes that could not be identified in *B. napus* genome (Supplementary Table S6).

**Figure 3:**
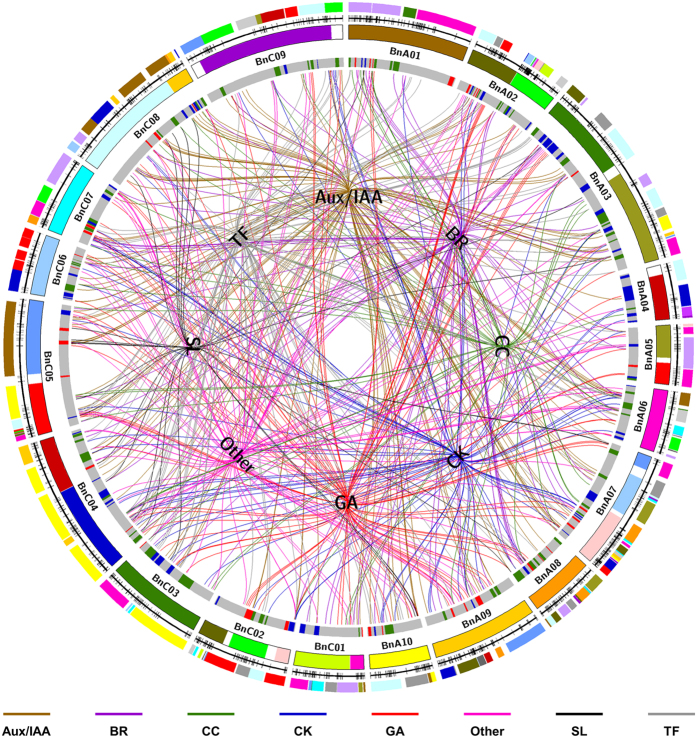
Schematic of homologous plant architecture (PA) genes in *Brassica napus*. The colored blocks in the outermost circle represent the conserved Arabidopsis blocks in the *B. napus* genome identified through single-nucleotide polymorphism/simple sequence repeat (SNP/SSR) markers in the *B. napus* genetic linkage groups, which are represented by the second outer circle. The third circle represents the *B. napus* genome reconstructed with fragments of the homoeologous exchanges as defined by Chalhoub *et al.*^27^. The fourth circle represents the unique QTLs (quantitative trait loci). The inner circle represents 8 categories of PA genes [auxin/indole acetic acid (Aux/IAA), gibberellin (GA), cytokinin (CK), brassinosteroid (BR), strigolactone (SL), transcription factor (TF), cell cycle gene (CC), and other types of genes (Other)]. The lines connect the positions of the category name (in the inner circle) and the positions of genes in the *B. napus* genetic map (in the fourth circle) of the same category. The different categories are expressed with different color lines.

These homologous genes were mapped onto the *B. napus* genetic linkage map according to the relationship between the *B. napus* genetic map and the physical map^39^. After the exclusion of homologous genes located on scaffolds and unplaced sequences in the reference genome, 826 homologous genes (76.5%) were anchored into the *B. napus* genetic map (Table 5, Fig. 3, and Supplementary Table S8). These genes were evenly distributed across all *B. napus* linkage groups (Table 5, Fig. 3, and Supplementary Table S8).

Next, we predicted the candidate genes of 105 unique QTLs, except for 58 PY-related QTLs (69 unique QTLs only related to PA; 36 unique QTLs were pleiotropic QTLs related to both PA and PY). In total, 190 homologous genes were located in or near the confidence intervals of 91 unique QTLs (Fig. 3 and Supplementary Table S9), which were considered as candidate genes. Among the 91 unique QTLs with candidate genes, 61 were related to PA only, and the remaining 30 were pleiotropic QTLs. We did not identify any homologous genes near the remaining 14 unique QTLs, and considered these loci unknown or PA-specific loci in *B. napus* (Supplementary Table S9).

To further verify the effectiveness and accuracy of this method in the identification of QTL candidate genes, three QTLs were chosen according to the importance and effectiveness to clone the candidate genes. DNA fragments containing three QTLs candidate genes were cloned in two parents (Hua_5 and J7005; Supplementary Figs S5–S7): *CLV2* for *quPAY.A02_24.39* (ID_15 in Table S9), *SLY2* for *quBSP.A09_26.7* (ID_77 in Table S9), and *AHK4* for *quPAY.C07_21.9* (ID_139 in Table S9). The cloned DNA sequences of the candidate genes were aligned against the *B. napus* reference genomic and coding sequences (CDS. Darmor-*bzh*^27^), as well as the sequences of their respective Arabidopsis homologous genes (Supplementary Figs S5–S7). There were numbers of SNPs in the two parents in all three cloned genes at genomic DNA levels (Supplementary Figs S5–S7). At amino acid (aa) level, Bna.A02.CLV2 in J7005 and Bna.C07.AHK4 in Hua_5 harbored a precocious termination at the 382 aa residues and 383 aa residues, respectively (Supplementary Figs S5,S7), while four aa mutations for Bna.A09.SLY2 were identified between the two parents (Supplementary Fig. S6). The above results indicated that the candidate gene sequences between two parents had obvious differences, providing further evidence that the method for predication of the QTL candidate genes is effective and veracious.

Because of the high homoeology between the A and C genomes of *B. napus*, some QTLs identified in A genome of *B. napus* also had corresponding orthologous QTLs in C genome (vice versa), and these QTLs also identified same candidate genes. For instances, a QTL for SSN was identified in A03 (identified QTL ID_103; unique QTL ID_30), and a corresponding orthologous QTL for SSN was also identified in the corresponding orthologous region of C03 (identified QTL ID_151; unique QTL ID_108). These two QTLs identified the same candidate genes *PsLP* and *LeSP* (Table S9). Similarly, two QTLs for AHP in A04 and C04 (identified QTL ID_110, 305; unique QTL ID_42, 68) also had two corresponding orthologous QTLs in C04 and C07 (identified QTL ID_60, 59, 332; unique QTL ID_118, 137). However, some of QTLs did not identify corresponding orthologous counterparts in the two subgenomes. For instance, *qPH.A01* (ID_1), *qMILP.A02* (ID_17), *qBS.C04* (ID_241), *qTSW.C05* (ID_65) were only identified in A or C genome of *B. napus*. Above results indicated that the A- and C-subgenomes of *B. napus* may experience the selections at both same and different direction during the process of the parallel evolution.

To understand how pleiotropic QTLs may influence PY through PA, we examined 3 pleiotropic major QTLs for PY (Supplementary Table S10). There were 8 candidate genes for the 3 pleiotropic QTLs, which have been previously identified to be involved in regulating PA or PA-related traits. For instance, *AtCUC2* and *PhNAM* influence embryo, flora, and SAM development. High *CUC2* transcript levels have been reported to increase floral organ number and to shorten internodes^40^,^41^,^42^; The *sp* tomato mutant disrupted the regularity of alternating vegetative and reproductive phases, and resulted in the characteristic scorpioid architecture of tomato inflorescence^43^; The *PsLF* genes determines the flowering node in pea and also affects inflorescence structure^44^. The above results indicate that the effects of loci on PY may largely be through the regulation of PA-related traits; therefore they exhibit pleiotropy on both PA- and PY-related traits.

### Epistatic loci pairs for PA- and PY-related traits are equally important to plant yield

To explore the influence of PA on PY, we analyzed all possible two-locus interactions between PA components and PY in the whole genome using a two-way ANOVA procedure^45^. Because only two genotypes (A and B) exist in each locus of a DH population, we could only identify the interactions of additive effects of two different loci (AA). In total, 2350 interaction loci-pairs were identified in the four environments, with 690 for WH09, 746 for WH10, 311 for HG10, and 603 for GS11 (Supplementary Table S11). These interactions can explain 5.18–23.58% PY variation variances (Var; 8.65% on average). Furthermore, PY was affected not only by relevant QTLs but also by a large number of epistatic interaction loci-pairs. We identified 92% (2167) of the total interaction loci-pairs (2350) in a specific environment, and 183 interaction loci-pairs existed in two or more environments, with only 3 interaction loci-pairs detected in three environments. The results indicate that the interaction loci-pairs with PY were highly influenced by environment.

**Figure 4:**
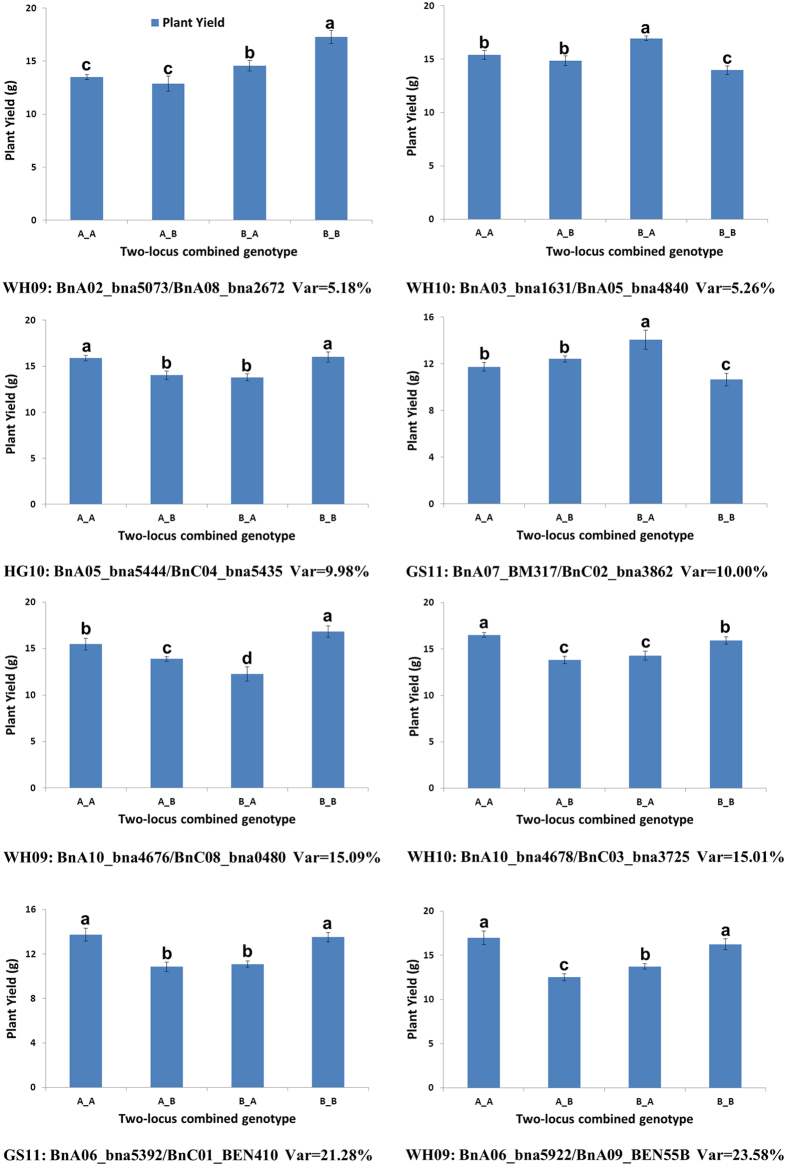
Verification of two-locus interactions for plant yield (PY). The X-axis represents the combination of two-locus genotypes of A (Hua_5) and B (J7005). The Y-axis represents the PY average (g), and the error bars represent the standard error. The letters on each column represent statistically significant difference by multiple comparisons at P = 0.05 level.

To verify the authenticity and effectiveness of the interaction loci-pairs, eight interaction loci-pairs were randomly selected for multiple comparisons between two-locus genotypic combinations of PY (Fig. 4). The average PY values of the 4 different combinations were significantly different (Fig. 4), suggesting that the interaction loci-pairs indeed existed.

**Figure 5:**
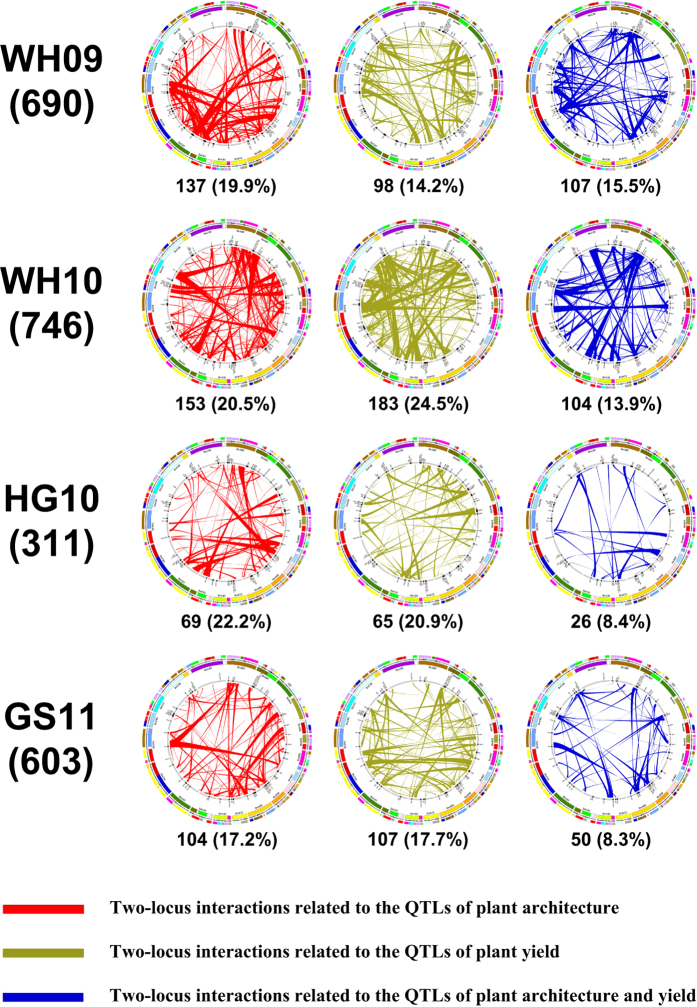
Schematic diagram for epistatic interaction effects of two-locus interactions of plant yield (PY) with quantitative trait loci (QTLs). The left column represents the environment of DH population planting, and the number in each bracket is the two-locus interactions of the PY in the corresponding environment. Each panel represents the two-locus interactions of PY located in or overlapping with the confidence intervals of identified QTLs. The colored blocks in the outermost circle represent the conserved Arabidopsis blocks aligned with the *B. napus* genome identified through SNP/SSR markers in the *B. napus* genetic linkage groups, which are represented by the second outer circle. The third circle represents the *B. napus* genome reconstructed with fragments of homoeologous exchanges defined by Chalhoub *et al.*^27^. The inner circle represents the identified QTLs in the same environment shown in the left column. The red, green, and blue lines represent the interactions related to the QTLs of plant architecture, PY, or both, respectively. The number and percentage in each bracket are the two-locus interactions of the PY corresponding to the colored lines, and the number accounts for the total number (left column) of the two-locus interactions of the PY in the corresponding environment.

To understand the relationship between the interaction loci-pairs and the identified QTLs, we aligned the positions of interaction loci-pairs and mapped QTLs. Approximately half of the interaction loci-pairs, including 342 (49.6%, WH09), 440 (59.0%, WH10), 160 (51.4%, HG10), and 261 (43.3%, GS11), were located within identified QTLs confidence intervals, or overlapped with the intervals (Fig. 5 and Supplementary Table S11), which contained 69 (95.8%), 81 (88.0%), 84 (98.8%), and 95 (97.9%) different QTLs, respectively, covering almost more than 95% QTLs (329/346) in the entire genome. Of these identified QTLs, 38.5% (463/1203) were affected by PA-related traits (average Var = 8.65) (Fig. 5), 37.7% (453/1203) were affected by PY-related traits (average Var = 8.93), and 23.9% (287/1203) were affected by both (average Var = 8.83).

We further analyzed the effects of the interaction loci-pairs corresponding PAFs (MILP, BHP, and BSP) and PY components (PSN, SSN, and TSW) on PY. There were 290 and 363 interaction loci-pairs corresponding PAFs (average Var = 8.53) and PY components traits (average Var = 8.74). These findings further illustrated that PA has a significant influence on PY (accounting for 1/3 of the total effect), and the number and effects of PA-related traits were similar to those of PY-related traits on PY. The effects of the PY interaction loci-pairs corresponding PAFs were equally important to those of the PY components. Interestingly, the interaction loci-pairs were not directly related to QTL effect, and the interaction effect (Var) was also independent from the QTL effect.

For the 183 interaction loci-pairs identified repeatedly in two or more environments, 97 were located in or overlapped with the confidence intervals of 100 identified QTLs (Supplementary Table S12). Of these loci-pairs, 33, 44, and 20 were affected by PA-related traits (average Var = 9.25), PY-related traits (average Var = 8.80), or both (average Var = 9.24), respectively. These results indicate that the contribution of PA-related traits to PY can be more stably identified because the traits are less influenced by the environment.

## Discussion

The improvements of PY in major crops over the last decades have primarily resulted from enhanced photosynthesis efficiency and increased energy conversion efficiency^1^,^2^,^46^, which heavily rely on the continuous optimization of PA-related traits^5^,^6^. Therefore, a better understanding of PA development and genetic regulation has a fundamental influence on the efficiency of high-yield improvement.

As a very complicated trait, rapeseed yield is not only related to the yield-component traits (PSN, SSN, and TSW)^47^, but also influenced by various abiotic and biotic stresses^48^, flowering time^49^, seed germination and seedling vigor^50^. Although it has been recognized for long time that PA is one of the most important factors on crop adaptation and yield potential^1^,^2^, there is a lack of effective measurements in rapeseed that has a relatively loose plant architecture with complex spatial structure. In this study, we proposed the concept of “PAFs” and investigated the relationship between PA and yield. Our results demonstrated that PY is significantly correlated with PA-related traits (Supplementary Table S3). It was showed that PAFs have a direct influence on PY and plot yield by analyzing data from high-yield and low-yield pools in the DH population, which was further verified with data from the China national rapeseed regional trial of winter-type rapeseed area.

Our results and previous studies showed that the three PY components vary from one material to another, which can result in a number of combinations of traits that achieve similar yield levels. This finding imposes a huge challenge on yield breeding for selection of high-yield genotypes. It is important to identify a more universal measurement that can directly reflect yield level of a given crop rather than evaluation of all of a crop’s component traits for seed yield. In our case, PAF seems to be a useful indicator for such a purpose and can be used to predict rapeseed yield level. Compared to the three PY components, PAFs can be easily and accurately measured and can be screened on a large scale in the field. Therefore, PAFs may be widely applied to high-yield breeding in rapeseed and other crops.

PA has been shown to be influenced by environmental factors, such as light, temperature, nutrition, and plant density. In this study, the heritability of PA-related traits exhibited higher than that of PY-related traits (Supplementary Table S2), indicating that PAFs are more stable measurements for genetic manipulation.

Through extensive genetic analysis with a high-density genetic map, we identified 163 unique QTLs related to PA, PY, or both. Analyses of pleiotropic QTLs for controlling PA and yield further showed that PA and PA-related traits interact with PY. Approximately 60% of pleiotropic QTLs exhibited effects on PY and three components of PY through PA-related traits (Supplementary Table S5). Such a finding suggests that we can directly monitor pleiotropic QTLs to improve PY by selecting relevant PAF phenotypes with molecular markers on large scale in practical breeding programs.

We collected 256 genes related to PA development and regulation and grouped the genes into 8 categories. Among the 190 predicted candidate genes corresponding to 91 unique QTLs (Fig. 3 and Supplementary Table S9), it was found that genes in the categories of TF (24.7%), Aux/IAA (22.6%), and GA (13.7%) accounted for the largest proportions, suggesting that these three gene categories are important for PA domestication and improvements in rapeseed, as well as in the process of yield improvement. For instance, *AtCUC2*, *PhNAM*, *LeSP*, and *PsLF* act as TFs in PA development and also indirectly influence seed yield^40^,^41^,^42^,^43^,^44^. The “Green revolution genes” are involved in GA metabolism and signal transduction (e.g., *D8*, *RGA*, and *RHT1*) and play important roles in stem and internode elongation^3^,^4^.

To demonstrate the effectiveness of the prediction of QTL candidate genes, we cloned three candidate genes (Supplementary Figs S5–S7; *Bna.A02.CLV2* for *quPAY.A02_24.39*, *Bna.A09.SLY2* for *quBSP.A09_26.7*, and *Bna.C07.AHK4* for *quPAY.C07_21.9*) from the two parents. The sequences of the three candidate genes were aligned against *B. napus* reference genome and corresponding Arabidopsis genes. These three genes are all related to PA regulation in Arabidopsis^1^,^2^,^51^,^52^,^53^. The *Atclv2* mutation results in fewer siliques, shorter main inflorescence and siliques^51^. The *PsClv2* mutation (*sym28*) has shorter internodes between flowering nodes, and fasciated stem^54^, which is similar to the parent J7005 used in this study (Supplementary Table S1. Bna.A02.CLV2 in J7005 harbored a precocious termination at the 382 aa residues) compared with another parent Hua_5. Similarly, the *Atahk4* mutation leads to a more loose PA with higher branch height and fewer branches^52^, which is similar to the performance of Hua_5 (Supplementary Table S1. Bna.C07.AHK4 in Hua_5 harbored a precocious termination at the 383 aa residues). *AtSLY2* was shown to be related to PA regulation through GA signal^53^. Therefore, the mutations on these genes are consistent with the phenotypic performances of the two parental lines (Fig. 1 and Supplementary Table S1).

PA may vary from one species from another, and such a difference is related to the genetic regulation of important PA-related genes. However, there has not been a systematic study on differentiation, the evolutionary relationship of these PA regulation genes in different species, and the similarities and differences of their regulation in *Brassica* species. Through the comparison of homologous genes in *B. napus*, many important genes for PA formation and regulation were identified, such as *SPY* and *CKI1* reported in Arabidopsis; *RA3* and *TD1* in maize; *TAD1*, *D14*, and *GA2ox1* in rice; and “Green Revolution” genes (*D8*, *RGL1*, and *RHT1*), which had at least one homologous gene in *B. napus* (Supplementary Table S6). However, there were 40 PA-regulating genes, including some important genes, such as *BZR1* and *GAI* in Arabidopsis; *TB1*, *RA1*, and *RA2* in maize; and *DEP1*, *IPA1* (*OsSPL14*), *MOC1*, *TAC1*, and *LA1* in rice, which did not have homologous genes in *B. napus* (Supplementary Table S6). Such a result may be attributed to a small number of homologous genes, low density of the genetic map, or the existence of specific PA-regulating loci in *Brassica*.

In this study, a large number of AA interaction loci-pairs were identified (Supplementary Table S11), suggesting that yield formation is extremely complex. Interaction loci-pairs explained 5.18–23.18% of phenotypic variation. This finding suggests that yield improvement may be achieved by manipulating a few interaction loci, which could further broaden the gene pool for PY improvement in rapeseed.

*B. napus* has a very complex genome structure. In this study, we identified a total of 1818 (77.4%) interaction loci-pairs with conserved blocks in Arabidopsis. Moreover, only 105 (5.78%) interaction loci-pairs have two loci that are both located on different copies of the same conserved block (Supplementary Table S13).There may be an indirect relationship between whole-genome epistatic interactions and different copies of the same conserved blocks in *B. napus*.

Among the 183 interaction loci-pairs detected in two or more environments, 97 were located in or overlapped with confidence intervals of 100 different QTLs, and the QTLs were related to 257 candidate genes (Supplementary Table S12). Interestingly, more than half of the genes fell into three categories of genes, GA (20.2%), TF (17.1%), and Aux/IAA (13.2%), indicating the importance of these three gene categories in plant domestication and PA formation. These three gene categories are worth future in-depth studies in rapeseed.

## Methods

### Plant materials, field trials and trait evaluation

A DH population consisting of 254 individual DH lines was generated from a microspore culture of F1 buds of the cross between cv. Huashuang 5 (Hua_5), a semi-winter *B. napus* variety, and J7005, a pure line with distinct PA from Hua_5. A random subset including 190 lines from the 254 DH lines was sampled as the mapping population (designated as the HJ-DH population thereafter) for linkage map construction and QTL mapping^39^,^55^,^56^.

The DH population together with two parents, F1 and RF1 were planted in Wuhan for two consecutive winter-growing seasons of 2009–2010 (WH09) and 2010–2011 (WH10), in Huanggang in the 2010–2011 (HG10) season, and in Gansu in the spring of 2011 (GS11). The field experiment followed a randomized complete block design with three replicates, including Hua_5 as a control for every 20 plots. Each line was planted in two rows with 12 plants in each row, with distances of 17 cm between plants within each row and 30 cm between rows. Field management followed regular breeding practices.

Fifteen plants growing uniformly from each plot were chosen for trait evaluation. The mean of each trait calculated from the 15 sampled plants in one plot was used to determine plot performance. Traits examined in this study were grouped into two categories. The first category included plant yield (PY) related traits, i.e., silique seed number (SSN), thousand seed weight (TSW), main inflorescence silique number (MISN), branch silique number (BSN), main inflorescence yield (MIY), and branch yield (BY). The second category included PA-related traits, i.e. plant height (PH), branch height (BH), main inflorescence length (MIL), branch number (BN), and branch total length (BTL).

The performance of three high-yield check (CK) cultivars included in the national rapeseed regional trial in China was used to verify the results from the DH population. Each of the CKs were grown in numbers of environment (N) with various plant density (Table 2) following a standard field experiment of a randomized complete block design with three replicates and 20 m^2^ for each plot as outlined in the reports by National Agricultural Technology Extension and Service Center (NATESC)^37^,^57^.

### Statistical analysis

The broad-sense heritability (*h*^2^) of each trait in HJ-DH population was calculated as:


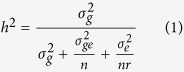


where σ^2^_g_ is genotypic variance, σ^2^_ge_ variance due to genotype by environment interaction, σ^2^_e_ error variance, *n* number of environments; *r* number of replications. The estimates of σ^2^_g_, σ^2^_ge_ and σ^2^_e_, were obtained from an ANOVA with environment considered as a random effect.

The statistical analysis of the phenotypes (correlation coefficient, multiple comparison) was conducted through the Excel 2010 and SAS 7.0 software.

### Molecular marker and SNP array genotyping

Primer sequences of SSR (simple sequence repeat) markers for genetic mapping were described by Cai *et al.*^39^. SNP genotyping was performed using *B. napus* 6 K Illumina Infinium HD Assay SNP arrays (Illumina Inc., San Diego, CA) developed by the University of Queensland. The analysis process and bi-filtering method were described previously by Cai *et al.*^56^.

### Genetic map construction, QTL mapping and meta-analysis

Linkage analysis with all markers was performed using MAPMAKER 3.0^58^ and MSTmap^59^ software. Detailed information describing genetic map construction was provided previously by Cai *et al.*^39^. QTLs were detected using the composite interval mapping (CIM) procedure in QTL Cartographer V2.5 software (http://statgen.ncsu.edu/qtlcart/WQTLCart.htm). A P = 0.05 significance threshold for QTLs was determined through permutation analysis with 1,000 repetitions. Other parameters and QTL mapping methods were described previously by Feng *et al.*^60^.

Identified QTLs were integrated using a “two-round” strategy of QTL meta-analysis^60^,^61^ with BioMercator2.1 software^62^,^63^. In the first round, identified QTLs of a specific trait that were repeatedly detected in multiple environments were integrated and labeled as consensus QTLs (step 1). In the second round, overlapping consensus QTLs of different traits were integrated into unique QTLs (step 2).

### Identification of homologous PA-related genes in *B. napus*

To identify and locate putative PA-related homologous genes in *B. napus*, the CDS sequences of PA-related genes in Arabidopsis were used as queries to search for homologous genes using BLASTn program, and the protein sequences of PA-related genes in other species were queried against the *B. napus* genome^27^ (http://www.genoscope.cns.fr/brassicanapus/) using the BLASTp program with an E-value of 1E-50 and an Identity of 50% set as thresholds.

### Identification of physical SSR and SNP marker positions in *B. napus*

An SSR primer sequence was used to identify its physical position in the *B. napus* reference genome^27^ through e-PCR analysis. When an SSR marker had more than two amplicons, the accurate position for a particular locus was manually determined by referring to the physical positions of its upstream and downstream amplicons^64^.

The probe sequence of a SNP was used to identify its physical position in the *B. napus* reference genome using BLASTn analysis. The best-hit locus of BLASTn results was chosen as the potential physical position of the given SNP locus in *B. napus*.

The method used to identify homoeologous exchanges described by Chalhoub *et al.*^27^ was performed as described previously by Cai *et al.*^39^. The relationship between the *B. napus* genetic map and the physical map (reference genome) was established (Supplementary Table S7) by homoeologous exchanges identified in the HJ-DH population.

The homologs of PA-related genes in *B. napus* were mapped onto *B. napus* genetic linkage groups based on the physical positions of the markers and homologous genes.

### Genome-wide epistatic interaction analysis

Genome-wide epistatic interaction analysis was conducted using two-way analysis of variance (ANOVA) as described by Zhou *et al.*^45^. Briefly, a statistic for the F-test was first constructed as:


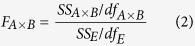


where *F*_*A × B*_ is the F-test for the significance of interaction effect of locus-pair (A locus and B locus). *SS*_*A × B*_ and *SS*_*E*_ are the sum of squares of the AB locus-pair interaction effect and the random error effect, respectively. *df*_*A × B*_ and *df*_*E*_ are the degree of freedom of the AB locus-pair interaction effect and random error effect, respectively. A significance threshold for the F-test at P ≤ 0.001 was calculated through 10,000- repetition permutation analysis. The details of epistatic interaction loci detection have been described previously by Zhou *et al.*^45^.

The two-locus-interaction significant pairs that passed the permutation tests and significance on F-test, were used to merge the interaction effects of the neighboring molecular markers by using of point-to-point expansion method. The two interaction loci were ≥defined as *A*_*x*_ and *B*_*y*_, and the F-value was calculated as *F(x, y)* by two-way ANOVA, and then a *M × N* matrix was constructed:


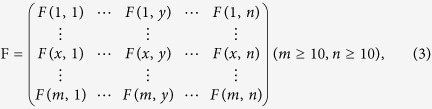


We used the *A*_*x*_ and *B*_*y*_ as the centers, and extended from these two centers in two directions by *m* and *n* marker loci (*m* ≥ 10, *n* ≥ 10), respectively. The distance between *A*_*1*_ and *A*_*m*_, *B*_*1*_ and *B*_*n*_ was not less than 2 cM.

The largest effect locus *F(x, y)* was chosen in the matrix as the starting point to perform a merging process. We first fixed *A*_*x*_, and used *B*_*y*_ as the center, extended towards the two ends to test the significance of the molecular markers within the region from *B*_*1*_ to *B*_*n*_ with *A*_*x*_. The molecular markers in the *B* region were merged using a threshold 1.5-*Lg*_*10*_*P*-drop confidence interval, and these merged markers were defined as *A*_*x′*_. And then we fixed *B*_*y*_, and extended the loci towards the two ends, centering on the *A*_*x*_, to test the significance of the molecular markers in the *A*_*1*_ to *A*_*m*_ region with *B*_*y*_. Similarly, the molecular markers in this *A* region were merged using a threshold 1.5-*Lg*_*10*_*P*-drop confidence interval, and they were defined as *B*_*y′*_. Finally, the combination *A*_*x′*_ × *B*_*y′*_ consisting of two groups of markers from regions *A* and *B* were regarded as one epistatic interaction pair, the effect of which come from the most significant pair *A*_*x*_ × *B*_*y*_ in all the corresponding pairs.

### Gene cloning and sequence alignment

Genomic DNA was prepared from young leaf tissues as described previously^39^. The primers used for amplification of the genomic DNA fragments are listed in supplementary Table S14. The PCR products were cloned into pMD18-T vector (Takara Corporation, Japan) according to the manufacturer’s instructions. The M13F and M13R universal primers and the BigDye Terminator Cycle Sequencing v3.1 (Applied Biosystems, Foster City, CA, USA) were used for sequencing. Sequences were aligned using SEQUENCHER 4.1.2 (Gene Codes Corporation, Ann Arbor, MI, USA).

## Additional Information

**How to cite this article**: Cai, G. *et al.* Genetic dissection of plant architecture and yield-related traits in *Brassica napus*. *Sci. Rep.* **6**, 21625; doi: 10.1038/srep21625 (2016).

## Supplementary Material

Supplementary Information

Supplementary dataset 1

Supplementary dataset 2

Supplementary dataset 3

Supplementary dataset 4

Supplementary dataset 5

Supplementary dataset 6

Supplementary dataset 7

## Figures and Tables

**Table 1:** Statistics of PA- and PY-related traits in high- and low-yield pools in four environments.

Env.	Material	N	AVE ± SD^a^									
			PY (g)	BHP	MILP	BSP	BN	BTL (cm)	BAL (cm)	PSN	SSN (per silique)	TSW (g)
WH09	High-PY Pool	25	19.21 ± 2.53 a^b^	0.376 ± 0.050 b	0.376 ± 0.024 b	0.248 ± 0.036 a	8.1 ± 0.9 a	464.3 ± 70.8 a	57.2 ± 4.1 a	417.6 ± 66.7 a	15.0 ± 3.4 a	3.161 ± 0.405 b
	Low-PY Pool	27	10.53 ± 1.35 b	0.409 ± 0.053 a	0.402 ± 0.036 a	0.189 ± 0.023 b	5.9 ± 0.5 c	304.6 ± 42.0 c	51.4 ± 4.9 b	270.5 ± 40.2 b	14.3 ± 2.6 a	2.798 ± 0.400 c
	Hua_5	35	17.88 ± 4.15 a	0.381 ± 0.038 b	0.383 ± 0.026 b	0.236 ± 0.031 a	7.1 ± 0.7 b	408.1 ± 68.3 b	57.5 ± 5.3 a	285.5 ± 50.3 b	14.9 ± 2.7 a	4.241 ± 0.249 a
WH10	High-PY Pool	34	19.07 ± 0.96 a	0.367 ± 0.060 a	0.367 ± 0.057 b	0.265 ± 0.028 b	8.9 ± 1.2 a	620.7 ± 62.4 a	70.3 ± 8.1 ab	439.7 ± 48.3 a	19.4 ± 3.3 a	2.462 ± 0.389 b
	Low-PY Pool	16	10.69 ± 1.10 b	0.273±0.072 c	0.464 ± 0.079 a	0.263 ± 0.027 b	7.2 ± 1.2 b	536.1 ± 51.6 b	76.0 ± 9.8 a	341.7 ± 61.4 b	14.8 ± 3.0 b	2.545 ± 0.412 b
	Hua_5	33	19.35 ± 3.38 a	0.327±0.061 b	0.380 ± 0.042 b	0.293 ± 0.042 a	7.9 ± 0.9 b	530.6 ± 129.0 b	67.5 ± 13.4 b	307.9 ± 62.4 b	20.2 ± 1.3 a	3.565 ± 0.229 a
HG10	High-PY Pool	20	20.09 ± 1.91 a	0.324 ± 0.061 a	0.392 ± 0.044 c	0.284 ± 0.040 ab	8.4 ± 1.2 a	629.5 ± 101.8 a	75.0 ± 5.9 a	394.4 ± 56.8 a	21.1 ± 2.1 ab	2.417 ± 0.373 b
	Low-PY Pool	43	11.52 ± 1.37 c	0.288 ± 0.046 b	0.435 ± 0.040 b	0.277 ± 0.031 b	7.1 ± 0.7 b	515.7 ± 57.8 b	72.5 ± 7.2 a	277.3 ± 46.0 b	17.5 ± 3.4 c	2.569 ± 0.373 b
	Hua_5	26	17.35 ± 3.53 b	0.306 ± 0.049 ab	0.400 ± 0.033 c	0.294 ± 0.035 a	6.9 ± 0.8 b	490.6 ± 80.7 b	70.7 ± 6.1 a	239.2 ± 44.2 c	21.3 ± 1.0 a	3.424 ± 0.238 a
GS11	High-PY Pool	28	16.73 ± 1.67 a	0.302 ± 0.062 a	0.460 ± 0.065 b	0.237 ± 0.033 b	6.4 ± 0.8 a	403.0 ± 42.3 a	63.5 ± 5.5 a	309.1 ± 46.9 a	20.5 ± 3.2 b	2.932 ± 0.253 b
	Low-PY Pool	27	7.97 ± 0.90 b	0.251 ± 0.049 b	0.532 ± 0.044 a	0.217 ± 0.032 c	5.1 ± 0.5 b	323.1 ± 43.8 b	63.5 ± 6.7 a	236.6 ± 60.4 b	16.6 ± 5.4 c	3.008 ± 0.372 b
	Hua_5	26	16.52 ± 3.88 a	0.269 ± 0.078 ab	0.470 ± 0.066 b	0.262 ± 0.038 a	5.9 ± 0.8 a	384.8 ± 61.3 a	64.9 ± 7.3 a	217.7 ± 45.0 b	23.0 ± 1.7 a	3.963±0.365 a

^a^Average ± standard deviation.

^b^The lowercase letter indicates a significant difference at the 0.05 probability level based on Duncan-test among the different materials of the same trait.

**Table 2:** Performances of the plant architecture and yield related traits of the check (CK) varieties in China National Regional Trial materials in the years of 2006–2010.

District^a^	Material	Yea	N	Density^b^	Plot yield (kg)^c^	Plant yield (g)	BHP^d^	MILP^d^	BSP^d^	BN^d^	PSN^d^	SSN^d^	TSW (g)^d^
U-Y. R.	Youyan_10	2006	12	5.3–10	4.730 ± 0.102	22.59 ± 0.67	0.421 ± 0.020	0.301±0.010	0.283 ± 0.029	9.3 ± 0.4	418.1 ± 17.2	18.2 ± 0.6	3.405 ± 0.106
		2007	12	5.3–10	4.928 ± 0.106	24.82 ± 0.51	0.403 ± 0.012	0.314 ± 0.010	0.283 ± 0.012	8.5 ± 0.3	444.3 ± 14.0	18.3 ± 0.5	3.637 ± 0.232
		2008	12	5.3–10	4.829 ± 0.014	23.85 ± 0.67	0.396 ± 0.008	0.329 ± 0.007	0.275 ± 0.003	9.6 ± 0.1	450.9 ± 15.8	17.5 ± 0.3	3.360 ± 0.095
		2009	10	5.3–10	5.083 ± 0.098	28.09 ± 2.00	0.364 ± 0.013	0.347 ± 0.004	0.289 ± 0.014	8.7 ± 0.1	483.5 ± 9.6	16.9 ± 0.3	3.857 ± 0.257
		2010	12	15–20	5.424 ± 0.194	22.95 ± 0.58	0.391 ± 0.007	0.346 ± 0.007	0.263 ± 0.003	8.7 ± 0.2	452.2 ± 14.6	17.3 ± 0.4	3.638 ± 0.035
M–Y. R.	Zhongyouza_2	2006	10	10–12	5.830 ± 0.201	21.62 ± 0.50	0.424 ± 0.019	0.301 ± 0.006	0.274 ± 0.025	8.0 ± 0.1	323.6 ± 14.9	21.5 ± 0.9	3.747 ± 0.090
		2007	10	10–12	4.747 ± 0.052	17.99 ± 0.43	0.378 ± 0.003	0.390 ± 0.016	0.232 ± 0.014	7.5 ± 0.2	298.7 ± 12.6	19.0 ± 0.4	3.910 ± 0.010
		2008	9	10–12	4.663 ± 0.064	17.25 ± 1.47	0.406 ± 0.010	0.334 ± 0.022	0.260 ± 0.014	7.4 ± 0.1	276.4 ± 16.2	20.5 ± 0.6	4.070 ± 0.030
		2009	10	10–12	4.912 ± 0.121	17.29 ± 0.61	0.429 ± 0.009	0.335 ± 0.004	0.236 ± 0.012	7.4 ± 0.3	296.1 ± 14.9	18.3 ± 0.6	3.933 ± 0.116
		2010	10	15–20	5.132 ± 0.226	13.02 ± 0.56	0.429 ± 0.013	0.359 ± 0.009	0.212 ± 0.014	5.9 ± 0.3	222.7 ± 14.9	19.8 ± 0.7	3.838 ± 0.056
L–Y. R.	Qinyou_7	2006	9	8–10	5.486 ± 0.345	30.51 ± 0.95	0.269 ± 0.022	0.357 ± 0.009	0.374 ± 0.013	8.4 ± 0.2	428.7 ± 8.5	22.5 ± 0.5	3.707 ± 0.110
		2007	8	8–10	4.994 ± 0.283	31.73 ± 1.46	0.259 ± 0.032	0.398 ± 0.009	0.329 ± 0.025	7.8 ± 0.4	388.4 ± 35.3	23.8 ± 0.9	3.930 ± 0.062
		2008	8	8–10	4.659 ± 0.161	29.06 ± 0.46	0.278 ± 0.016	0.391 ± 0.013	0.331 ± 0.029	7.8 ± 0.3	389.3 ± 8.3	23.3 ± 1.1	3.540 ± 0.040
		2009	8	8–10	4.769 ± 0.096	23.76 ± 0.17	0.230 ± 0.015	0.398 ± 0.015	0.372 ± 0.021	7.4 ± 0.3	353.3 ± 20.9	23.3 ± 0.6	3.610 ± 0.070
		2010	9	15–20	4.898 ± 0.216	19.58 ± 1.97	0.324 ± 0.010	0.368 ± 0.022	0.308 ± 0.019	6.8 ± 0.4	267.1 ± 12.0	24.7 ± 1.1	3.243 ± 0.050
HuangHuai	Qinyou_7	2006	9	8.4–12	5.512 ± 0.115	20.50 ± 1.08	0.423 ± 0.013	0.392 ± 0.003	0.185 ± 0.010	7.9 ± 0.3	351.0 ± 7.6	20.4 ± 1.2	3.400 ± 0.081
		2007	10	8.4–12	6.710 ± 0.009	25.19 ± 0.76	0.319 ± 0.024	0.366 ± 0.001	0.315 ± 0.024	8.2 ± 0.0	327.2 ± 5.5	22.2 ± 0.7	3.860 ± 0.099
		2008	10	8.4–12	5.482 ± 0.077	19.56 ± 0.29	0.302 ± 0.010	0.353 ± 0.000	0.345 ± 0.010	8.7 ± 0.0	316.6 ± 2.5	20.6 ± 0.6	3.460 ± 0.014
		2009	11	8.4–12	5.357 ± 0.134	21.15 ± 0.92	0.284 ± 0.008	0.358 ± 0.002	0.358 ± 0.006	8.3 ± 0.4	324.4 ± 10.3	22.6 ± 0.2	3.325 ± 0.035
		2010	11	15–20	6.398 ± 0.006	19.34 ± 0.04	0.330 ± 0.018	0.341 ± 0.011	0.329 ± 0.029	8.0 ± 0.0	295.6 ± 0.3	24.2 ± 0.3	3.145 ± 0.021

^a^The abbreviations of the district mean: U-Y. R.: Upper reaches of Yangtze River; M-Y. R.: Middle reaches of Yangtze River; L-Y. R.: Lower reaches of Yangtze River.

^b^Thousand plants per 667 m^2^.

^c^The plot area is 20 m^2^.

^d^Abbreviations: BHP: branch height proportion; MILP: main inflorescence length proportion; BSP: branch segment proportion; BN: branch number; PSN: plant silique number; SSN: silique seed number; TSW: thousand seed weight.

**Table 3:** Identified QTLs, consensus QTLs, and unique QTLs.

LG	Identified QTL	Consensus QTL	Unique QTL	LG	Identified QTL	Consensus QTL	Unique QTL
BnA01	23	21	10	BnC01	9	7	5
BnA02	42	32	13	BnC02	13	11	8
BnA03	23	22	16	BnC03	12	11	8
BnA04	11	9	5	BnC04	28	27	14
BnA05	5	5	4	BnC05	12	12	5
BnA06	9	8	6	BnC06	24	19	9
BnA07	14	14	11	BnC07	15	14	9
BnA08	29	25	10	BnC08	29	25	11
BnA09	21	15	7	BnC09	10	10	7
BnA10	17	14	5				
Sub total	194	165	87	Sub total	152	136	76
Total					346	301	163

**Table 4:** Identified QTLs of the 20 plant architecture and yield related traits of the HJ-DH population in four environments.

Trait	Env.	N.	LOD (min-max)	*R*^*2*^ (sum)	*R*^*2*^ (min-max)	A (+)	A (−)	Consensus QTL	Trait	Env.	N.	LOD (min-max)	*R*^*2*^ (sum)	*R*^*2*^ (min-max)	A (+)	A (−)	Consensus QTL
PH	WH09	4	5.74–8.15	40.0%	8.3–12.1%	3.4 (1)	−8.5 (3)	20	MISN	WH09	4	5.02–14.66	37.1%	5.3–18.7%	5.0 (2)	−8.0 (2)	16
	WH10	5	3.57–10.48	44.1%	5.2–16.0%	9.2 (2)	−15.7 (3)			WH10	7	3.51–19.08	86.7%	5.0–34.2%	13.9 (5)	−8.7 (2)	
	HG10	5	4.62–14.05	56.0%	5.8–19.2%	12.0 (3)	−8.1 (2)			HG10	6	3.51–13.95	60.1%	6.0–17.5%	14.5 (5)	−3.9 (1)	
	GS11	7	3.50–17.03	63.2%	3.7–23.8%	9.0 (3)	−15.7 (4)			GS11	4	3.98–9.86	36.6%	5.8–14.8%	7.6 (3)	−3.2 (1)	
MIL	WH09	3	4.25–9.39	40.1%	7.3–18.6%	3.6 (2)	−2.6 (1)	16	BSN	WH09	4	4.24–9.99	36.5%	6.2–15.8%		−92.2 (4)	13
	WH10	9	3.28–27.42	96.8%	3.8–51.8%	14.5 (5)	−8.3 (4)			WH10	3	4.01–7.95	28.9%	6.7–13.9%		−57.6 (3)	
	HG10	3	6.81–8.56	35.4%	10.7–13.7%	4.2 (2)	−2.0 (1)			HG10	7	3.21–6.96	67.9%	6.6–13.4%	19.6 (1)	−110.5 (6)	
	GS11	4	6.30–8.10	34.2%	7.8–9.6%	7.2 (3)	−2.4 (1)			GS11	1	13.48	30.4%	30.4%		−153.6 (1)	
BH	WH09	5	3.89–14.49	63.7%	7.6–25.7%	6.7 (2)	−10.2 (3)	16	PSN	WH09	4	3.72–12.41	48.9%	6.3–23.7%		−112.7 (4)	11
	WH10	5	5.79–22.78	60.0%	6.7–29.0%	5.8 (1)	−24.8 (4)			WH10	3	4.27–11.54	39.5%	6.7–19.7%		−79.2 (3)	
	HG10	6	3.87–8.07	45.4%	5.5–11.2%	11.3 (3)	−11.1 (3)			HG10	4	3.86–9.07	39.8%	6.5–15.7%	16.8 (1)	−66.4 (3)	
	GS11	3	7.20–26.44	46.2%	6.2–33.4%	2.9 (1)	−9.1 (2)			GS11	2	3.13–13.17	35.4%	6.3–29.1%		−174.0 (2)	
BS	WH09	3	3.54–7.96	24.1%	5.5–11.6%		−6.0 (3)	13	MISNP	WH09	3	5.57–9.80	31.9%	7.1–13.2%	0.050 (3)		9
	WH10	3	3.84–6.15	31.2%	7.4–13.6%	2.1 (1)	−4.3 (2)			WH10	3	3.78–6.31	26.3%	6.9–12.5%	0.007 (1)	−0.018 (2)	
	HG10	4	5.45–7.97	39.0%	7.5–11.4%	4.1 (2)	−11.0 (2)			HG10	2	3.05–3.28	12.6%	6.2–6.3%	0.017 (2)		
	GS11	3	3.71–7.39	25.5%	5.6–11.5%	3.4 (2)	−1.9 (1)			GS11	1	4.33	7.6%	7.6%	0.012 (1)		
BHP	WH09	4	4.08–9.56	36.0%	5.6–14.0%	0.033 (2)	−0.036 (2)	17	SSN	WH09	3	5.58–7.21	31.4%	9.4–12.0%	33.8 (3)		17
	WH10	6	3.47–22.34	64.9%	3.5–28.3%	0.040 (2)	−0.112 (4)			WH10	6	3.48–11.53	66.5%	6.4–25.1%	50.0 (4)	−21.0 (2)	
	HG10	6	3.54–6.96	40.9%	3.8–9.0%	0.034 (2)	−0.063 (4)			HG10	7	4.45–13.61	69.4%	5.6–21.6%	59.4 (5)	−18.9 (2)	
	GS11	5	3.30–30.24	53.2%	2.8–37.7%	0.042 (3)	−0.054 (2)			GS11	7	3.80–11.97	95.0%	7.8–26.4%	65.4 (4)	−44.7 (3)	
MILP	WH09	4	3.44–14.65	56.5%	4.3–20.8%	0.049 (3)	−0.009 (1)	18	TSW	WH09	10	3.69–15.08	71.4%	4.2–13.6%	0.674 (5)	−0.602 (5)	22
	WH10	6	3.77–20.76	54.1%	4.1–28.1%	0.083 (3)	−0.057 (3)			WH10	5	4.54–8.77	47.4%	7.3–11.3%	0.389 (3)	−0.279 (2)	
	HG10	6	3.43–16.50	54.5%	3.6–23.5%	0.050 (4)	−0.027 (2)			HG10	6	3.78–13.38	57.5%	3.4–15.4%	0.482 (4)	−0.237 (2)	
	GS11	5	4.13–25.49	41.9%	2.9–22.5%	0.065 (3)	−0.037 (2)			GS11	5	4.76–10.66	45.5%	4.7–13.5%	0.366 (3)	−0.248 (2)	
BSP	WH09	6	3.45–11.16	57.3%	5.2–19.0%	0.024 (2)	−0.055 (4)	15	BY	WH09	4	4.33–5.72	33.9%	6.8–9.2%	1.11 (1)	−3.05 (3)	11
	WH10	2	4.32–7.67	23.7%	8.6–15.1%	0.011 (1)	−0.019 (1)			WH10	4	3.50–7.10	33.9%	5.3–11.4%	2.39 (3)	−0.59 (1)	
	HG10	4	3.51–16.95	52.2%	4.9–28.1%	0.024 (2)	−0.029 (2)			HG10	3	4.02–5.33	27.8%	7.9–10.8%	1.76 (2)	−0.84 (1)	
	GS11	3	6.03–7.94	38.9%	11.8–15.2%	0.015 (1)	−0.027 (2)			GS11	1	11.81	20.8%	20.8%		−1.37 (1)	
BN	WH09	5	4.98–7.74	31.3%	4.9–8.6%	0.3 (1)	−1.2 (4)	15	PY	WH09	4	3.56–12.34	51.2%	5.6–21.9%	2.34 (2)	−2.83 (2)	16
	WH10	4	4.16–27.91	61.7%	4.4–40.7%	0.5 (1)	−1.6 (3)			WH10	9	3.23–9.38	71.5%	4.6–14.4%	6.12 (7)	−1.46 (2)	
	HG10	5	3.91–8.10	48.5%	5.8–12.8%	0.3 (1)	−1.8 (4)			HG10	2	4.36–8.14	24.7%	8.1–16.6%	2.48 (2)		
	GS11	6	4.35–21.89	78.4%	4.7–33.6%	0.3 (1)	−1.6 (5)			GS11	1	8.17	14.6%	14.6%		−1.27 (1)	
BTL	WH09	4	3.47–8.94	29.0%	4.3–11.3%		−96.6 (4)	10	MIY	WH09	3	5.35–9.64	42.2%	8.2–19.9%	0.76 (3)		18
	WH10	3	4.88–9.23	33.3%	7.5–15.9%		−83.9 (3)			WH10	6	3.57–9.52	53.4%	4.8–13.6%	1.13 (5)	−0.22 (1)	
	HG10	3	3.65–8.86	28.1%	5.9–15.3%		−113.8 (3)			HG10	5	4.08–7.13	49.5%	6.4–11.4%	1.28 (5)		
	GS11	2	5.06–8.43	25.6%	9.2–16.4%		−40.8 (2)			GS11	5	4.39–8.83	42.1%	5.6–11.9%	0.71 (3)	−0.58 (2)	
BAL	WH09	3	4.02–10.31	31.4%	6.5–16.9%	2.7 (1)	−3.3 (2)	15	MIYP	WH09	5	3.55–9.46	58.6%	5.0–16.8%	0.121 (5)		13
	WH10	6	4.18–24.08	75.2%	3.7–28.5%	14.4 (3)	−9.0 (3)			WH10	2	3.54–4.89	17.0%	7.5–9.6%	0.024 (2)		
	HG10	5	3.63–6.57	48.0%	6.6–13.7%	5.6 (2)	−7.9 (3)			HG10	3	4.24–5.42	28.9%	7.8–11.6%	0.023 (2)	−0.010 (1)	
	GS11	4	3.48–9.19	34.5%	5.3–15.1%	4.3 (2)	−3.7 (2)			GS11	3	4.11–17.01	41.1%	5.2–25.9%	0.037 (2)	−0.035 (1)	
Total		179				73	106	155			167				99	68	146

**Table 5:** Homologous genes of plant architecture regulation in *B. napus* genetic map.

Category	Pathway								Total
	Aux/IAA	CK	GA	BR	SL	CC	TF	Other	
Gene (At)	40 (34)	22 (20)	42 (28)	25 (10)	22 (7)	26 (25)	37 (22)	42 (17)	256 (163)
*Bn*	198 (175)	101 (72)	180 (116)	117 (51)	68 (18)	100 (91)	189 (95)	127 (54)	1080 (672)
BnA01	1 (0)	1 (1)	2 (2)	6 (3)	2 (1)	8 (7)	7 (3)	4 (1)	31 (18)
BnA02	6 (6)	2 (2)	12 (8)	6 (3)	0 (0)	0 (0)	13 (10)	6 (1)	45 (30)
BnA03	15 (12)	5 (4)	8 (7)	9 (6)	11 (1)	14 (11)	25 (11)	9 (4)	96 (56)
BnA04	1 (1)	3 (2)	2 (1)	0 (0)	0 (0)	1 (1)	0 (0)	2 (2)	9 (7)
BnA05	3 (2)	1 (1)	7 (3)	2 (1)	0 (0)	0 (0)	4 (1)	4 (1)	21 (9)
BnA06	10 (8)	9 (9)	7 (3)	4 (2)	0 (0)	3 (3)	8 (6)	8 (4)	49 (35)
BnA07	16 (15)	6 (5)	8 (5)	5 (1)	4 (0)	4 (4)	10 (1)	4 (2)	57 (33)
BnA08	9 (9)	2 (1)	4 (4)	6 (3)	5 (1)	1 (1)	5 (3)	1 (0)	33 (22)
BnA09	8 (6)	5 (3)	11 (6)	5 (1)	0 (0)	4 (3)	6 (2)	9 (3)	48 (24)
BnA10	7 (7)	3 (2)	8 (4)	3 (1)	3 (1)	0 (0)	10 (6)	2 (0)	36 (21)
Sub total	76 (67)	37 (30)	69 (43)	46 (21)	25 (4)	35 (30)	88 (43)	49 (18)	425 (255)
BnC01	3 (3)	0 (0)	5 (4)	6 (2)	0 (0)	4 (3)	3 (2)	3 (1)	24 (15)
BnC02	4 (4)	4 (4)	8 (7)	1 (0)	0 (0)	1 (1)	6 (4)	3 (0)	27 (20)
BnC03	8 (5)	6 (4)	4 (4)	4 ((3)	2 (0)	4 (4)	13 (9)	4 (2)	45 (31)
BnC04	13 (11)	9 (2)	5 (4)	6 (4)	11 (2)	4 (4)	14 (3)	12 (8)	74 (38)
BnC05	16 (16)	2 (1)	8 (8)	4 (2)	9 (2)	4 (4)	7 (4)	8 (5)	58 (42)
BnC06	7 (7)	3 (3)	4 (3)	2 (0)	0 (0)	2 (2)	1 (1)	4 (2)	23 (18)
BnC07	4 (4)	6 (6)	14 (8)	7 (3)	0 (0)	8 (7)	8 (2)	8 (2)	55 (32)
BnC08	18 (18)	3 (2)	5 (2)	4 (1)	5 (2)	4 (4)	1 (1)	6 (6)	46 (36)
BnC09	10 (7)	5 (3)	11 (6)	5 (1)	0 (0)	4 (3)	8 (5)	6 (1)	49 (26)
Sub total	83 (75)	38 (25)	64 (46)	39 (16)	27 (6)	35 (32)	61 (31)	54 (27)	401 (258)
Total	159 (142)	75 (55)	133 (89)	85 (37)	52 (10)	70 (62)	149 (74)	103 (45)	826 (513)

The number in the brackets means the numbers of the Arabidopsis genes or the homologous genes of the Arabidopsis in *B. napus*.
