# Supplementary material for: Genetic dissection of plant architecture and yield-related traits in Brassica napus
Source: Sci Rep. 2016 Feb 16;6:21625. doi: 10.1038/srep21625 (PMC4754947; doi:10.1038/srep21625)
Supplement: Supplementary Information [file srep21625-s1.pdf]

Genetic dissection of plant architecture and yield-related traits in *Brassica napus*

Guangqin Cai[1, 2], Qingyong Yang[1], Hao Chen[1], Qian Yang[2], Chunyu Zhang[2], Chuchuan Fan[1], Yongming Zhou[1, 2, *]

[1]National Key Laboratory of Crop Genetic Improvement, Huazhong Agricultural University, Wuhan 430070, China

[2]Key Laboratory of Rapeseed Genetics and Breeding of Agriculture Ministry of China, Huazhong Agricultural University, Wuhan 430070, China

[*]Corresponding author: Tel +86 (0)27 8728 6869; fax +86 (0)27 8728 1009; email ymzhou@mail.hzau.edu.cn

Additional Information

Competing financial interests

The authors have no competing interests as defined by Nature Publishing Group, or other interests that might be perceived to influence the results and/or discussion reported in this paper.

Supplementary Information

Supplementary Table S1. Performances of 20 evaluated plant architecture and yield related traits of two parents, F1, RF1, parental mean, and DH population mean, and range in the HJ-DH population in four environments.

Trait	Env.	Hua 5 [a]	F1	RF1	J 7005	Parental mean	DH mean	DH range
**PH (cm)**	WH09	161.1±6.9 ab [b]	166.8±13.1 a	152.1±5.4 bc	142.4±1.4 c	151.8±4.1 bc	147.4±7.6 c	122.5-168.3
	WH10	163.3±19.1 a	161.3±1.0 a	170.9±6.2 a	167.2±9.3 a	164.5±13.7 a	172.7±14.4 a	132.9-213.2
	HG10	169.8±12.7 a	180.9±9.5 a	177.8±8.5 a	169.3±5.4 a	169.6±6.2 a	171.8±10.3 a	148.8-201.4
	GS11	138.8±10.8 a	137.2±14.9 a	142.9±12.3 a	131.5±6.1 a	135.2±7.2 a	132.8±10.7 a	107.6-170.5
**BH (cm)**	WH09	52.4±15.0 a	59.0±6.9 a	48.3±15.3 a	54.9±16.5 a	53.7±9.4 a	56.7±7.8 a	38.5-75.6
	WH10	41.2±10.0 b	40.7±10.6 b	40.7±14.4 b	59.1±13.2 a	50.2±9.8 ab	59.4±16.4 a	21.7-98.9
	HG10	42.1±10.8 a	54.6±16.9 a	52.7±6.5 a	51.4±10.6 a	46.8±7.0 a	54.7±11.6 a	30.8-88.9
	GS11	31.1±9.7 b	34.3±14.8 ab	37.3±11.8 ab	45.7±9.6 a	38.4±7.4 ab	36.6±10.5 ab	18.5-66.5
**MIL (cm)**	WH09	68.0±4.4 a	62.5±7.5 ab	60.8±2.6 ab	44.6±5.6 c	56.3±3.9 b	57.7±5.2 b	44.1-77.1
	WH10	81.1±7.1 a	70.5±7.0 ab	69.2±4.6 b	49.9±9.3 c	65.0±4.3 b	67.3±7.6 b	44.3-88.9
	HG10	81.0±8.9 a	73.2±3.8 b	69.6±1.3 b	53.2±2.7 c	67.1±4.4 b	70.8±5.2 b	50.7-87.6
	GS11	76.0±4.4 a	68.3±3.1 b	67.0±4.5 b	50.7±4.7 d	63.3±3.5 c	65.8±6.9 bc	49.2-80.0
**BS (cm)**	WH09	40.7±12.0 a	45.4±7.5 a	43.1±7.6 a	42.8±12.5 a	41.8±9.2 a	33.0±6.3 a	20.3-52.0
	WH10	41.0±7.3 b	50.2±2.6 ab	61.0±7.8 a	58.2±6.3 a	49.3±5.3 b	46.0±6.4 b	30.6-65.3
	HG10	46.7±7.6 c	53.2±10.1 bc	55.5±3.0 b	64.7±5.8 a	55.7±5.0 ab	46.2±5.7 c	35.8-71.5
	GS11	31.8±3.8 b	34.6±1.2 a	38.6±6.6 a	35.2±5.7 ab	33.5±3.6 b	30.5±5.2 b	19.6-47.4
**BHP**	WH09	0.329±0.096 a	0.358±0.053 a	0.315±0.090 a	0.389±0.122 a	0.359±0.070 a	0.386±0.050 a	0.262-0.485
	WH10	0.250±0.045 c	0.252±0.064 bc	0.237±0.082 c	0.353±0.072 a	0.303±0.043 abc	0.339±0.073 ab	0.160-0.508
	HG10	0.247±0.057 a	0.300±0.083 a	0.296±0.026 a	0.302±0.056 a	0.275±0.038 a	0.316±0.055 a	0.181-0.442
	GS11	0.221±0.058 c	0.245±0.079 bc	0.260±0.069 bc	0.346±0.063 a	0.284±0.045 ab	0.270±0.061 abc	0.146-0.436
**MILP**	WH09	0.422±0.030 a	0.374±0.030 ab	0.401±0.031 a	0.313±0.037 b	0.367±0.018 ab	0.392±0.033 a	0.283-0.504
	WH10	0.500±0.044 a	0.437±0.046 ab	0.406±0.036 b	0.299±0.054 c	0.399±0.037 b	0.395±0.071 b	0.228-0.601
	HG10	0.477±0.036 a	0.405±0.036 b	0.392±0.019 b	0.315±0.019 c	0.396±0.019 b	0.414±0.039 b	0.280-0.521
	GS11	0.549±0.033 a	0.502±0.061 b	0.470±0.028 b	0.385±0.031 c	0.467±0.017 b	0.501±0.065 b	0.347-0.629
**BSP**	WH09	0.249±0.071 a	0.268±0.028 a	0.284±0.060 a	0.298±0.087 a	0.273±0.053 a	0.222±0.038 a	0.147-0.333
	WH10	0.250±0.029 c	0.311±0.018 ab	0.357±0.048 a	0.348±0.029 a	0.299±0.021 bc	0.266±0.029 bc	0.201-0.346
	HG10	0.276±0.047 b	0.294±0.056 b	0.312±0.010 b	0.383±0.042 a	0.330±0.031 ab	0.270±0.033 b	0.204-0.384
	GS11	0.230±0.035 a	0.254±0.020 a	0.271±0.043 a	0.269±0.048 a	0.249±0.034 a	0.229±0.035 a	0.155-0.317
**BN**	WH09	7.3±2.1 b	8.7±0.6 ab	8.8±1.1 ab	11.9±2.8 a	9.6±2.3 ab	7.0±1.0 b	5.3-10.1
	WH10	6.5±0.8 d	8.5±0.2 c	10.9±0.1 b	13.4±1.6 a	10.0±0.9 bc	8.5±1.3 c	5.3-12.5
	HG10	6.6±0.9 c	9.2±1.3 b	9.1±1.2 b	11.9±0.9 a	9.2±0.7 b	7.6±0.8 c	5.8-10.7
	GS11	5.4±0.5 c	6.0±0.7 bc	6.7±1.3 ab	7.2±1.0 a	6.3±0.7 bc	5.7±0.8 bc	4.2-8.2
**BTL (cm)**	WH09	500.5±170.3 a	541.2±49.6 a	546.8±137.9 a	477.3±179.2 a	488.9±163.3 a	388.8±75.5 a	237.3-639.4
	WH10	530.1±72.6 c	708.8±50.9 abc	877.5±99.4 a	714.7±194.9 ab	618.2±86.1 bc	595.1±73.0 bc	418.5-764.1
	HG10	516.6±121.0 b	719.2±145.0 a	712.3±70.2 a	713.8±81.8 a	615.2±58.0 ab	546.1±78.7 b	365.7-845.5
	GS11	398.0±54.1 b	418.7±50.5 ab	488.9±131.0 a	368.3±61.2 b	383.2±53.3 b	364.2±50.2 b	254.9-506.4
**BAL (cm)**	WH09	67.8±7.5 a	61.9±4.1 ab	61.8±7.4 ab	39.4±5.4 c	53.6±5.2 b	55.1±5.3 b	43.3-76.9
	WH10	81.1±6.8 a	83.0±7.6 a	80.7±9.1 a	52.8±8.3 c	66.5±4.0 b	71.1±9.4 ab	46.5-97.9
	HG10	78.2±10.0 a	78.1±6.6 a	78.6±3.4 a	60.1±6.2 b	69.1±5.6 ab	72.0±6.5 a	49.7-91.2
	GS11	74.2±5.2 a	69.7±6.3 ab	72.1±5.0 ab	51.3±6.0 d	62.7±3.9 c	64.1±6.0 bc	50.7-81.5
**MISN**	WH09	96.4±7.5 a	94.2±8.9 a	90.2±2.5 ab	70.4±4.1 c	83.4±4.7 b	82.6±8.9 b	63.2-103.1
	WH10	90.0±14.5 a	79.9±7.0 ab	82.7±3.4 ab	63.5±6.8 c	76.0±6.5 bc	79.9±7.9 ab	60.7-101.2
	HG10	84.8±8.8 a	83.1±4.8 ab	81.4±3.4 ab	62.4±5.5 c	73.6±5.8 b	79.1±8.7 ab	62.2-98.6
	GS11	66.9±25.4 a	58.7±2.7 a	57.1±3.1 a	53.6±6.2 a	60.3±12.0 a	55.2±7.7 a	36.1-74.7
**BSN**	WH09	360.0±195.4 a	431.4±79.9 a	415.0±144.1 a	289.3±52.6 a	324.6±121.3 a	254.9±60.3 a	115.4-477.8
	WH10	277.3±71.5 c	429.4±22.6 ab	445.1±83.1 a	298.7±130.0 bc	283.9±70.3 c	314.3±54.0 abc	193.1-488.0
	HG10	211.0±66.1 b	306.3±59.1 a	325.0±38.1 a	302.4±54.2 a	256.7±49.7 ab	253.7±51.0 ab	134.6-401.3
	GS11	201.5±52.3 b	284.8±46.5 ab	346.6±144.8 a	264.0±64.8 ab	232.8±50.7 b	215.9±56.8 b	105.8-653.2
**PSN**	WH09	456.7±198.4 a	525.6±85.5 a	505.2±146.6 a	359.7±56.5 a	408.2±124.5 a	337.7±64.0 a	178.6-571.5
	WH10	367.3±84.4 bc	509.3±15.6 ab	527.9±86.5 a	362.3±133.7 bc	359.9±75.7 c	394.2±58.4 abc	256.3-578.8
	HG10	295.7±72.3 b	389.4±63.9 a	406.4±41.5 a	364.8±54.7 a	330.3±53.4 ab	332.8±56.8 ab	196.9-481.9
	GS11	268.4±62.6 b	343.5±47.7 ab	403.7±146.5 a	317.7±63.8 ab	293.1±51.7 b	271.0±59.5 b	141.9-713.2
**MISNP**	WH09	0.276±0.074 a	0.210±0.042 a	0.207±0.065 a	0.222±0.048 a	0.249±0.044 a	0.272±0.042 a	0.171-0.451
	WH10	0.250±0.030 a	0.157±0.018 b	0.159±0.020 b	0.187±0.044 b	0.220±0.024 a	0.208±0.025 ab	0.139-0.276
	HG10	0.299±0.059 a	0.216±0.022 bc	0.201±0.012 bc	0.174±0.027 c	0.236±0.034 b	0.245±0.028 b	0.168-0.334
	GS11	0.250±0.062 a	0.173±0.020 b	0.152±0.042 b	0.175±0.040 b	0.213±0.042 a	0.214±0.035 ab	0.140-0.315
**MIY (g)**	WH09	5.62±0.95 a	4.83±0.53 ab	3.82±0.75 abc	2.32±2.01 c	3.97±0.91 abc	3.65±0.62 bc	2.06-5.31
	WH10	5.62±0.97 a	3.44±0.69 b	3.74±0.58 b	1.45±0.35 c	3.61±0.61 b	3.66±0.62 b	1.82-5.83
	HG10	6.08±0.77 a	4.42±0.32 b	4.18±0.28 b	1.62±0.31 c	3.85±0.45 b	4.28±0.66 b	2.17-6.90
	GS11	4.60±0.80 a	3.04±0.67 bc	3.82±0.60 b	0.99±0.26 d	2.80±0.47 c	2.70±0.65 c	1.07-4.13
**BY (g)**	WH09	16.70±6.96 ab	19.78±3.80 a	16.87±4.13 ab	7.85±5.81 c	12.27±5.76 abc	10.72±2.84 bc	4.94-24.48
	WH10	14.23±4.22 a	14.00±0.87 a	15.69±2.48 a	6.85±3.37 c	10.86±2.85 bc	11.99±2.13 ab	6.07-16.59
	HG10	12.24±5.08 a	13.95±2.98 a	14.56±1.48 a	7.55±2.54 b	9.90±2.53 ab	10.80±2.26 ab	4.79-18.73
	GS11	12.05±4.23 b	13.11±2.30 b	20.16±8.47 a	5.25±1.63 c	8.65±2.55 c	9.45±2.52 bc	2.64-18.54
**PY (g)**	WH09	22.32±7.49 ab	24.61±4.32 a	20.69±3.63 ab	10.17±7.81 c	16.24±6.66 abc	14.36±3.11 bc	7.36-29.79
	WH10	19.85±5.06 a	17.44±1.47 a	19.43±2.97 a	8.30±3.67 c	14.47±3.41 bc	15.64±2.53 ab	8.12-21.94
	HG10	18.32±5.74 a	18.37±3.29 a	18.74±1.22 a	9.18±2.79 b	13.75±2.92 ab	15.08±2.76 a	7.16-23.95
	GS11	16.65±4.88 b	16.15±2.41 b	23.98±9.00 a	6.24±1.82 d	11.44±2.92 cd	12.15±2.82 bc	4.54-21.98
**MIYP**	WH09	0.308±0.065 ab	0.240±0.042 ab	0.214±0.088 b	0.268±0.030 ab	0.288±0.026 ab	0.288±0.052 ab	0.160-0.483
	WH10	0.291±0.044 a	0.196±0.026 b	0.193±0.014 b	0.191±0.047 b	0.239±0.030 a	0.238±0.030 ab	0.147-0.320
	HG10	0.351±0.074 a	0.243±0.025 bc	0.224±0.028 bc	0.183±0.029 c	0.267±0.031 b	0.291±0.032 ab	0.214-0.380
	GS11	0.285±0.044 a	0.190±0.047 bc	0.168±0.042 c	0.163±0.028 c	0.224±0.033 ab	0.233±0.052 b	0.112-0.419
**SSN (10 silique)**	WH09	152.5±9.4 a	142.7±5.4 ab	164.8±29.4 a	86.9±27.7 c	119.7±13.5 b	147.5±29.8 ab	39.5-230.9
	WH10	173.8±19.4 b	204.7±10.6 a	197.2±17.1 ab	120.0±20.3 d	148.2±15.0 c	184.2±32.5 ab	86.6-262.8
	HG10	195.4±21.2 a	195.8±7.5 a	194.5±21.3 a	115.8±8.5 c	155.6±10.5 b	191.9±30.5 a	77.8-250.6
	GS11	228.8±18.2 a	205.4±23.6 ab	219.1±18.9 a	92.6±17.3 d	160.7±11.3 c	188.0±40.6 bc	72.2-294.7
**TSW (g)**	WH09	3.717±0.260 a	3.451±0.069 a	3.362±0.238 ab	3.301±0.525 ab	3.509±0.198 a	2.909±0.405 b	2.295-4.132
	WH10	3.652±0.573 a	2.678±0.245 b	2.658±0.115 b	2.525±0.373 b	3.099±0.366 b	2.445±0.356 b	1.858-3.996
	HG10	3.161±0.263 a	2.403±0.051 b	2.475±0.024 b	1.880±0.214 c	2.520±0.227 b	2.454±0.342 b	1.951-3.553
	GS11	3.646±0.199 a	2.860±0.186 b	3.100±0.228 b	2.544±0.228 c	3.095±0.178 b	2.890±0.350 b	2.297-4.033

[a] Average ± standard deviation.

[b] The lowercase letter indicates a significant difference at the 0.05 probability level based on Duncan-test among the different materials of the same trait.

Supplementary Table S2. ANOVA of trait performance and broad-sense heritabilities (h^2) of 20 plant architecture and yield related traits in the HJ-DH population.

Trait	Variation	*df*	MS	F value	P value	h^2 (%)	Trait	Variation	*df*	MS	F value	P value	h^2 (%)
**PH**	Genotype (G)	178	929.446	10.62	＜.0001	**83.3%**	**MISN**	Genotype (G)	178	537.708	13.19	＜.0001	**82.8%**
	Environment (E)	3	213480.942	2348.50	＜.0001			Environment (E)	3	91004.178	2231.76	＜.0001	
	G×E	567	155.344	1.77	＜.0001			G×E	567	92.654	2.27	＜.0001	
	Error	1479	87.546					Error	1478	40.777			
**MIL**	G	178	255.134	9.84	＜.0001	**68.9%**	**BSN**	G	178	18363.725	3.98	＜.0001	**67.1%**
	E	3	17432.330	672.34	＜.0001			E	3	918768.926	199.06	＜.0001	
	G×E	567	79.280	3.06	＜.0001			G×E	567	6047.587	1.31	＜.0001	
	Error	1478	25.928					Error	1478	4615.620			
**BH**	G	178	1005.654	13.35	＜.0001	**78.4%**	**PSN**	G	178	22126.841	4.42	＜.0001	**69.9%**
	E	3	60272.801	799.83	＜.0001			E	3	1408281.213	281.39	＜.0001	
	G×E	567	217.494	2.89	＜.0001			G×E	567	6651.767	1.33	＜.0001	
	Error	1477	75.357					Error	1478	5004.760			
**BS**	G	178	225.517	5.90	＜.0001	**72.9%**	**MISNP**	G	178	0.005	3.96	＜.0001	**60.0%**
	E	3	39458.050	1032.43	＜.0001			E	3	0.502	341.22	＜.0001	
	G×E	567	61.030	1.60	＜.0001			G×E	567	0.002	1.62	＜.0001	
	Error	1475	38.219					Error	1478	0.001			
**MILP**	G	178	0.018	14.05	＜.0001	**72.2%**	**MIY**	G	178	3.132	13.48	＜.0001	**82.2%**
	E	3	1.489	1130.74	＜.0001			E	3	238.522	1026.12	＜.0001	
	G×E	567	0.005	4.03	＜.0001			G×E	567	0.559	2.41	＜.0001	
	Error	1477	0.001					Error	1495	0.232			
**BSP**	G	178	0.008	5.92	＜.0001	**74.8%**	**BY**	G	178	31.438	3.59	＜.0001	**58.4%**

	E	3	0.342	259.11	<.0001			E	3	589.725	67.39	<.0001	
	G×E	567	0.002	1.49	<.0001			G×E	567	13.086	1.50	<.0001	
	Error	1475	0.001					Error	1490	8.751			
**BHP**	G	178	0.024	9.67	<.0001	**74.1%**	**PY**	G	178	45.005	4.30	<.0001	**64.6%**
	E	3	1.329	540.55	<.0001			E	3	1291.805	123.41	<.0001	
	G×E	567	0.006	2.50	<.0001			G×E	567	15.920	1.52	<.0001	
	Error	1477	0.002					Error	1489	10.468			
**BN**	G	178	6.959	9.16	<.0001	**74.8%**	**MIYP**	G	178	0.008	3.87	<.0001	**50.0%**
	E	3	771.946	1015.69	<.0001			E	3	0.557	253.50	<.0001	
	G×E	567	1.752	2.31	<.0001			G×E	567	0.004	1.93	<.0001	
	Error	1479	0.760					Error	1489	0.002			
**BTL**	G	178	30556.480	4.46	<.0001	**70.4%**	**SSN**	G	178	9539.147	26.53	<.0001	**87.0%**
	E	3	7361816.550	1073.38	<.0001			E	3	239600.156	666.25	<.0001	
	G×E	567	9051.460	1.32	<.0001			G×E	567	1244.492	3.46	<.0001	
	Error	1478	6858.570					Error	1476	359.623			
**BAL**	G	178	324.098	8.40	<.0001	**75.4%**	**TSW**	G	178	1.298	37.12	<.0001	**92.8%**
	E	3	34553.130	900.69	<.0001			E	3	38.257	1094.55	<.0001	
	G×E	567	79.691	2.08	<.0001			G×E	567	0.093	2.66	<.0001	
	Error	1478	38.363					Error	1499	0.035			

Supplementary Table S3. Correlation coefficients of 20 traits in winter-type (below diagonal) and spring-type (above diagonal) rapeseed area, respectively.

r	PH	BH	MIL	BS	BHP	MILP	BSP	BN	BTL	BAL	MISN	BSN	PSN	MISP	SSN	BY	PY	MIY	MIYP	TSW
**PH**		0.807**	-0.047	0.467**	0.655**	-0.613**	-0.015	0.522**	0.491**	-0.071	0.162*	0.301**	0.308**	-0.322**	-0.079	0.506**	0.489**	0.159*	-0.304**	0.051
**BH**	0.536**		-0.461**	0.263**	0.971**	-0.849**	-0.134	0.552**	0.210**	-0.534**	-0.088	0.124	0.108	-0.248**	0.014	0.411**	0.381**	0.059	-0.272**	-0.016
**MIL**	0.362**	-0.461**		-0.409**	-0.562**	0.769**	-0.452**	-0.601**	-0.148*	0.710**	0.570**	0.042	0.114	0.311**	-0.225**	-0.235**	-0.151*	0.255**	0.383**	0.015
**BS**	0.727**	-0.014	0.381**		0.152*	-0.604**	0.872**	0.770**	0.763**	-0.055	-0.288**	0.282**	0.232**	-0.569**	0.141*	0.508**	0.425**	-0.129	-0.567**	0.068
**BHP**	0.057	0.870**	-0.751**	-0.434**		-0.852**	-0.180*	0.506**	0.081	-0.656**	-0.151*	0.043	0.021	-0.177*	0.047	0.329**	0.300**	0.026	-0.217**	-0.051
**MILP**	-0.369**	-0.848**	0.726**	-0.150**	-0.795**		-0.358**	-0.791**	-0.389**	0.642**	0.360**	-0.126	-0.074	0.420**	-0.146*	-0.474**	-0.399**	0.105	0.472**	0.006
**BSP**	0.374**	-0.358**	0.317**	0.905**	-0.640**	0.043		0.584**	0.594**	-0.024	-0.424**	0.158*	0.096	-0.483**	0.194**	0.306**	0.216**	-0.247**	-0.504**	0.083
**BN**	0.631**	0.355**	-0.074	0.724**	0.049	-0.525**	0.586**		0.778**	-0.389**	-0.314**	0.371**	0.315**	-0.686**	0.140*	0.635**	0.539**	-0.123	-0.642**	-0.012
**BTL**	0.713**	-0.049	0.540**	0.871**	-0.469**	0.021	0.746**	0.751**		0.269**	-0.141*	0.518**	0.477**	-0.751**	0.026	0.631**	0.537**	-0.118	-0.675**	0.152*
**BAL**	0.420**	-0.445**	0.896**	0.563**	-0.770**	0.589**	0.523**	0.091*	0.716**		0.269**	0.189**	0.215**	-0.068	-0.182**	-0.048	-0.038	0.018	-0.016	0.230**
**MISN**	0.054	0.210**	0.010	-0.214**	0.242**	-0.054	-0.331**	-0.028	-0.090*	-0.151**		0.286**	0.402**	0.321**	-0.358**	-0.066	0.013	0.311**	0.323**	-0.121
**BSN**	0.406**	0.165**	0.117**	0.400**	-0.041	-0.175**	0.290**	0.597**	0.589**	0.265**	0.381**		0.992**	-0.676**	-0.345**	0.437**	0.369**	-0.091	-0.450**	0.110
**PSN**	0.388**	0.181**	0.112*	0.348**	-0.008	-0.170**	0.229**	0.558**	0.543**	0.230**	0.489**	0.993**		-0.605**	-0.375**	0.409**	0.355**	-0.047	-0.389**	0.090
**MISP**	-0.545**	-0.094*	-0.245**	-0.637**	0.221**	0.139**	-0.540**	-0.681**	-0.768**	-0.482**	0.076	-0.826**	-0.769**		0.139	-0.620**	-0.493**	0.266**	0.798**	-0.188**
**SSN**	0.560**	0.309**	0.137**	0.456**	0.030	-0.264**	0.285**	0.326**	0.344**	0.201**	-0.303**	-0.109*	-0.144**	-0.187**		0.269**	0.348**	0.462**	0.169*	-0.480**
**BY**	0.381**	0.185**	0.057	0.383**	-0.003	-0.222**	0.286**	0.504**	0.468**	0.189**	0.181**	0.741**	0.722**	-0.671**	0.183**		0.976**	0.350**	-0.552**	0.022
**PY**	0.430**	0.223**	0.098*	0.378**	0.019	-0.223**	0.251**	0.453**	0.434**	0.186**	0.221**	0.643**	0.634**	-0.581**	0.297**	0.974**		0.545**	-0.369**	-0.073
**MIY**	0.379**	0.249**	0.188**	0.161**	0.094*	-0.116**	-0.008	0.028	0.078	0.072	0.250**	-0.054	-0.020	0.058	0.560**	0.366**	0.566**		0.538**	-0.398**
**MIYP**	-0.293**	-0.015	-0.105*	-0.421**	0.171**	0.091*	-0.396**	-0.550**	-0.580**	-0.333**	0.007	-0.786**	-0.740**	0.857**	0.083	-0.692**	-0.542**	0.294**		-0.409**
**TSW**	-0.485**	-0.349**	-0.069	-0.327**	-0.128**	0.280**	-0.144**	-0.295**	-0.288**	-0.132**	-0.065	-0.090*	-0.091*	0.252**	-0.634**	-0.006	-0.066	-0.250**	0.009	

* and ** indicate a significant difference at the 0.05 probability level and 0.01 probability level, respectively.

Supplementary Table S4. Detailed information of identified QTLs.

ID	Env.	Trait	LG	Peak	LOD	A	R^2	Interval
1	WH09	PH	BnA01	0.0	5.74	-2.697	8.3%	0-2.3
2	WH09	SSN	BnA01	15.8	6.11	11.525	10.0%	14.1-16.8
3	WH09	TSW	BnA01	23.7	6.36	-0.136	7.5%	23.1-26.1
4	WH09	MIL	BnA01	25.8	9.39	-2.585	18.6%	25.6-26.9
5	WH09	BSP	BnA01	124.7	3.69	0.011	6.5%	116.8-127.7
6	WH09	TSW	BnA01	125.7	4.04	-0.128	6.1%	121-127.7
7	WH09	BS	BnA02	9.6	3.54	-1.641	5.5%	3.9-14.4
8	WH09	BTL	BnA02	11.6	4.77	-20.814	5.7%	5.8-13.9
9	WH09	BN	BnA02	11.7	6.47	-0.296	6.7%	7.6-13.9
10	WH09	BSN	BnA02	11.7	4.90	-19.697	7.2%	8.1-14.2
11	WH09	MISNP	BnA02	11.7	5.57	0.013	7.1%	7.3-13.9
12	WH09	MIYP	BnA02	11.7	3.55	0.013	5.0%	8.6-14.6
13	WH09	SSN	BnA02	12.7	5.58	10.478	9.4%	7.8-15.4
14	WH09	PSN	BnA02	16.8	5.52	-24.574	8.4%	15.9-17.4
15	WH09	BY	BnA02	21.7	5.52	-1.062	8.8%	21.5-22
16	WH09	PY	BnA02	21.7	12.34	-1.584	21.9%	21-22.3
17	WH09	MILP	BnA02	26.5	12.95	0.017	18.3%	25.6-26.8
18	WH09	BHP	BnA02	27.4	4.08	-0.014	5.6%	24.9-28.7
19	WH09	BSP	BnA02	56.7	8.96	-0.016	13.1%	56.3-57.3
20	WH09	BHP	BnA02	71.7	6.41	0.018	9.0%	71.5-72.7
21	WH09	PY	BnA03	20.1	3.56	0.946	5.6%	18.7-25.3
22	WH09	TSW	BnA03	43.4	9.34	0.162	11.9%	42-45.4
23	WH09	TSW	BnA03	124.7	3.69	0.130	6.6%	123.3-130.8
24	WH09	BH	BnA04	40.5	4.62	-2.530	7.6%	38.7-42.6
25	WH09	MIYP	BnA05	59.5	6.31	0.019	10.1%	58.2-60.8
26	WH09	MILP	BnA05	62.5	3.44	-0.009	4.3%	60.5-62.6
27	WH09	MIY	BnA06	0.3	8.78	0.260	14.1%	0-1.3
28	WH09	SSN	BnA06	58.7	7.21	11.800	12.0%	57-59.8
29	WH09	PH	BnA07	81.5	6.20	-2.839	9.0%	80.9-83.4
30	WH09	BS	BnA07	93.1	7.96	-2.467	11.6%	92.5-94.2
31	WH09	TSW	BnA07	112.0	3.89	-0.108	4.6%	111.9-115
32	WH09	TSW	BnA07	121.9	5.28	-0.121	6.2%	121.5-122.2

33	WH09	PH	BnA08	2.0	6.63	-2.969	10.6%	0-5
34	WH09	MISNP	BnA08	15.1	9.80	0.020	13.2%	14.4-16.3
35	WH09	BY	BnA08	15.1	4.33	-0.997	6.8%	13.5-16.4
36	WH09	MIYP	BnA08	16.0	8.98	0.025	14.6%	14.9-16.9
37	WH09	BTL	BnA08	29.9	8.94	-31.692	11.3%	29.4-30.5
38	WH09	BSN	BnA08	30.2	9.99	-32.715	15.8%	29.3-30.5
39	WH09	PSN	BnA08	30.2	12.41	-40.484	23.7%	29.7-31
40	WH09	BN	BnA08	34.3	7.74	-0.381	8.6%	32.9-36.5
41	WH09	BSP	BnA08	65.2	3.45	-0.010	5.2%	59-68.5
42	WH09	BSP	BnA09	26.7	11.16	-0.018	19.0%	23.8-27.7
43	WH09	MISN	BnA09	47.0	5.69	2.499	6.1%	43.3-50.8
44	WH09	MIY	BnA09	76.8	5.35	0.195	8.2%	75.3-79.4
45	WH09	MIL	BnA09	78.8	4.25	1.526	7.3%	78.5-81.6
46	WH09	MILP	BnA09	81.8	8.94	0.014	13.1%	79.5-83.4
47	WH09	BN	BnC01	3.7	4.98	-0.281	4.9%	2.8-5.6
48	WH09	BH	BnC02	9.3	3.89	4.042	12.0%	5.7-14
49	WH09	BN	BnC02	35.1	5.88	0.298	6.0%	34.5-36.1
50	WH09	BSP	BnC02	63.0	3.73	0.013	7.5%	55.3-70.9
51	WH09	BAL	BnC03	38.2	5.24	-1.727	8.0%	27.8-43.5
52	WH09	BTL	BnC03	93.1	6.31	-24.172	7.7%	92.5-98.8
53	WH09	BS	BnC03	102.6	5.16	-1.870	7.0%	100.3-102.9
54	WH09	BH	BnC04	11.5	6.42	-3.187	10.2%	10.7-12.1
55	WH09	PH	BnC04	65.4	8.15	3.396	12.1%	64.2-67.4
56	WH09	BY	BnC04	89.7	5.72	1.109	9.2%	89.3-90.8
57	WH09	PY	BnC04	89.7	7.62	1.398	12.7%	89.4-90.8
58	WH09	MIY	BnC04	102.4	9.64	0.307	19.9%	98.2-104.4
59	WH09	BH	BnC04	105.1	5.93	2.664	8.2%	104.7-108.8
60	WH09	BHP	BnC04	116.1	4.80	0.015	7.5%	115.1-123
61	WH09	BSP	BnC04	140.7	4.39	-0.011	6.0%	139.2-141.6
62	WH09	BAL	BnC05	56.5	4.02	-1.558	6.5%	48.0-60.1
63	WH09	BY	BnC05	59.7	5.14	-0.993	9.2%	58.5-61.8
64	WH09	PY	BnC05	59.7	5.95	-1.245	11.0%	58.5-61.6
65	WH09	TSW	BnC05	101.5	4.02	0.096	4.2%	96.7-102.8
66	WH09	MISN	BnC06	2.9	5.02	2.546	5.3%	1.9-3.0

67	WH09	MIL	BnC06	14.8	7.30	2.093	14.2%	11.4-19.0
68	WH09	TSW	BnC06	39.5	15.08	0.166	13.6%	33.2-39.8
69	WH09	BAL	BnC06	45.5	10.31	2.671	16.9%	43.7-46.9
70	WH09	BH	BnC06	45.5	14.49	-4.487	25.7%	44.4-46.2
71	WH09	BHP	BnC06	45.5	9.56	-0.022	14.0%	41.6-46.3
72	WH09	MILP	BnC06	49.1	14.65	0.018	20.8%	48.8-49.1
73	WH09	TSW	BnC07	14.3	3.69	-0.109	4.3%	13.4-17.2
74	WH09	BTL	BnC07	19.1	3.47	-19.876	4.3%	16.6-20.9
75	WH09	TSW	BnC07	63.4	5.04	0.121	6.6%	60.2-67.7
76	WH09	BN	BnC08	59.9	5.03	-0.269	5.1%	58.5-60.6
77	WH09	BSN	BnC08	59.9	4.93	-21.122	7.3%	56.1-63.8
78	WH09	PSN	BnC08	59.9	6.00	-28.271	10.5%	56.1-62.8
79	WH09	MIYP	BnC08	61.8	9.46	0.025	16.8%	60.5-63.8
80	WH09	MISNP	BnC08	72.9	7.58	0.018	11.6%	69.9-75
81	WH09	MISN	BnC08	73.9	5.39	-2.936	7.0%	71.6-76.7
82	WH09	BSN	BnC08	127.7	4.24	-18.707	6.2%	126.8-129.2
83	WH09	PSN	BnC08	127.7	3.72	-19.406	6.3%	123.6-129.7
84	WH09	MISN	BnC08	128.7	14.66	-5.028	18.7%	127.7-129.9
85	WH09	MIYP	BnC09	97.9	7.18	0.039	12.0%	96.3-99.2
86	WH10	MIY	BnA01	15.8	9.52	0.268	13.6%	15.5-17
87	WH10	TSW	BnA01	19.6	8.77	-0.144	10.8%	19.2-20.7
88	WH10	SSN	BnA01	26.8	11.53	18.219	25.1%	25.2-27.8
89	WH10	MILP	BnA01	29.3	5.68	-0.020	6.3%	28.6-30.4
90	WH10	MIL	BnA01	49.5	4.11	-1.962	5.6%	49.5-51.8
91	WH10	BAL	BnA01	57.1	4.18	-2.131	3.7%	53.7-60.4
92	WH10	BH	BnA02	18.6	6.55	-5.105	6.7%	18.2-18.9
93	WH10	BHP	BnA02	18.6	8.00	-0.025	8.3%	18.2-18.9
94	WH10	MISN	BnA02	19.8	6.51	-3.081	11.5%	17.3-22
95	WH10	BAL	BnA02	22.5	18.95	4.989	20.9%	22-27.4
96	WH10	BN	BnA02	22.8	6.03	-0.399	6.5%	22.4-25.4
97	WH10	MIL	BnA02	22.8	5.21	2.209	6.8%	22.4-24.2
98	WH10	BH	BnA02	53.9	8.32	5.790	8.7%	52.8-55.2
99	WH10	BHP	BnA02	53.9	7.88	0.025	8.2%	52.6-54.9
100	WH10	BAL	BnA02	63.6	9.61	-3.562	9.1%	63.3-66.1

101	WH10	PY	BnA03	13.5	3.77	0.715	5.4%	12-16.3
102	WH10	TSW	BnA03	31.4	4.54	0.147	11.3%	26.6-35.4
103	WH10	SSN	BnA03	70.3	5.55	-11.413	8.9%	68.2-72.3
104	WH10	MIL	BnA03	71.4	4.37	1.999	5.0%	70.3-72.9
105	WH10	BSP	BnA03	153.8	7.67	-0.019	15.1%	153.4-156.4
106	WH10	BTL	BnA03	163.9	5.85	-27.011	9.9%	163-165
107	WH10	MIY	BnA03	176.8	4.88	0.178	6.6%	174.9-178.5
108	WH10	BSP	BnA03	183.9	4.32	0.011	8.6%	182-186.2
109	WH10	BH	BnA04	41.9	7.34	-5.077	8.4%	41.2-44
110	WH10	BHP	BnA04	42.9	9.88	-0.027	11.6%	41.1-44.3
111	WH10	PY	BnA04	49.5	5.57	-0.875	9.7%	47.2-50.8
112	WH10	BY	BnA04	55.0	3.50	-0.592	5.3%	50.8-57.9
113	WH10	MIY	BnA04	57.2	7.59	-0.218	10.7%	56.4-62.8
114	WH10	PY	BnA05	20.4	9.38	1.082	14.4%	19.7-22.5
115	WH10	MIL	BnA05	45.7	4.09	-1.939	4.7%	44.5-47.6
116	WH10	MIL	BnA06	8.2	3.28	1.638	3.8%	7.3-9.9
117	WH10	PSN	BnA06	63.9	4.27	-16.840	6.7%	61.3-65.2
118	WH10	BSN	BnA06	64.2	4.01	-15.248	6.7%	61.8-65.2
119	WH10	MIL	BnA07	40.9	4.92	-2.278	5.6%	39.6-43.1
120	WH10	MIYP	BnA07	98.6	4.89	0.011	9.6%	97.9-98.9
121	WH10	BAL	BnA08	1.0	8.03	-3.271	8.4%	0-3.4
122	WH10	BSN	BnA08	15.1	7.95	-24.964	13.9%	13.7-17.3
123	WH10	PSN	BnA08	16.6	11.54	-38.426	19.7%	16-17.6
124	WH10	BS	BnA08	37.3	4.46	-2.060	10.2%	33.3-41.9
125	WH10	BTL	BnA08	37.8	9.23	-33.875	15.9%	36.3-40.6
126	WH10	PH	BnA08	43.3	5.23	-4.414	7.6%	43.1-46.5
127	WH10	PY	BnA09	51.7	3.91	0.738	6.5%	48.6-52.5
128	WH10	SSN	BnA09	52.5	4.05	-9.538	7.1%	49-58.5
129	WH10	TSW	BnA09	53.1	6.26	0.113	7.5%	52.8-55.5
130	WH10	MIL	BnA09	59.4	6.10	2.387	8.0%	56.7-62.4
131	WH10	MISN	BnA09	62.4	7.75	2.962	11.5%	61.1-63.1
132	WH10	MILP	BnA09	65.0	5.95	0.021	6.6%	64.4-67.3
133	WH10	MIL	BnA10	39.1	4.92	-2.130	5.6%	34.1-42.4
134	WH10	BS	BnA10	45.8	3.84	2.069	7.4%	43.7-47.1

135	WH10	SSN	BnA10	45.8	6.96	13.174	10.9%	45.2-56.3
136	WH10	PH	BnA10	46.8	3.57	4.109	5.2%	45.2-48.2
137	WH10	MILP	BnA10	47.2	4.01	-0.018	4.4%	46.2-48.5
138	WH10	BY	BnA10	49.9	5.08	0.798	8.8%	47.8-53.1
139	WH10	PY	BnA10	49.9	4.39	0.918	8.6%	47.6-53.9
140	WH10	MISNP	BnA10	56.2	3.78	-0.009	6.9%	55.2-57
141	WH10	TSW	BnC01	0.0	8.58	-0.135	10.6%	0-1.7
142	WH10	MISN	BnC01	53.7	4.72	2.320	7.2%	51.9-55.5
143	WH10	PY	BnC02	2.0	4.33	1.066	5.5%	1-7.3
144	WH10	MIY	BnC02	7.3	6.12	0.328	12.2%	2.3-11.8
145	WH10	MISN	BnC02	8.3	4.40	4.222	11.3%	4.7-12.5
146	WH10	BN	BnC02	42.8	9.01	0.478	10.1%	42.2-43.6
147	WH10	MISN	BnC02	55.5	3.51	2.153	5.0%	53.1-62.4
148	WH10	PY	BnC02	97.2	3.23	-0.586	4.6%	97.2-99.1
149	WH10	MIYP	BnC03	77.8	3.54	0.013	7.5%	74.5-79.6
150	WH10	MIY	BnC03	85.1	3.57	0.148	4.8%	81.5-88.6
151	WH10	SSN	BnC03	102.9	4.57	9.856	8.2%	101.7-103.4
152	WH10	BH	BnC04	18.3	5.79	-4.998	7.1%	18.2-19.3
153	WH10	PH	BnC04	18.3	4.87	-4.692	7.9%	17.5-19.3
154	WH10	BHP	BnC04	19.3	4.29	-0.019	5.0%	18.2-19.9
155	WH10	BY	BnC04	59.3	4.97	0.789	8.3%	58-60.3
156	WH10	MIY	BnC04	69.4	3.65	0.210	5.6%	66.5-74.6
157	WH10	MISNP	BnC04	106.1	6.31	-0.009	12.5%	102.7-110
158	WH10	PY	BnC04	109.9	3.68	0.670	6.1%	105.6-118.9
159	WH10	BHP	BnC04	120.0	3.47	0.015	3.5%	113.3-123.6
160	WH10	MILP	BnC04	122.0	3.95	-0.018	4.7%	119-124.7
161	WH10	MILP	BnC04	147.3	3.77	0.017	4.1%	145.9-148.8
162	WH10	MISNP	BnC05	95.5	3.81	0.007	6.9%	83.9-98.7
163	WH10	BN	BnC05	116.2	4.16	-0.319	4.4%	113.3-117.4
164	WH10	BS	BnC06	39.5	6.15	-2.262	13.6%	39.2-40.4
165	WH10	BN	BnC06	39.8	27.91	-0.897	40.7%	39.5-40.2
166	WH10	MIL	BnC06	44.5	27.42	6.273	51.8%	42.5-45.5
167	WH10	PH	BnC06	45.5	10.48	-6.558	16.0%	43.9-47.2
168	WH10	BAL	BnC06	54.8	24.08	5.474	28.5%	53.3-55.4

169	WH10	BH	BnC06	54.8	22.78	-9.588	29.0%	53.0-55.8
170	WH10	BHP	BnC06	54.8	22.34	-0.042	28.3%	52.9-55.2
171	WH10	MILP	BnC06	54.8	20.76	0.045	28.1%	53.8-55.1
172	WH10	TSW	BnC06	54.8	6.12	0.129	7.3%	53.3-56.6
173	WH10	MISN	BnC07	11.3	3.51	2.286	6.0%	8.8-16.6
174	WH10	BTL	BnC07	68.6	4.88	-23.018	7.5%	66.6-69.7
175	WH10	BSN	BnC08	113.0	4.24	-17.393	8.2%	107-116
176	WH10	PSN	BnC08	114.0	7.29	-23.909	13.0%	110.2-115.9
177	WH10	MISN	BnC08	117.9	19.08	-5.622	34.2%	115.9-120.3
178	WH10	SSN	BnC08	118.4	3.48	8.791	6.4%	115.9-122.1
179	WH10	BY	BnC08	137.6	7.10	0.801	11.4%	136.6-138.5
180	WH10	PY	BnC08	137.6	7.28	0.936	10.8%	136.4-138.7
181	WH10	PH	BnC09	88.2	4.54	5.068	7.5%	84.6-91
182	WH10	BAL	BnC09	159.5	5.11	3.913	4.7%	157.7-161.8
183	HG10	PH	BnA01	5.3	4.62	-2.775	5.8%	4.3-7.5
184	HG10	MIL	BnA01	9.1	7.06	-1.954	11.0%	7.9-11.1
185	HG10	MIYP	BnA01	9.1	4.24	0.010	7.8%	7.9-10.1
186	HG10	BAL	BnA01	9.1	3.96	-2.009	7.2%	5.7-12.3
187	HG10	SSN	BnA01	15.8	13.61	16.821	21.6%	14.1-17.3
188	HG10	MIY	BnA01	28.7	7.13	0.266	11.4%	28.4-29.3
189	HG10	PY	BnA01	29.6	4.36	0.977	8.1%	29.3-30.6
190	HG10	BSN	BnA01	121.7	3.79	19.583	12.1%	114.8-128.1
191	HG10	SSN	BnA02	14.7	6.84	11.514	10.4%	11.7-15.8
192	HG10	BHP	BnA02	24.3	6.05	-0.016	7.4%	22.5-25.6
193	HG10	BSN	BnA02	31.4	3.41	-16.264	6.9%	29.8-32.8
194	HG10	PH	BnA02	57.2	6.72	3.543	8.8%	55.6-58.4
195	HG10	MISN	BnA02	96.8	12.73	3.678	15.3%	95.2-98.5
196	HG10	PSN	BnA02	97.8	3.86	16.754	6.5%	94.5-98.8
197	HG10	TSW	BnA03	43.7	13.38	0.151	15.0%	42.6-47.2
198	HG10	BHP	BnA03	71.4	5.63	-0.017	6.8%	70.5-72.5
199	HG10	BY	BnA03	92.1	4.76	-0.839	9.1%	89.3-99.7
200	HG10	BSN	BnA03	92.1	3.21	-16.053	6.6%	89-101.2
201	HG10	MISNP	BnA03	92.1	3.28	0.009	6.2%	89-100.1
202	HG10	BAL	BnA03	133.7	4.43	2.993	13.4%	128.1-139.4

203	HG10	BN	BnA03	163.0	4.00	-0.237	5.8%	161.9-163.7
204	HG10	MISN	BnA03	175.0	6.19	2.583	6.9%	173.3-176.4
205	HG10	MISN	BnA04	22.2	3.51	2.372	6.0%	14.5-31.8
206	HG10	MILP	BnA04	40.5	3.95	0.009	4.8%	38.9-44.5
207	HG10	SSN	BnA06	0.3	4.45	8.944	5.6%	0-1.9
208	HG10	BS	BnA06	34.7	7.97	2.254	11.3%	34.1-35.9
209	HG10	PH	BnA07	8.3	6.76	4.231	11.8%	6.8-12.1
210	HG10	BAL	BnA07	85.1	3.87	-1.821	7.0%	82.4-87.9
211	HG10	TSW	BnA07	87.1	7.64	-0.111	8.2%	86.7-88.9
212	HG10	TSW	BnA07	96.2	8.95	-0.126	9.4%	95.3-96.7
213	HG10	BTL	BnA07	111.7	8.86	-36.687	15.3%	110.8-113.4
214	HG10	BSN	BnA08	16.6	6.96	-24.149	13.4%	15.1-17.6
215	HG10	PH	BnA08	30.2	14.05	-5.324	19.2%	29.9-33.4
216	HG10	PSN	BnA08	30.2	6.35	-22.355	10.5%	26.3-33.3
217	HG10	BH	BnA08	32.3	4.61	-3.242	6.2%	31.7-39
218	HG10	BN	BnA08	45.4	8.06	-0.324	12.8%	44.3-47.8
219	HG10	BS	BnA09	19.7	6.37	-2.025	8.9%	15.7-22.6
220	HG10	BSP	BnA09	19.7	16.95	-0.020	28.1%	18.5-23
221	HG10	TSW	BnA09	53.1	6.04	0.102	6.1%	52.6-54.6
222	HG10	SSN	BnA09	56.1	4.72	-10.041	7.5%	53.1-59.6
223	HG10	MIL	BnA09	59.4	6.81	1.899	10.7%	56.7-61.6
224	HG10	MISN	BnA09	60.4	6.13	2.537	7.0%	57.6-63.4
225	HG10	MILP	BnA09	62.4	3.43	0.009	3.6%	58-66.6
226	HG10	BN	BnA10	43.1	6.29	0.304	9.4%	42.3-44
227	HG10	MIY	BnA10	45.8	7.10	0.283	11.3%	45.2-47.1
228	HG10	PH	BnA10	46.8	8.09	4.275	10.5%	46.6-48.2
229	HG10	BH	BnA10	47.2	6.72	4.425	9.4%	46.4-48.7
230	HG10	MILP	BnA10	48.2	10.35	-0.018	13.3%	46.6-51
231	HG10	BHP	BnA10	48.9	4.77	0.017	5.7%	46.7-50.5
232	HG10	BY	BnA10	62.3	4.02	0.785	7.9%	57.8-63.3
233	HG10	SSN	BnC01	0.0	5.57	9.688	6.9%	0-2.7
234	HG10	BN	BnC01	4.6	8.10	-0.341	12.3%	3.7-5.6
235	HG10	MIYP	BnC01	71.4	5.22	-0.010	9.6%	68.7-78.3
236	HG10	MISN	BnC02	14.3	4.84	3.350	7.3%	9.5-16.9

237	HG10	BSP	BnC02	51.3	3.51	-0.009	4.9%	50-53.4
238	HG10	BTL	BnC03	94.0	4.14	-22.414	6.9%	93-96
239	HG10	BHP	BnC03	95.0	6.74	0.018	9.0%	93.5-99
240	HG10	BH	BnC03	103.4	5.59	3.446	7.7%	102.6-103.4
241	HG10	BS	BnC04	2.9	5.45	1.861	7.5%	1-5.6
242	HG10	MISNP	BnC04	18.8	3.05	0.008	6.3%	14.6-22.9
243	HG10	MIYP	BnC04	19.3	5.42	0.013	11.6%	17.3-21.1
244	HG10	BSN	BnC04	19.9	3.59	-16.737	8.0%	17.1-22.1
245	HG10	MIY	BnC04	54.6	6.91	0.271	11.0%	54.1-55.9
246	HG10	BY	BnC04	55.6	5.33	0.980	10.8%	53.1-56
247	HG10	PY	BnC04	55.6	8.14	1.502	16.6%	55-56
248	HG10	BSN	BnC05	9.8	4.47	-17.785	8.9%	8.6-11.1
249	HG10	PSN	BnC05	9.8	4.41	-19.138	7.2%	9.2-10.7
250	HG10	MILP	BnC05	98.5	3.81	0.011	4.9%	95.6-102.3
251	HG10	MIY	BnC05	113.5	5.69	0.243	9.4%	111.2-118.0
252	HG10	SSN	BnC06	5.5	4.50	-8.875	6.3%	4.1-8.8
253	HG10	TSW	BnC06	31.3	12.93	0.158	15.4%	29.8-32.2
254	HG10	MIL	BnC06	40.7	8.56	2.298	13.7%	39.8-43.8
255	HG10	MILP	BnC06	43.5	16.50	0.022	23.5%	42.4-44.1
256	HG10	BH	BnC06	45.5	8.07	-4.618	11.2%	43.4-47.3
257	HG10	BHP	BnC06	45.5	6.96	-0.019	8.2%	43.4-47.3
258	HG10	BAL	BnC06	54.8	6.57	2.566	13.7%	52.8-57.2
259	HG10	BH	BnC07	17.6	3.87	3.426	5.5%	15.4-20.5
260	HG10	MIY	BnC07	26.2	4.08	0.214	6.4%	25.1-28.5
261	HG10	TSW	BnC07	75.8	3.78	0.071	3.4%	75.2-76.7
262	HG10	BHP	BnC08	76.0	3.54	-0.012	3.8%	70.6-76.8
263	HG10	BH	BnC08	77.7	4.55	-3.210	5.5%	76.8-78.9
264	HG10	SSN	BnC08	126.4	7.55	12.459	11.2%	125-127.5
265	HG10	MISN	BnC08	132.0	13.95	-3.939	17.5%	130.4-133
266	HG10	BSN	BnC08	133.0	6.74	-19.515	12.1%	129.9-134
267	HG10	PSN	BnC08	133.0	9.07	-24.876	15.7%	131.1-134
268	HG10	MILP	BnC08	135.0	3.77	-0.009	4.4%	133.7-135.3
269	HG10	BSP	BnC08	143.7	7.78	0.013	11.3%	141.6-144.2
270	HG10	BSP	BnC09	18.7	5.36	0.011	7.9%	16.3-20.3

271	HG10	BN	BnC09	125.0	3.91	-0.856	8.2%	124-126.2
272	HG10	BS	BnC09	125.0	6.38	-8.982	11.4%	124-126.2
273	HG10	BTL	BnC09	126.6	3.65	-54.659	5.9%	125.2-128.1
274	HG10	BAL	BnC09	130.2	3.63	-4.044	6.6%	128.4-131.3
275	GS11	BAL	BnA01	7.6	5.18	-1.995	8.2%	6.4-9.1
276	GS11	BH	BnA01	31.9	7.79	2.903	6.5%	31.5-34.2
277	GS11	BHP	BnA01	31.9	5.88	0.016	5.2%	31.4-37.2
278	GS11	PSN	BnA02	15.3	3.13	-17.866	6.3%	12.1-16.7
279	GS11	BH	BnA02	18.6	26.44	-6.341	33.4%	18.3-19.3
280	GS11	BHP	BnA02	18.6	30.24	-0.040	37.7%	18.3-18.9
281	GS11	MILP	BnA02	18.6	25.49	0.035	22.5%	18.3-18.7
282	GS11	MIL	BnA02	22.0	8.10	2.599	9.6%	19.4-23.6
283	GS11	BAL	BnA02	22.8	9.19	2.613	15.1%	22-24.3
284	GS11	TSW	BnA02	22.8	4.76	0.086	4.7%	22.1-25.3
285	GS11	BY	BnA02	23.1	11.81	-1.367	20.8%	22.5-24.8
286	GS11	PY	BnA02	24.1	8.17	-1.265	14.6%	22.8-25.3
287	GS11	PH	BnA02	24.3	17.03	-5.529	23.8%	24.1-25.4
288	GS11	BN	BnA02	25.3	21.89	-0.488	33.6%	24.3-26.3
289	GS11	SSN	BnA02	66.6	7.75	-17.267	15.2%	65.6-67.8
290	GS11	MISN	BnA02	96.8	6.06	2.510	8.4%	95.6-97.8
291	GS11	BHP	BnA03	0.0	3.30	0.012	2.8%	0-4.1
292	GS11	PH	BnA03	52.4	11.07	-4.483	15.7%	49.8-55.2
293	GS11	PH	BnA03	142.0	3.91	3.372	4.6%	141.4-143
294	GS11	MISN	BnA03	147.6	3.98	2.717	5.8%	146.2-150.9
295	GS11	SSN	BnA04	3.2	6.25	14.829	12.5%	1.5-4.5
296	GS11	MIYP	BnA04	56.4	17.01	-0.035	25.9%	55.9-62.5
297	GS11	MIY	BnA04	57.2	6.49	-0.223	8.5%	56.4-58.5
298	GS11	MIYP	BnA05	45.7	4.11	0.014	5.2%	44.8-48.9
299	GS11	MIL	BnA06	24.9	6.30	2.194	7.8%	20.8-28.1
300	GS11	SSN	BnA06	57.0	7.18	16.615	14.1%	55.8-61
301	GS11	TSW	BnA07	68.5	5.23	-0.141	10.0%	63.3-73.3
302	GS11	BN	BnA07	101.8	4.35	-0.216	4.7%	100.7-104.9
303	GS11	MIY	BnA07	101.8	4.39	0.191	5.6%	100.7-102.9
304	GS11	PH	BnA08	16.6	3.86	-2.938	4.1%	16.2-19.8

305	GS11	BHP	BnA08	27.6	4.77	-0.015	4.1%	24.3-28.7
306	GS11	PH	BnA08	27.6	3.54	-2.734	3.7%	24.6-28.7
307	GS11	BTL	BnA08	38.3	5.06	-16.439	9.2%	35.6-40.7
308	GS11	BN	BnA08	39.6	7.25	-0.258	7.7%	34.9-41.9
309	GS11	MILP	BnA08	40.6	4.13	0.013	2.9%	35.9-41.9
310	GS11	BH	BnA08	41.9	7.20	-2.798	6.2%	41.3-43
311	GS11	SSN	BnA08	46.7	4.79	-14.132	9.7%	45.4-47.7
312	GS11	MISN	BnA08	52.5	5.57	2.398	7.6%	51.3-54.3
313	GS11	MIL	BnA09	40.7	7.04	2.394	8.8%	36.2-46
314	GS11	SSN	BnA09	51.7	4.61	-13.292	9.4%	47.8-53.1
315	GS11	BAL	BnA09	89.2	3.91	1.685	6.0%	88-90.8
316	GS11	MILP	BnA10	50.9	6.05	-0.017	4.6%	48.9-54.5
317	GS11	BS	BnA10	62.3	3.71	1.379	5.6%	59.3-63.3
318	GS11	MILP	BnC01	1.7	7.39	0.017	5.1%	0.4-7.5
319	GS11	BN	BnC01	3.7	7.17	-0.263	7.4%	2.8-4.9
320	GS11	TSW	BnC01	56.3	6.96	-0.107	7.1%	54.3-57.4
321	GS11	BN	BnC02	38.5	8.07	0.286	9.0%	36.8-42
322	GS11	PH	BnC02	40.5	5.92	3.227	7.6%	38.8-42.8
323	GS11	MIY	BnC03	58.5	8.83	-0.359	11.9%	56.8-61.8
324	GS11	MIY	BnC03	72.9	5.39	0.283	7.0%	72.4-75
325	GS11	BSP	BnC03	89.1	6.03	-0.012	11.9%	85.6-93.1
326	GS11	BAL	BnC04	12.1	3.48	-1.661	5.3%	11.5-14.1
327	GS11	MIY	BnC04	109.9	6.90	0.237	9.1%	106.3-111.1
328	GS11	MIYP	BnC04	149.9	7.41	0.023	9.9%	149.4-150.6
329	GS11	PH	BnC05	58.8	3.50	2.426	3.7%	56.1-60.6
330	GS11	SSN	BnC05	137.3	3.80	12.154	7.8%	132.8-138.2
331	GS11	TSW	BnC06	54.8	9.75	0.127	10.3%	53.7-55.4
332	GS11	BHP	BnC07	15.3	3.74	0.014	3.4%	12-17.1
333	GS11	BN	BnC07	21.9	11.68	-0.416	16.0%	20.3-23.2
334	GS11	MISNP	BnC07	21.9	4.33	0.012	7.6%	14.4-23.7
335	GS11	BTL	BnC07	24.8	8.43	-24.384	16.4%	23.8-26.2
336	GS11	BSP	BnC07	29.2	7.45	-0.015	11.8%	28.9-33.5
337	GS11	BS	BnC07	30.4	5.86	-1.946	8.3%	28.7-34.5
338	GS11	TSW	BnC07	60.4	10.66	0.153	13.5%	59.6-64.5

339	GS11	BSP	BnC08	78.3	7.94	0.015	15.2%	77.2-79.4
340	GS11	BS	BnC08	100.6	7.39	1.987	11.5%	98.7-103.9
341	GS11	SSN	BnC08	113.0	11.97	21.754	26.4%	109.5-115
342	GS11	MILP	BnC08	129.7	9.79	-0.020	6.8%	128.7-131.2
343	GS11	MIL	BnC08	130.0	6.70	-2.411	8.0%	128.7-132
344	GS11	MISN	BnC08	131.0	9.86	-3.248	14.8%	128.8-133
345	GS11	BSN	BnC09	136.4	13.48	-153.569	30.4%	135.9-136.4
346	GS11	PSN	BnC09	136.4	13.17	-156.147	29.1%	135.9-136.4

Supplementary Table S5. Detailed information of unique QTLs.

ID	Environment	Trait	LG	Peak	LOD	R^2	A	Interval
1	/WH09	/PH	BnA01	0	5.74	8.3	-2.6966	0-2.3
2	/GS11/HG10	/PH/BAL/MIL/MIYP	BnA01	8.07				7.41-8.74
3	/WH10/HG10/WH09	/MIY/SSN	BnA01	15.8				15.19-16.41
4	/WH10	/TSW	BnA01	19.6	8.77	10.8	-0.144	19.2-20.7
5	/WH09/WH10	/TSW/MIL/SSN	BnA01	25.7				25.16-26.24
6	/WH10/HG10	/MIY/MILP/PY	BnA01	29.04				28.69-29.38
7	/GS11	/BH/BHP	BnA01	31.9				30.68-33.12
8	/WH10	/MIL	BnA01	49.5	4.11	5.6	-1.9615	49.5-51.8
9	/WH10	/BAL	BnA01	57.1	4.18	3.7	-2.1314	53.7-60.4
10	/WH09/HG10	/BSN/BSP/TSW	BnA01	124.85				122.22-127.47
11	/WH09/HG10	/BS/BTL/BN/BSN/MISNP/MIYP/SSN	BnA02	12.56				11.45-13.67
12	/GS11/WH09	/PSN	BnA02	16.66				15.94-17.37
13	/GS11/WH10	/BH/BHP/MILP	BnA02	18.6				18.47-18.73
14	/WH09/WH10/GS11	/MISN/PY/BY/MIL/BAL	BnA02	21.84				21.63-22.06
15	/WH09/WH10/HG10/GS11	/TSW/PY/PH/BN/BHP	BnA02	24.39				23.96-24.81
16	/WH09	/MILP	BnA02	26.5	12.95	18.3	0.0168	25.6-26.8
17	/HG10	/BSN	BnA02	31.41	3.41	6.9	-16.264	29.8-32.8
18	/WH10	/BH/BHP	BnA02	53.9				53.07-54.73
19	/WH09/HG10	/BSP/PH	BnA02	56.76				56.29-57.23
20	/WH10	/BAL	BnA02	63.6	9.61	9.1	-3.5622	63.3-66.1
21	/GS11	/SSN	BnA02	66.6	7.75	15.2	-17.2666	65.6-67.8

22	/WH09	/BHP	BnA02	71.7	6.41	9.0	0.0182	71.5-72.7
23	/GS11/HG10	/MISN/PSN	BnA02	96.95				96.11-97.8
24	/GS11	/BHP	BnA03	0	0.00	2.8	0.01221	0-4.1
25	/WH10	/PY	BnA03	13.5	3.77	5.4	0.7151	12-16.3
26	/WH09	/PY	BnA03	20.1	3.56	5.6	0.9462	18.7-25.3
27	/WH10	/TSW	BnA03	31.4	4.54	11.3	0.1469	26.6-35.4
28	/WH09/HG10	/TSW	BnA03	43.51				42.14-44.87
29	/GS11	/PH	BnA03	52.4	11.07	15.7	-4.4827	49.8-55.2
30	/WH10/HG10	/SSN/BHP/MIL	BnA03	71.26				70.52-72
31	/HG10	/BY/BSN/MISNP	BnA03	92.1				88.88-95.32
32	/WH09	/TSW	BnA03	124.7	3.69	6.6	0.1296	123.3-130.8
33	/HG10	/BAL	BnA03	133.7	4.43	13.4	2.9933	128.1-139.4
34	/GS11	/PH	BnA03	142	3.91	4.6	3.3724	141.4-143
35	/GS11	/MISN	BnA03	147.6	3.98	5.8	2.717	146.2-150.9
36	/WH10	/BSP	BnA03	153.8	7.67	15.1	-0.0192	153.4-156.4
37	/WH10/HG10	/BN/BTL	BnA03	163.4				162.73-164.07
38	/WH10/HG10	/MISN/MIY	BnA03	175.77				174.59-176.94
39	/WH10	/BSP	BnA03	183.9	4.32	8.6	0.0109	182-186.2
40	/GS11	/SSN	BnA04	3.2	6.25	12.5	14.8294	1.5-4.5
41	/HG10	/MISN	BnA04	22.2	3.51	6.0	2.372	14.5-31.8
42	/WH09/WH10/HG10	/MILP/BH/BHP	BnA04	41.78				40.9-42.65
43	/WH10	/PY	BnA04	49.5	5.57	9.7	-0.8747	47.2-50.8
44	/WH10/GS11	/BY/MIYP/MIY	BnA04	56.99				56.06-57.91
45	/WH10	/PY	BnA05	20.4	9.38	14.4	1.0819	19.7-22.5

46	/WH10/GS11	/MIL/MIYP	BnA05	45.7				44.46-46.94
47	/WH09	/MIYP	BnA05	59.5	6.31	10.1	0.0185	58.2-60.8
48	/WH09	/MILP	BnA05	62.5	3.44	4.3	-0.0089	60.5-62.6
49	/WH09/HG10	/MIY/SSN	BnA06	0.3				0-0.84
50	/WH10	/MIL	BnA06	8.2	3.28	3.8	1.6382	7.3-9.9
51	/GS11	/MIL	BnA06	24.9	6.30	7.8	2.1935	20.8-28.1
52	/HG10	/BS	BnA06	34.7	7.97	11.3	2.2542	34.1-35.9
53	/GS11/WH09	/SSN	BnA06	58.32				57.09-59.55
54	/WH10	/PSN/BSN	BnA06	64.07				62.79-65.35
55	/HG10	/PH	BnA07	8.3	6.76	11.8	4.231	6.8-12.1
56	/WH10	/MIL	BnA07	40.9	4.92	5.6	-2.2777	39.6-43.1
57	/GS11	/TSW	BnA07	68.5	5.23	10.0	-0.1406	63.3-73.3
58	/WH09	/PH	BnA07	81.5	6.20	9.0	-2.8387	80.9-83.4
59	/HG10	/BAL/TSW	BnA07	86.82				85.8-87.85
60	/WH09	/BS	BnA07	93.1	7.96	11.6	-2.4669	92.5-94.2
61	/HG10	/TSW	BnA07	96.2	8.95	9.4	-0.1258	95.3-96.7
62	/WH10	/MIYP	BnA07	98.6	4.89	9.6	0.011	97.9-98.9
63	/GS11	/BN/MIY	BnA07	101.8				100.83-102.77
64	/WH09/HG10	/BTL/TSW	BnA07	111.82				110.83-112.82
65	/WH09	/TSW	BnA07	121.9	5.28	6.2	-0.1213	121.5-122.2
66	/WH09/WH10	/BAL/PH	BnA08	1.32				0-2.72
67	/WH09/WH10/HG10/GS11	/MISNP/BY/MIYP/BSN/PH/PSN	BnA08	15.96				15.53-16.39
68	/GS11	/BHP/PH	BnA08	27.6				26.1-29.1
69	/WH09/HG10	/BTL/BSN/PH/PSN	BnA08	30.09				29.75-30.42

70	/WH09/WH10/HG10/GS11	/BH/BN/BS/BTL/MILP	BnA08	36.82					35.82-37.82
71	/GS11	/BH	BnA08	41.9	7.20	6.2	-2.7982		41.3-43
72	/WH10	/PH	BnA08	43.3	5.23	7.6	-4.4135		43.1-46.5
73	/HG10/GS11	/BN/SSN	BnA08	46.31					45.35-47.27
74	/GS11	/MISN	BnA08	52.5	5.57	7.6	2.3977		51.3-54.3
75	/WH09	/BSP	BnA08	65.2	3.45	5.2	-0.009767		59-68.5
76	/HG10	/BS/BSP	BnA09	19.7					17.82-21.58
77	/WH09	/BSP	BnA09	26.7	11.16	19.0	-0.0184		23.8-27.7
78	/WH09/GS11	/MIL/MISN	BnA09	44.67					41.7-47.65
79	/GS11/WH10/HG10	/PY/TSW/SSN	BnA09	52.95					52.26-53.64
80	/HG10/WH10	/MIL/MISN/MILP	BnA09	62.47					61.75-63.19
81	/WH09	/MIY/MIL/MILP	BnA09	79.14					78.1-80.19
82	/GS11	/BAL	BnA09	89.2	3.91	6.0	1.6847		88-90.8
83	/WH10	/MIL	BnA10	39.1	4.92	5.6	-2.1297		34.1-42.4
84	/HG10	/BN	BnA10	43.1	6.29	9.4	0.3036		42.3-44
85	/WH10/HG10/GS11	/BS/MIY/SSN/PH/BH/MILP/BHP/BY/PY	BnA10	47.02					46.6-47.43
86	/WH10	/MISNP	BnA10	56.2	3.78	6.9	-0.0086		55.2-57
87	/HG10/GS11	/BS/BY	BnA10	62.3					60.68-63.92
88	/WH10/HG10/GS11	/SSN/TSW/MILP	BnC01	0.07					0-0.77
89	/GS11/WH09/HG10	/BN	BnC01	4.09					3.47-4.72
90	/WH10	/MISN	BnC01	53.7	4.72	7.2	2.3199		51.9-55.5
91	/GS11	/TSW	BnC01	56.3	6.96	7.1	-0.1069		54.3-57.4
92	/HG10	/MIYP	BnC01	71.4	5.22	9.6	-0.0104		68.7-74.8
93	/WH10	/PY	BnC02	2.0	4.33	5.5	1.0655		1-7.3

94	/WH09/WH10/HG10	/MIY/BH/MISN	BnC02	10.18				8.14-12.21
95	/WH09/GS11	/BN	BnC02	35.39				34.63-36.16
96	/GS11	/PH	BnC02	40.5	5.92	7.6	3.2266	38.8-42.8
97	/WH10	/BN	BnC02	42.8	9.01	10.1	0.4778	42.2-43.6
98	/HG10	/BSP	BnC02	51.3	3.51	4.9	-0.0088	50-53.4
99	/WH09/WH10	/MISN/BSP	BnC02	57.47				53.47-61.46
100	/WH10	/PY	BnC02	97.2	3.23	4.6	-0.5862	97.2-99.1
101	/WH09	/BAL	BnC03	38.2	5.24	8.0	-1.7267	27.8-43.5
102	/GS11	/MIY	BnC03	58.5	8.83	11.9	-0.3587	56.8-61.8
103	/GS11	/MIY	BnC03	72.9	5.39	7.0	0.2831	72.4-75
104	/WH10	/MIYP	BnC03	77.8	3.54	7.5	0.0127	74.5-79.6
105	/WH10	/MIY	BnC03	85.1	3.57	4.8	0.1475	81.5-88.6
106	/GS11	/BSP	BnC03	89.1	6.03	11.9	-0.0123	85.6-93.1
107	/WH09/HG10	/BTL/BHP	BnC03	94.06				92.84-95.27
108	/WH09/WH10/HG10	/BS/SSN/BH	BnC03	103.26				102.91-103.61
109	/HG10	/BS	BnC04	2.9	5.45	7.5	1.8609	1-5.6
110	/WH09/GS11	/BH/BAL	BnC04	11.63				11.02-12.25
111	/WH10/HG10	/BH/PH/MISNP/BHP/MIYP/BSN	BnC04	18.6				18.21-19
112	/HG10	/MIY/BY/PY	BnC04	55.38				54.97-55.8
113	/WH10	/BY	BnC04	59.3	4.97	8.3	0.7891	58-60.3
114	/WH09	/PH	BnC04	65.4	8.15	12.1	3.3957	64.2-67.4
115	/WH10	/MIY	BnC04	69.4	3.65	5.6	0.2099	66.5-74.6
116	/WH09	/BY/PY	BnC04	89.7				89.19-90.21
117	/WH09/WH10/GS11	/MIY/BH/MISNP/PY	BnC04	106.3				105.02-107.58

118	/WH09/WH10	/BHP	BnC04	117.54				114.41-120.68
119	/WH10	/MILP	BnC04	122	3.95	4.7	-0.0183	119-124.7
120	/WH09	/BSP	BnC04	140.7	4.39	6.0	-0.0113	139.2-141.6
121	/WH10	/MILP	BnC04	147.3	3.77	4.1	0.0169	145.9-148.8
122	/GS11	/MIYP	BnC04	149.9	7.41	9.9	0.0228	149.4-150.6
123	/HG10	/PSN/BSN	BnC05	9.8				9.2-10.4
124	/WH09/GS11	/BY/PY/PH/BAL	BnC05	59.44				58.4-60.4
125	/WH09/WH10/HG10	/TSW/MILP/MISNP	BnC05	99.75				97.6-101.9
126	/WH10/HG10	/BN/MIY	BnC05	115.48				113.7-117.2
127	/GS11	/SSN	BnC05	137.3	3.80	7.8	12.154	132.8-138.2
128	/WH09	/MISN	BnC06	2.9	5.02	5.3	2.5455	1.9-3.0
129	/HG10	/SSN	BnC06	5.5	4.50	6.3	-8.8749	4.1-8.8
130	/WH09	/MIL	BnC06	14.8	7.30	14.2	2.0932	11.4-19.0
131	/HG10	/TSW	BnC06	31.3	12.93	15.4	0.1581	29.8-32.2
132	/WH09/WH10	/BN/BS/TSW	BnC06	39.72				39.4-40.0
133	/WH10/HG10	/MILP/MIL	BnC06	43.38				42.7-44.1
134	/WH09/WH10/HG10	/BAL/BH/BHP/PH	BnC06	45.5				44.9-46.1
135	/WH09	/MILP	BnC06	49.1	14.65	20.8	0.0183	48.8-49.1
136	/WH10/HG10/GS11	/BAL/BH/BHP/MILP/TSW	BnC06	54.8				54.4-55.2
137	/WH09/WH10/GS11	/MISN/TSW/BHP	BnC07	14.21				12.79-15.63
138	/WH09/HG10	/BH/BTL	BnC07	18.48				16.83-20.12
139	/GS11	/BN/MISNP	BnC07	21.9				20.52-23.28
140	/GS11	/BTL	BnC07	24.8	8.43	16.4	-24.3842	23.8-26.2
141	/HG10	/MIY	BnC07	26.2	4.08	6.4	0.2141	25.1-28.5

142	/GS11	/BSP/BS	BnC07	29.66				27.86-31.47
143	/GS11/WH09	/TSW	BnC07	61.3				59.25-63.35
144	/WH10	/BTL	BnC07	68.6	4.88	7.5	-23.0178	66.6-69.7
145	/HG10	/TSW	BnC07	75.8	3.78	3.4	0.0706	75.2-76.7
146	/WH09	/BN/BSN/PSN/MIYP	BnC08	60.39				59.55-61.22
147	/WH09/HG10	/MISNP/MISN/BHP	BnC08	74.06				72.5-75.62
148	/HG10/GS11	/BH/BSP	BnC08	77.99				77.23-78.75
149	/GS11	/BS	BnC08	100.6	7.39	11.5	1.9871	98.7-103.9
150	/WH10/GS11	/BSN/PSN/SSN/MISN	BnC08	115.76				114.48-117.03
151	/WH09/HG10	/SSN/PSN	BnC08	126.59				125.43-127.74
152	/WH09/HG10/GS11	/BSN/MILP/MIL/MISN	BnC08	129.79				129.26-130.32
153	/HG10	/PSN	BnC08	133	9.07	15.7	-24.8761	131.1-134
154	/HG10	/MILP	BnC08	135	3.77	4.4	-0.0091	133.7-135.3
155	/WH10	/BY/PY	BnC08	137.6				136.87-138.33
156	/HG10	/BSP	BnC08	143.7	7.78	11.3	0.0128	141.6-144.2
157	/HG10	/BSP	BnC09	18.7	5.36	7.9	0.0109	16.3-20.3
158	/WH10	/PH	BnC09	88.2	4.54	7.5	5.0676	84.6-91
159	/WH09	/MIYP	BnC09	97.9	7.18	12.0	0.039	96.3-99.2
160	/HG10	/BN/BS/BTL	BnC09	125.36				124.67-126.04
161	/HG10	/BAL	BnC09	130.2	3.63	6.6	-4.0439	128.4-131.3
162	/GS11	/BSN/PSN	BnC09	136.4				136.22-136.58
163	/WH10	/BAL	BnC09	159.5	5.11	4.7	3.9132	157.7-161.8

Supplementary Table S6. Detailed information and references of the genes for plant architecture

Gene (Reference)	Species	Pathway	Copy number
			B. napus
ARF1[1]	*Arabidopsis*	Aux/IAA	3
ARF19[2]	*Arabidopsis*	Aux/IAA	6
ARF2[2]	*Arabidopsis*	Aux/IAA	8
ARF7[2]	*Arabidopsis*	Aux/IAA	2
ARGOS[3]	*Arabidopsis*	Aux/IAA	3
AtPGP1[4]	*Arabidopsis*	Aux/IAA	3
AtPGP19[4]	*Arabidopsis*	Aux/IAA	6
AtPGP4[5]	*Arabidopsis*	Aux/IAA	6
AUX1[6]	*Arabidopsis*	Aux/IAA	6
AVP1[7]	*Arabidopsis*	Aux/IAA	8
AXR1[8]	*Arabidopsis*	Aux/IAA	9
AXR2[9]	*Arabidopsis*	Aux/IAA	7
AXR3[10]	*Arabidopsis*	Aux/IAA	6
AXR6[9]	*Arabidopsis*	Aux/IAA	8
BUD1[11]	*Arabidopsis*	Aux/IAA	4
BUD2[12]	*Arabidopsis*	Aux/IAA	5
IAA14[13]	*Arabidopsis*	Aux/IAA	7
IAA18[14]	*Arabidopsis*	Aux/IAA	5
IAA28[15]	*Arabidopsis*	Aux/IAA	4
IAA3[16]	*Arabidopsis*	Aux/IAA	4
IAA6[17]	*Arabidopsis*	Aux/IAA	2
IAA9[18]	*Arabidopsis*	Aux/IAA	9
LAX1[19]	*Arabidopsis*	Aux/IAA	6
LAX2[19]	*Arabidopsis*	Aux/IAA	7
LAX3[19]	*Arabidopsis*	Aux/IAA	4
MP[2]	*Arabidopsis*	Aux/IAA	6
PID[20]	*Arabidopsis*	Aux/IAA	3
PIN1[21]	*Arabidopsis*	Aux/IAA	4
PIN2[22]	*Arabidopsis*	Aux/IAA	6
PIN6[23]	*Arabidopsis*	Aux/IAA	2
PIN7[23]	*Arabidopsis*	Aux/IAA	6

TIR1[24]	*Arabidopsis*	Aux/IAA	6
UCH1[25]	*Arabidopsis*	Aux/IAA	2
UCH2[25]	*Arabidopsis*	Aux/IAA	2
BR2[26]	Maize	Aux/IAA	6
DAD1[27]	*Petunia hybrida*	Aux/IAA	2
D10[28]	Rice	Aux/IAA	2
IAA20[29]	Rice	Aux/IAA	0
SAMDC1[12,30]	Rice	Aux/IAA	7
SoDW3[26]	Sorghum	Aux/IAA	6
**Sub total**	**40 (34)**	**Aux/IAA**	**198 (175)**
AHK1[31]	*Arabidopsis*	CK	2
AHK2[31]	*Arabidopsis*	CK	4
AHK3[31]	*Arabidopsis*	CK	2
AHK4[31]	*Arabidopsis*	CK	2
AHK5[31]	*Arabidopsis*	CK	7
AMP1[32]	*Arabidopsis*	CK	3
ARR15[33]	*Arabidopsis*	CK	4
ARR5[34]	*Arabidopsis*	CK	8
ARR6[33]	*Arabidopsis*	CK	8
ARR7[33]	*Arabidopsis*	CK	4
BP[35]	*Arabidopsis*	CK	3
CKX2[36]	*Arabidopsis*	CK	4
CLV1[37]	*Arabidopsis*	CK	2
CLV2[38]	*Arabidopsis*	CK	2
CLV3[39]	*Arabidopsis*	CK	0
IPT5[35]	*Arabidopsis*	CK	4
IPT7[35]	*Arabidopsis*	CK	3
KN1[40]	*Arabidopsis*	CK	2
STM[41]	*Arabidopsis*	CK	3
WUS[42]	*Arabidopsis*	CK	5
LOG[43]	Rice	CK	29
OsCKX2[44]	Rice	CK	0
**Sub total**	**22 (20)**	**CK**	**101 (72)**
DWF1A[45]	*Arabidopsis*	GA	4
DWF1B[45]	*Arabidopsis*	GA	6

DWF1C[45]	*Arabidopsis*	GA	2
GA20ox 1[46]	*Arabidopsis*	GA	10
GA20ox 2[46]	*Arabidopsis*	GA	10
GA20ox 3[46]	*Arabidopsis*	GA	6
GA20ox 4[47]	*Arabidopsis*	GA	5
GA20ox 5[47]	*Arabidopsis*	GA	3
GA2ox 1[48]	*Arabidopsis*	GA	7
GA2ox 2[48]	*Arabidopsis*	GA	6
GA2ox 3[48]	*Arabidopsis*	GA	5
GA2ox 4[48]	*Arabidopsis*	GA	2
GA2ox 5[48]	*Arabidopsis*	GA	0
GA2ox 6[48]	*Arabidopsis*	GA	4
GA2ox 7[48]	*Arabidopsis*	GA	2
GA2ox 8[48]	*Arabidopsis*	GA	7
GA3ox 1[47]	*Arabidopsis*	GA	4
GA3ox 2[47]	*Arabidopsis*	GA	5
GA3ox 3[47]	*Arabidopsis*	GA	2
GA3ox 4[47]	*Arabidopsis*	GA	2
GAI[49]	*Arabidopsis*	GA	0
KTN1[50]	*Arabidopsis*	GA	4
RGA[51]	*Arabidopsis*	GA	4
RGL1[52]	*Arabidopsis*	GA	2
SHI[53]	*Arabidopsis*	GA	2
SLY1[54]	*Arabidopsis*	GA	2
SLY2[54]	*Arabidopsis*	GA	6
SPY[55]	*Arabidopsis*	GA	4
D8[56]	Maize	GA	10
PHOR[57]	Maize	GA	0
BC12[58]	Rice	GA	12
D1[59]	Rice	GA	2
D62[60]	Rice	GA	4
DGL1[50]	Rice	GA	4
EUI[61]	Rice	GA	0
GSR1[62]	Rice	GA	0
GID1[63]	Rice	GA	10

GID2[64]	Rice	GA	0
Os20ox2[65]	Rice	GA	10
SD1[66]	Rice	GA	0
SLR1[64]	Rice	GA	6
RHT1[67]	Wheat	GA	6
**Sub total**	**42 (28)**	**GA**	**180 (116)**
ARL[68]	*Arabidopsis*	BR	6
BAK1[69]	*Arabidopsis*	BR	11
BES1[70]	*Arabidopsis*	BR	6
BIN2[71]	*Arabidopsis*	BR	7
BKI1[72]	*Arabidopsis*	BR	3
BRI1[69]	*Arabidopsis*	BR	7
BSU1[72]	*Arabidopsis*	BR	0
BZR1[73]	*Arabidopsis*	BR	0
CPD[74]	*Arabidopsis*	BR	7
DWF4[74]	*Arabidopsis*	BR	4
GRAS19[75]	Maize	BR	3
BU1[76]	Rice	BR	4
CYP724B1[77]	Rice	BR	4
CYP90D2[78]	Rice	BR	3
D11[78]	Rice	BR	4
D2[79]	Rice	BR	3
ILA1[80]	Rice	BR	13
ILI1[81]	Rice	BR	0
LIC[82]	Rice	BR	2
MADS22[83]	Rice	BR	4
OsMDP1[84]	Rice	BR	2
RAVL1[85]	Rice	BR	0
TUD1[86]	Rice	BR	4
XIAO[87]	Rice	BR	4
GRAS7[75]	*Solanum* [illegible]	BR	16
**Sub total**	**25 (10)**	**BR**	**117 (51)**
BRC1[88]	*Arabidopsis*	SL	1
BRC2[88]	*Arabidopsis*	SL	6
IAA12[89]	*Arabidopsis*	SL	4

MAX1[90]	*Arabidopsis*	SL	1
MAX2[91]	*Arabidopsis*	SL	1
MAX3[92]	*Arabidopsis*	SL	2
MAX4[93]	*Arabidopsis*	SL	3
TB1[94]	Maize	SL	0
DAD2[95]	*Petunia hybrida*	SL	7
RMS1[93]	*Pisum sativum*	SL	2
RMS4[96]	*Pisum sativum*	SL	0
RMS5[96]	*Pisum sativum*	SL	2
D14[97]	Rice	SL	7
D17[98]	Rice	SL	2
D27[99]	Rice	SL	2
D3[26]	Rice	SL	4
D53[100,101]	Rice	SL	6
D88[102]	Rice	SL	7
HTD2[103]	Rice	SL	7
OsTB1[104]	Rice	SL	0
SLB1[105]	Rice	SL	2
SLB2[105]	Rice	SL	2
**Sub total**	**22 (7)**	**SL**	**68 (18)**
APC10[106]	*Arabidopsis*	CC	2
APC13[107]	*Arabidopsis*	CC	2
APC2[108]	*Arabidopsis*	CC	3
APC4[109]	*Arabidopsis*	CC	2
APC6[110]	*Arabidopsis*	CC	3
APC8[107]	*Arabidopsis*	CC	5
AtD27[111]	*Arabidopsis*	CC	2
CCS52A1[112]	*Arabidopsis*	CC	9
CCS52A2[112]	*Arabidopsis*	CC	8
CCS52B[112]	*Arabidopsis*	CC	2
Cdc20.1[113]	*Arabidopsis*	CC	7
Cdc20.2[113]	*Arabidopsis*	CC	7
Cdc20.3[113]	*Arabidopsis*	CC	1
Cdc20.4[113]	*Arabidopsis*	CC	3
Cdc20.5[113]	*Arabidopsis*	CC	3

CDC27A[114]	*Arabidopsis*	CC	2
CDC27B[114]	*Arabidopsis*	CC	3
CKI1[115]	*Arabidopsis*	CC	4
CYC2[116]	*Arabidopsis*	CC	4
CYCB1;1[117]	*Arabidopsis*	CC	2
CYCB2;2[117]	*Arabidopsis*	CC	6
CYCD2;1[118]	*Arabidopsis*	CC	2
CycD3[118]	*Arabidopsis*	CC	7
GIG1[119]	*Arabidopsis*	CC	0
UVI4[120]	*Arabidopsis*	CC	2
TAD1[121]	Rice	CC	9
**Sub total**	**26 (25)**	**CC**	**100 (91)**
ABI3[122]	*Arabidopsis*	TF	2
AIL5[123]	*Arabidopsis*	TF	2
AIL6[123]	*Arabidopsis*	TF	9
AIL7[123]	*Arabidopsis*	TF	7
AN3[124]	*Arabidopsis*	TF	5
ANT[125]	*Arabidopsis*	TF	6
CAL[126]	*Arabidopsis*	TF	2
CUC1[127]	*Arabidopsis*	TF	6
CUC2[127]	*Arabidopsis*	TF	2
CUC3[127]	*Arabidopsis*	TF	4
GRF1[128]	*Arabidopsis*	TF	3
GRF3[129]	*Arabidopsis*	TF	5
GRF5[129]	*Arabidopsis*	TF	6
JAW[130]	*Arabidopsis*	TF	0
LAS[131]	*Arabidopsis*	TF	2
LFY[132]	*Arabidopsis*	TF	4
RAM1[133]	*Arabidopsis*	TF	3
RAX1[133]	*Arabidopsis*	TF	4
RAX2[133]	*Arabidopsis*	TF	4
RAX3[133]	*Arabidopsis*	TF	8
REV[134]	*Arabidopsis*	TF	6
TFL1[135]	*Arabidopsis*	TF	5
BA1[136]	Maize	TF	5

NAM[137]	*Petunia hybrida*	TF	2
LF[138]	*Pisum sativum*	TF	17
MADS5[139]	*Rice*	TF	16
MADS7[139]	*Rice*	TF	15
MADS55[83]	Rice	TF	0
MADS56[139]	*Rice*	TF	9
MADS57[140]	*Rice*	TF	9
MOC1[141]	Rice	TF	0
OsLAX1[142]	Rice	TF	0
OsLAX2[142]	Rice	TF	0
PCF1[143]	Rice	TF	0
PROG1[144]	Rice	TF	0
Ls[145]	Tomato	TF	2
SP[146]	Tomato	TF	19
**Sub total**	**37 (22)**	**TF**	**189 (95)**
AG[147]	*Arabidopsis*	Other	6
AP1[148]	*Arabidopsis*	Other	6
AP3[149]	*Arabidopsis*	Other	4
ASK1[150]	*Arabidopsis*	Other	3
CNA[151]	*Arabidopsis*	Other	2
EMF1[152]	*Arabidopsis*	Other	4
ER[153]	*Arabidopsis*	Other	3
ERA1[154]	*Arabidopsis*	Other	3
LOB[155]	*Arabidopsis*	Other	4
PHAB[156]	*Arabidopsis*	Other	3
PHAV[156]	*Arabidopsis*	Other	2
PNH[157]	*Arabidopsis*	Other	3
SEU[158]	*Arabidopsis*	Other	0
SPL9[159]	*Arabidopsis*	Other	4
SPS1[160]	*Arabidopsis*	Other	3
STIP[161]	*Arabidopsis*	Other	4
SYD[162]	*Arabidopsis*	Other	0
FEA2[163]	Maize	Other	0
RA1[164]	Maize	Other	0
RA2[165]	Maize	Other	0

RA3[166]	Maize	Other	20
TD1[167]	Maize	Other	3
APO1[168]	Rice	Other	0
APO2[169]	Rice	Other	2
DEP1[170]	Rice	Other	0
FON1[171]	Rice	Other	10
FON4[172]	Rice	Other	0
FZP[173]	Rice	Other	0
IPA1[174]	Rice	Other	0
LA1[175]	Rice	Other	0
LPA1[176]	Rice	Other	0
MST4[139]	Rice	Other	4
NYC3[177]	Rice	Other	4
OGR1[178]	Rice	Other	2
OsDW3[26]	Rice	Other	4
OSH1[179]	Rice	Other	4
OsSPL14[180]	Rice	Other	0
SP1[146]	Rice	Other	3
Spk(t)[181]	Rice	Other	8
TAC1[182]	Rice	Other	0
TAW1[183]	Rice	Other	9
WFP[174]	Rice	Other	0
**Sub total**	**42 (17)**	**Other**	**127 (54)**
**Total**	**256 (163)**		**1080 (672)**

References

1. Ulmasov, T., Hagen, G. & Guilfoyle, T. J. *ARF1*, a transcription factor that binds to auxin response elements. *Science* **276,** 1865-1868 (1997).

2. Okushima, Y. *et al*. Functional genomic analysis of the *AUXIN RESPONSE FACTOR* gene family members in *Arabidopsis thaliana*: unique and overlapping

functions of *ARF7* and *ARF19*. *Plant Cell* **17,** 444-463 (2005).

3. Hu, Y., Xie, Q. & Chua, N.H. The Arabidopsis auxin-inducible gene *ARGOS* controls lateral organ size. *Plant Cell* **15,** 1951-1961 (2003).

4. Bouchard, R. *et al*. Immunophilin-like *TWISTED DWARF1* modulates auxin efflux activities of Arabidopsis P-glycoproteins. *J. Biol. Chem.* **281,** 30603-30612 (2006).

5. Terasaka, K. *et al*. *PGP4*, an ATP binding cassette P-glycoprotein, catalyzes auxin transport in *Arabidopsis thaliana* roots. *Plant Cell* **17,** 2922-2939 (2005).

6. Bennett, M. J. *et al*. Arabidopsis *AUX1* gene: a permease-like regulator of root gravitropism. *Science* **273,** 948-950 (1996).

7. Li, J. *et al*. Arabidopsis H+-PPase AVP1 regulates auxin-mediated organ development. *Science* **310,** 121-125 (2005).

8 Leyser, H. O. *et al*. Arabidopsis auxin-resistance gene *AXR1* encodes a protein related to ubiquitin-activating enzyme E1. *Nature* 364, 161-164 (1993).

9. Nagpal, P. *et al*. AXR2 encodes a member of the Aux/IAA protein family. *Plant Physiol.* **123,** 563-574 (2000).

10. Leyser, H., Pickett, F. B., Dharmasiri, S. & Estelle, M. Mutations in the *AXR3* gene of Arabidopsis result in altered auxin response including ectopic expression from the SAUR-AC1 promoter. *Plant J.* **10,** 403-413 (1996).

11. Dai, Y. *et al*. Increased expression of *MAP KINASE KINASE7* causes deficiency in polar auxin transport and leads to plant architectural abnormality in Arabidopsis. *Plant Cell* **18,** 308-320 (2006).

12. Cui, X. *et al*. The *BUD2* mutation affects plant architecture through altering

cytokinin and auxin responses in Arabidopsis. *Cell Res.* **20,** 576-586 (2010).

13. Fukaki, H., Tameda, S., Masuda, H. & Tasaka, M. Lateral root formation is blocked by a gain-of-function mutation in the *SOLITARY-ROOT/IAA14* gene of Arabidopsis. *Plant J.* **29,** 153-168 (2002).

14. Reed, J. W. Roles and activities of Aux/IAA proteins in Arabidopsis. *Trends Plant Sci.* **6,** 420-425 (2001).

15. Rogg, L. E., Lasswell, J. & Bartel, B. A gain-of-function mutation in *IAA28* suppresses lateral root development. *Plant Cell* **13,** 465-480 (2001).

16. Tian, Q. & Reed, J. Control of auxin-regulated root development by the Arabidopsis thaliana *SHY2/IAA3* gene. *Development* **126,** 711-721 (1999).

17. Kim, B. C., Soh, M. S., Kang, B. J., Furuya, M. & Nam, H. G. Two dominant photomorphogenic mutations of *Arabidopsis thaliana* identified as suppressor mutations of *hy2*. *Plant J.* **9,** 441-456 (1996).

18. Wang, H. *et al*. The tomato Aux/IAA transcription factor *IAA9* is involved in fruit development and leaf morphogenesis. *Plant Cell* **17,** 2676-2692 (2005).

19. Parry, G. *et al*. Novel auxin transport inhibitors phenocopy the auxin influx carrier mutation aux1. *Plant J.* **25,** 399-406 (2001).

20. Bögre, L., Ökrész, L., Henriques, R. & Anthony, R. G. Growth signalling pathways in Arabidopsis and the AGC protein kinases. *Trends Plant Sci.* **8,** 424-431 (2003).

21. Petrášek, J. *et al*. PIN proteins perform a rate-limiting function in cellular auxin efflux. *Science* **312,** 914-918 (2006).

22. Müller, A. *et al*. AtPIN2 defines a locus of Arabidopsis for root gravitropism control. *EMBO J.* **17,** 6903-6911 (1998).

23. Friml, J. *et al*. Efflux-dependent auxin gradients establish the apical-basal axis of Arabidopsis. *Nature* **426,** 147-153 (2003).

24. Kepinski, S. & Leyser, O. The Arabidopsis F-box protein TIR1 is an auxin receptor. *Nature* **435,** 446-451 (2005).

25. Yang, P. *et al*. Ubiquitin C-terminal hydrolases 1 and 2 affect shoot architecture in Arabidopsis. *Plant J.* **51,** 441-457 (2007).

26. Multani, D. S. *et al*. Loss of an MDR transporter in compact stalks of maize *br2* and sorghum *dw3* mutants. *Science* **302,** 81-84 (2003).

27. Napoli, C. Highly branched phenotype of the petunia *dad1-1* mutant is reversed by grafting. *Plant Physiol.* **111,** 27-37 (1996).

28. Arite, T. *et al*. *DWARF10*, an *RMS1/MAX4/DAD1* ortholog, controls lateral bud outgrowth in rice. *Plant J.* **51,** 1019-1029 (2007).

29. Meng, Y. *et al*. Genome-wide survey of rice microRNAs and microRNA-target pairs in the root of a novel auxin-resistant mutant. *Planta* **230,** 883-898 (2009).

30. Hill, J. R. & Morris, D. R. Cell-specific translational regulation of S-adenosylmethionine decarboxylase mRNA. Dependence on translation and coding capacity of the cis-acting upstream open reading frame. *J. Biol. Chem.* **268,** 726-731 (1993).

31. Nishimura, C. *et al*. Histidine kinase homologs that act as cytokinin receptors possess overlapping functions in the regulation of shoot and root growth in

Arabidopsis. *Plant Cell* **16,** 1365-1377 (2004).

32. Helliwell, C. A. *et al*. The Arabidopsis AMP1 gene encodes a putative glutamate carboxypeptidase. *Plant Cell* **13,** 2115-2125 (2001).

33. Hwang, I. & Sheen, J. Two-component circuitry in Arabidopsis cytokinin signal transduction. *Nature* **413,** 383-389 (2001).

34. Jasinski, S. *et al*. *KNOX* action in Arabidopsis is mediated by coordinate regulation of cytokinin and gibberellin activities. *Curr. Biol.* **15,** 1560-1565 (2005).

35. Yanai, O. *et al*. Arabidopsis KNOXI proteins activate cytokinin biosynthesis. *Curr. Biol.* **15,** 1566-1571 (2005).

36. Werner, T., Motyka, V., Strnad, M. & Schmülling, T. Regulation of plant growth by cytokinin. *Proc. Natl. Acad. Sci. U. S. A.* **98,** 10487-10492 (2001).

37. Clark, S. E., Williams, R. W. & Meyerowitz, E. M. The *CLAVATA1* gene encodes a putative receptor kinase that controls shoot and floral meristem size in Arabidopsis. *Cell* **89,** 575-585 (1997).

38. Kayes, J. M. & Clark, S. E. *CLAVATA2*, a regulator of meristem and organ development in Arabidopsis. *Development* **125,** 3843-3851 (1998).

39. Fletcher, J. C., Brand, U., Running, M. P., Simon, R. & Meyerowitz, E. M. Signaling of cell fate decisions by *CLAVATA3* in Arabidopsis shoot meristems. *Science* **283,** 1911-1914 (1999).

40. Rupp, H. M., Frank, M., Werner, T., Strnad, M. & Schmülling, T. Increased steady state mRNA levels of the *STM* and *KNAT1* homeobox genes in cytokinin overproducing *Arabidopsis thaliana* indicate a role for cytokinins in the shoot

apical meristem. *Plant J.* **18,** 557-563 (1999).

41. Long, J. A., Moan, E. I., Medford, J. I. & Barton, M. K. A member of the KNOTTED class of homeodomain proteins encoded by the *STM* gene of Arabidopsis. *Nature* **379,** 66-69 (1996).

42. Mayer, K. F. *et al*. Role of *WUSCHEL* in regulating stem cell fate in the Arabidopsis shoot meristem. *Cell* **95,** 805-815 (1998).

43. Kurakawa, T. *et al*. Direct control of shoot meristem activity by a cytokinin-activating enzyme. *Nature* **445,** 652-655 (2007).

44. Ashikari, M. *et al*. Cytokinin oxidase regulates rice grain production. *Science* **309,** 741-745 (2005).

45. Klahre, U. *et al*. The Arabidopsis *DIMINUTO/DWARF1* gene encodes a protein involved in steroid synthesis. *Plant Cell* **10,** 1677-1690 (1998).

46. Plackett, A. R. *et al*. Analysis of the developmental roles of the Arabidopsis gibberellin 20-oxidases demonstrates that *GA20ox1,-2,* and *-3* are the dominant paralogs. *Plant Cell* **24,** 941-960 (2012).

47. Hedden, P. & Phillips, A. L. Gibberellin metabolism: new insights revealed by the genes. *Trends Plant Sci.* **5,** 523-530 (2000).

48. Olszewski, N., Sun, T.-p. & Gubler, F. Gibberellin signaling biosynthesis, catabolism, and response pathways. *Plant Cell* **14,** S61-S80 (2002).

49. Peng, J. *et al*. The Arabidopsis *GAI* gene defines a signaling pathway that negatively regulates gibberellin responses. *Gene Dev.* **11,** 3194-3205 (1997).

50. Komorisono, M. *et al*. Analysis of the rice mutant *dwarf* and *gladius leaf 1.*

Aberrant katanin-mediated microtubule organization causes up-regulation of gibberellin biosynthetic genes independently of gibberellin signaling. *Plant Physiol.* **138,** 1982-1993 (2005).

51. Silverstone, A. L., Mak, P. Y. A., Martinez, E. C. & Sun, T. The new *RGA* locus encodes a negative regulator of gibberellin response in *Arabidopsis thaliana*. *Genetics* **146,** 1087-1099 (1997).
52. Wen, C. K. & Chang, C. Arabidopsis *RGL1* encodes a negative regulator of gibberellin responses. *Plant Cell* **14,** 87-100 (2002).
53. Fridborg, I., Kuusk, S., Moritz, T. & Sundberg, E. The Arabidopsis dwarf mutant *shi* exhibits reduced gibberellin responses conferred by overexpression of a new putative zinc finger protein. *Plant Cell* **11,** 1019-1031 (1999).
54. Steber, C. M., Cooney, S. E. & McCourt, P. Isolation of the GA-response mutant sly1 as a suppressor of *ABI1-1* in *Arabidopsis thaliana*. *Genetics* **149,** 509-521 (1998).
55. Jacobsen, S. E. & Olszewski, N. E. Mutations at the *SPINDLY* locus of Arabidopsis alter gibberellin signal transduction. *Plant Cell* **5,** 887-896 (1993).
56. Winkler, R. & Freeling, M. Analysis of the autonomy of maize *Dwarf1* action in genetic mosaics. *J. Hered.* **85,** 377-380 (1994).
57. Amador, V., Monte, E., García-Martínez, J.L. & Prat, S. Gibberellins signal nuclear import of PHOR1, a photoperiod-responsive protein with homology to *Drosophila* armadillo. *Cell* **106,** 343-354 (2001).
58. Zhang, M. *et al*. *Brittle Culm 12*, a dual-targeting kinesin-4 protein, controls

cell-cycle progression and wall properties in rice. *Plant J.* **63,** 312-328 (2010).

59. Ashikari, M., Wu, J., Yano, M., Sasaki, T. & Yoshimura, A. Rice gibberellin-insensitive dwarf mutant gene *Dwarf 1* encodes the α-subunit of GTP-binding protein. *Proc. Natl. Acad. Sci. U. S. A.* **96,** 10284-10289 (1999).

60. Li, W. *et al*. Identification and characterization of *dwarf 62*, a loss-of-function mutation in *DLT/OsGRAS-32* affecting gibberellin metabolism in rice. *Planta* **232,** 1383-1396 (2010).

61. Luo, A. *et al*. *EUI1*, encoding a putative cytochrome P450 monooxygenase, regulates internode elongation by modulating gibberellin responses in rice. *Plant Cell Physiol.* **47,** 181-191 (2006).

62. Wang, L. *et al*. *OsGSR1* is involved in crosstalk between gibberellins and brassinosteroids in rice. *Plant J.* **57,** 498-510 (2009).

63. Ueguchi-Tanaka, M. *et al*. *GIBBERELLIN INSENSITIVE DWARF1* encodes a soluble receptor for gibberellin. *Nature* **437,** 693-698 (2005).

64. Gomi, K. *et al*. GID2, an F-box subunit of the SCF E3 complex, specifically interacts with phosphorylated SLR1 protein and regulates the gibberellin-dependent degradation of *SLR1* in rice. *Plant J.* **37,** 626-634 (2004).

65. Sakamoto, T. *et al*. Expression of a gibberellin 2-oxidase gene around the shoot apex is related to phase transition in rice. *Plant Physiol.* **125,** 1508-1516 (2001).

66. Monna, L. *et al*. Positional cloning of rice semidwarfing gene, *sd-1*: rice “green revolution gene” encodes a mutant enzyme involved in gibberellin synthesis. *DNA Res.* **9,** 11-17 (2002).

67. Peng, J. *et al*. 'Green revolution' genes encode mutant gibberellin response modulators. *Nature* **400,** 256-260 (1999).

68. Hu, Y., Poh, H. M. & Chua, N. H. The Arabidopsis *ARGOS-LIKE* gene regulates cell expansion during organ growth. *Plant J.* **47,** 1-9 (2006).

69. Li, J. *et al*. *B*AK1, an Arabidopsis LRR receptor-like protein kinase, Interacts with *BRI1* and modulates brassinosteroid signaling. *Cell* **110,** 213-222 (2002).

70. Yin, Y. *et al*. *BES1* accumulates in the nucleus in response to brassinosteroids to regulate gene expression and promote stem elongation. *Cell* **109,** 181-191 (2002).

71. Zhao, J. *et al*. Two putative BIN2 substrates are nuclear components of brassinosteroid signaling. *Plant Physiol.* **130,** 1221-1229 (2002).

72. Wang, X. & Chory, J. Brassinosteroids regulate dissociation of *BKI1*, a negative regulator of *BRI1* signaling, from the plasma membrane. *Science* **313,** 1118-1122 (2006).

73. Wang, Z.Y. *et al*. Nuclear-localized *BZR1* mediates brassinosteroid-induced growth and feedback suppression of brassinosteroid biosynthesis. *Dev. Cell* **2,** 505-513 (2002).

74. Choe, S. *et al*. Overexpression of *DWARF4* in the brassinosteroid biosynthetic pathway results in increased vegetative growth and seed yield in Arabidopsis. *Plant J.* **26,** 573-582 (2001).

75. Bolle, C. The role of GRAS proteins in plant signal transduction and development. *Planta* **218,** 683-692 (2004).

76. Tanaka, A. *et al*. *BRASSINOSTEROID UPREGULATED1,* encoding a

helix-loop-helix protein, is a novel gene involved in brassinosteroid signaling and controls bending of the lamina joint in rice. *Plant Physiol.* **151,** 669-680 (2009).

77. Tanabe, S. *et al*. A novel cytochrome P450 is implicated in brassinosteroid biosynthesis via the characterization of a rice dwarf mutant, *dwarf11*, with reduced seed length. *Plant Cell* **17,** 776-790 (2005).

78. Sakamoto, T. *et al*. Erect leaves caused by brassinosteroid deficiency increase biomass production and grain yield in rice. *Nat. Biotechnol.* **24,** 105-109 (2006).

79. Hong, Z. *et al*. A rice brassinosteroid-deficient mutant, *ebisu dwarf* (*d2*), is caused by a loss of function of a new member of cytochrome P450. *Plant Cell* **15,** 2900-2910 (2003).

80. Ning, J., Zhang, B., Wang, N., Zhou, Y. & Xiong, L. Increased leaf angle1, a Raf-like MAPKKK that interacts with a nuclear protein family, regulates mechanical tissue formation in the lamina joint of rice. *Plant Cell* **23,** 4334-4347 (2011).

81. Zhang, L.-Y. *et al*. Antagonistic HLH/bHLH transcription factors mediate brassinosteroid regulation of cell elongation and plant development in rice and Arabidopsis. *Plant Cell* **21,** 3767-3780 (2009).

82. Zhang, C. *et al*. Dynamics of brassinosteroid response modulated by negative regulator *LIC* in rice. *PLoS Genet.* **8,** e1002686 (2012).

83. Lee, S., Choi, S. C. & An, G. Rice SVP-group MADS-box proteins, OsMADS22 and OsMADS55, are negative regulators of brassinosteroid responses. *Plant J.* **54,** 93-105 (2008).

84. Duan, K. *et al*. A brassinolide-suppressed rice MADS-box transcription factor, *OsMDP1*, has a negative regulatory role in BR signaling. *Plant J.* **47,** 519-531 (2006).

85. Je, B. I. *et al*. RAV-Like1 maintains brassinosteroid homeostasis via the coordinated activation of *BRI1* and biosynthetic genes in rice. *Plant Cell* **22,** 1777-1791 (2010).

86. Hu, X. *et al*. The U-box E3 ubiquitin ligase TUD1 functions with a heterotrimeric G α subunit to regulate brassinosteroid-mediated growth in rice. *PLoS Genet.* **9,** e1003391 (2013).

87. Jiang, Y. *et al*. *XIAO* is involved in the control of organ size by contributing to the regulation of signaling and homeostasis of brassinosteroids and cell cycling in rice. *Plant J.* **70,** 398-408 (2012).

88. Aguilar-Martínez, J. A., Poza-Carrión, C. & Cubas, P. Arabidopsis *BRANCHED1* acts as an integrator of branching signals within axillary buds. *Plant Cell* **19,** 458-472 (2007).

89. Rouse, D., Mackay, P., Stirnberg, P., Estelle, M. & Leyser, O. Changes in auxin response from mutations in an AUX/IAA gene. *Science* **279,** 1371-1373 (1998).

90. Lazar, G. & Goodman, H. M. *MAX1*, a regulator of the flavonoid pathway, controls vegetative axillary bud outgrowth in Arabidopsis. *Proc. Natl. Acad. Sci. U. S. A.* **103,** 472-476 (2006).

91. Stirnberg, P., Furner, I. J. & Ottoline Leyser, H. MAX2 participates in an SCF complex which acts locally at the node to suppress shoot branching. *Plant J.* **50,**

80-94 (2007).

92. Booker, J. *et al*. MAX3/CCD7 is a carotenoid cleavage dioxygenase required for the synthesis of a novel plant signaling molecule. *Curr. Biol.* **14,** 1232-1238 (2004).

93. Sorefan, K. *et al*. *MAX4* and *RMS1* are orthologous dioxygenase-like genes that regulate shoot branching in Arabidopsis and pea. *Gene Dev.* **17,** 1469-1474 (2003).

94. Doebley, J., Stec, A. & Hubbard, L. The evolution of apical dominance in maize. *Nature* **386,** 485-488 (1997).

95. Hamiaux, C. *et al*. DAD2 is an α/β hydrolase likely to be involved in the perception of the plant branching hormone, strigolactone. *Curr. Biol.* **22,** 2032-2036 (2012).

96. Foo, E. *et al*. The branching gene *RAMOSUS1* mediates interactions among two novel signals and auxin in pea. *Plant Cell* **17,** 464-474 (2005).

97. Arite, T. *et al*. *d14*, a strigolactone-insensitive mutant of rice, shows an accelerated outgrowth of tillers. *Plant Cell Physiol.* **50,** 1416-1424 (2009).

98. Zou, J. *et al*. The rice *HIGH-TILLERING DWARF1* encoding an ortholog of Arabidopsis *MAX3* is required for negative regulation of the outgrowth of axillary buds. *Plant J.* **48,** 687-698 (2006).

99. Lin, H. *et al*. *DWARF27*, an iron-containing protein required for the biosynthesis of strigolactones, regulates rice tiller bud outgrowth. *Plant Cell* **21,** 1512-1525 (2009).

100. Jiang, L. *et al*. *DWARF 53* acts as a repressor of strigolactone signalling in rice.

Nature **504,** 401-405 (2013).

101. Zhou, F. *et al*. D14-SCFD3-dependent degradation of *D53* regulates strigolactone signalling. *Nature* **504,** 406–410 (2013).

102. Gao, Z. *et al*. *Dwarf 88*, a novel putative esterase gene affecting architecture of rice plant. *Plant Mol. Biol.* **71,** 265-276 (2009).

103. Liu, W. *et al*. Identification and characterization of *HTD2*: a novel gene negatively regulating tiller bud outgrowth in rice. *Planta* **230,** 649-658 (2009).

104. Takeda, T. *et al*. The *OsTB1* gene negatively regulates lateral branching in rice. *Plant J.* **33,** 513-520 (2003).

105. Cardoso, C. *et al*. Natural variation of rice strigolactone biosynthesis is associated with the deletion of two *MAX1* orthologs. *Proc. Natl. Acad. Sci. U. S. A.* **111,** 2379-2384 (2014).

106. Eloy, N. B. *et al*. The APC/C subunit 10 plays an essential role in cell proliferation during leaf development. *Plant J.* **68,** 351-363 (2011).

107. Zheng, B., Chen, X. & McCormick, S. The anaphase-promoting complex is a dual integrator that regulates both MicroRNA-mediated transcriptional regulation of cyclin B1 and degradation of Cyclin B1 during Arabidopsis male gametophyte development. *Plant Cell* **23,** 1033-1046 (2011).

108. Capron, A. *et al*. The Arabidopsis anaphase-promoting complex or cyclosome: molecular and genetic characterization of the APC2 subunit. *Plant Cell* **15,** 2370-2382 (2003).

109. Wang, Y. *et al*. The Arabidopsis APC4 subunit of the anaphase-promoting

complex/cyclosome (APC/C) is critical for both female gametogenesis and embryogenesis. *Plant J.* **69,** 227-240 (2012).

110. Kwee, H. S. & Sundaresan, V. The *NOMEGA* gene required for female gametophyte development encodes the putative *APC6/CDC16* component of the Anaphase Promoting Complex in Arabidopsis. *Plant J.* **36,** 853-866 (2003).

111. Waters, M. T., Brewer, P. B., Bussell, J. D., Smith, S. M. & Beveridge, C. A. The Arabidopsis ortholog of rice *DWARF27* acts upstream of *MAX1* in the control of plant development by strigolactones. *Plant Physiol.* **159,** 1073-1085 (2012).

112. Vinardell, J. M. *et al*. Endoreduplication mediated by the anaphase-promoting complex activator *CCS52A* is required for symbiotic cell differentiation in Medicago truncatula nodules. *Plant Cell* **15,** 2093-2105 (2003).

113. Kevei, Z. *et al*. Conserved *CDC20* cell cycle functions are carried out by two of the five isoforms in *Arabidopsis thaliana*. *PLoS ONE* **6,** e20618 (2011).

114. Blilou, I. *et al*. The Arabidopsis *HOBBIT* gene encodes a *CDC27* homolog that links the Plant Cell cycle to progression of cell differentiation. *Gene Dev.* **16,** 2566-2575 (2002).

115. Deng, Y. *et al*. Arabidopsis histidine kinase *CKI1* acts upstream of histidine phosphotransfer proteins to regulate female gametophyte development and vegetative growth. *Plant Cell* **22,** 1232-1248 (2010).

116. Day, I. S. & Reddy, A. Cloning of a family of cyclins from *Arabidopsis thaliana*. *Biochimica et Biophysica Acta (BBA)-Gene Structure and Expression* **1218,** 115-118 (1994).

117. Lee, J. *et al*. Cell cycle function of a rice B2-type cyclin interacting with a B-type cyclin-dependent kinase. *Plant J.* **34,** 417-425 (2003).

118. Cockcroft, C. E., den Boer, B. G., Healy, J. S. & Murray, J. A. *Cyclin D* control of growth rate in plants. *Nature* **405,** 575-579 (2000).

119. Iwata, E. *et al*. *GIGAS CELL1*, a novel negative regulator of the anaphase-promoting complex/cyclosome, is required for proper mitotic progression and cell fate determination in Arabidopsis. *Plant Cell* **23,** 4382-4393 (2011).

120. Heyman, J. *et al*. Arabidopsis *ULTRAVIOLET-B-INSENSITIVE4* maintains cell division activity by temporal inhibition of the anaphase-promoting complex/cyclosome. *Plant Cell* **23,** 4394-4410 (2011).

121. Xu, C. *et al*. Degradation of *MONOCULM 1* by APC/CTAD1 regulates rice tillering. *Nature Commun.* **3,** 750 (2012).

122. Giraudat, J. *et al*. Isolation of the Arabidopsis *ABI3* gene by positional cloning. *Plant Cell* **4,** 1251-1261 (1992).

123. Nole-Wilson, S., Tranby, T. L. & Krizek, B. A. *AINTEGUMENTA-like* (*AIL*) genes are expressed in young tissues and may specify meristematic or division-competent states. *Plant Mol. Biol.* **57,** 613-628 (2005).

124. Kim, G. T. *et al*. The *ANGUSTIFOLIA* gene of Arabidopsis, a plant *CtBP* gene, regulates leaf-cell expansion, the arrangement of cortical microtubules in leaf cells and expression of a gene involved in cell-wall formation. *EMBO J.* **21,** 1267-1279 (2002).

125. Elliott, R. C. *et al*. *AINTEGUMENTA*, an *APETALA2-like* gene of Arabidopsis with pleiotropic roles in ovule development and floral organ growth. *Plant Cell* **8,** 155-168 (1996).

126. Purugganan, M. D. & Suddith, J. I. Molecular population genetics of the Arabidopsis *CAULIFLOWER* regulatory gene: nonneutral evolution and naturally occurring variation in floral homeotic function. *Proc. Natl. Acad. Sci. U. S. A.* **95,** 8130-8134 (1998).

127. Hibara, K. I., Takada, S. & Tasaka, M. *CUC1* gene activates the expression of SAM-related genes to induce adventitious shoot formation. *Plant J.* **36,** 687-696 (2003).

128. Kim, J. H. & Kende, H. A transcriptional coactivator, *AtGIF1*, is involved in regulating leaf growth and morphology in Arabidopsis. *Proc. Natl. Acad. Sci. U. S. A.* **101,** 13374-13379 (2004).

129. Kim, J. H., Choi, D. & Kende, H. The *AtGRF* family of putative transcription factors is involved in leaf and cotyledon growth in Arabidopsis. *Plant J.* **36,** 94-104 (2003).

130. Palatnik, J. F. *et al*. Control of leaf morphogenesis by microRNAs. *Nature* **425,** 257-263 (2003).

131. Greb, T. *et al*. Molecular analysis of the *LATERAL SUPPRESSOR* gene in Arabidopsis reveals a conserved control mechanism for axillary meristem formation. *Gene Dev.* **17,** 1175-1187 (2003).

132. Weigel, D., Alvarez, J., Smyth, D. R., Yanofsky, M. F. & Meyerowitz, E. M.

LEAFY controls floral meristem identity in Arabidopsis. *Cell* **69,** 843-859 (1992).

133. Keller, T., Abbott, J., Moritz, T. & Doerner, P. Arabidopsis *REGULATOR OF AXILLARY MERISTEMS1* controls a leaf axil stem cell niche and modulates vegetative development. *Plant Cell* **18,** 598-611 (2006).

134. Otsuga, D., DeGuzman, B., Prigge, M. J., Drews, G. N. & Clark, S. E. *REVOLUTA* regulates meristem initiation at lateral positions. *Plant J.* **25,** 223-236 (2001).

135. Shannon, S. & Meeks-Wagner, D. R. A mutation in the Arabidopsis *TFL1* gene affects inflorescence meristem development. *Plant Cell* **3,** 877-892 (1991).

136. Gallavotti, A. *et al*. The role of *barren stalk1* in the architecture of maize. *Nature* **432,** 630-635 (2004).

137. Souer, E., van Houwelingen, A., Kloos, D., Mol, J. & Koes, R. *The No Apical Meristem* gene of petunia is required for pattern formation in embryos and flowers and is expressed at meristem and primordia boundaries. *Cell* **85,** 159-170 (1996).

138. Weller, J. L., Reid, J. B., Taylor, S. A. & Murfet, I. C. The genetic control of flowering in pea. *Trends Plant Sci.* **2,** 412-418 (1997).

139. Lu, Z. *et al*. Genome-wide binding analysis of the transcription activator *IDEAL PLANT ARCHITECTURE1* reveals a complex network regulating rice plant architecture. *Plant Cell* **25,** 3743-3759 (2013).

140. Guo, S. *et al*. The interaction between *OsMADS57* and *OsTB1* modulates rice tillering via *DWARF14*. *Nature Commun.* **4,** 1566 (2013).

141. Li, X. *et al*. Control of tillering in rice. *Nature* **422,** 618-621 (2003).

142. Komatsu, M., Maekawa, M., Shimamoto, K. & Kyozuka, J. The *LAX1* and

FRIZZY PANICLE 2 genes determine the inflorescence architecture of rice by controlling rachis-branch and spikelet development. *Dev. Biol.* **231,** 364-373 (2001).

143. Kosugi, S. & Ohashi, Y. *PCF1* and *PCF2* specifically bind to cis elements in the rice proliferating cell nuclear antigen gene. *Plant Cell* **9,** 1607-1619 (1997).

144. Jin, J. *et al*. Genetic control of rice plant architecture under domestication. *Nat. Genet.* **40,** 1365-1369 (2008).

145. Schumacher, K., Schmitt, T., Rossberg, M., Schmitz, G. & Theres, K. The *Lateral suppressor* (*Ls*) gene of tomato encodes a new member of the VHIID protein family. *Proc. Natl. Acad. Sci. U. S. A.* **96,** 290-295 (1999).

146. Pnueli, L. *et al*. The *SELF-PRUNING* gene of tomato regulates vegetative to reproductive switching of sympodial meristems and is the ortholog of *CEN* and *TFL1*. *Development* **125,** 1979-1989 (1998).

147. Mizukami, Y. & Ma, H. Ectopic expression of the floral homeotic gene *AGAMOUS* in transgenic Arabidopsis plants alters floral organ identity. *Cell* **71,** 119-131 (1992).

148. Peña, L. *et al*. Constitutive expression of Arabidopsis *LEAFY* or *APETALA1* genes in citrus reduces their generation time. *Nat. Biotechnol.* **19,** 263-267 (2001).

149. Riechmann, J. L., Krizek, B. A. & Meyerowitz, E. M. Dimerization specificity of Arabidopsis MADS domain homeotic proteins APETALA1, APETALA3, PISTILLATA, and AGAMOUS. *Proc. Natl. Acad. Sci. U. S. A.* **93,** 4793-4798 (1996).

150. Zhao, D., Yu, Q., Chen, M. & Ma, H. The *ASK1* gene regulates B function gene expression in cooperation with *UFO* and *LEAFY* in Arabidopsis. *Development* **128,** 2735-2746 (2001).

151. Williams, L. & Fletcher, J. C. Stem cell regulation in the Arabidopsis shoot apical meristem. *Curr. Opin. Plant Biol.* **8,** 582-586 (2005).

152. Aubert, D. *et al*. *EMF1*, a novel protein involved in the control of shoot architecture and flowering in Arabidopsis. *Plant Cell* **13,** 1865-1875 (2001).

153. Masle, J., Gilmore, S. R. & Farquhar, G. D. The *ERECTA* gene regulates plant transpiration efficiency in Arabidopsis. *Nature* **436,** 866-870 (2005).

154. Allen, G. J., Murata, Y., Chu, S. P., Nafisi, M. & Schroeder, J. I. Hypersensitivity of abscisic acid-induced cytosolic Calcium increases in the Arabidopsis farnesyltransferase mutant *era1-2*. *Plant Cell* **14,** 1649-1662 (2002).

155. Lin, W., Shuai, B. & Springer, P. S. The Arabidopsis LATERAL ORGAN BOUNDARIES–domain gene *ASYMMETRIC LEAVES2* functions in the repression of *KNOX* gene expression and in adaxial-abaxial patterning. *Plant Cell* **15,** 2241-2252 (2003).

156. McConnell, J. R. *et al*. Role of *PHABULOSA* and *PHAVOLUTA* in determining radial patterning in shoots. *Nature* **411,** 709-713 (2001).

157. McConnell, J. R. & Barton, M. K. Effect of mutations in the *PINHEAD* gene of Arabidopsis on the formation of shoot apical meristems. *Dev. Genet.* **16,** 358-366 (1995).

158. Ståldal, V., Sohlberg, J. J., Eklund, D. M., Ljung, K. & Sundberg, E. Auxin can act

independently of *CRC*, *LUG*, *SEU*, *SPT* and *STY1* in style development but not apical-basal patterning of the Arabidopsis gynoecium. *New Phytol.* **180,** 798-808 (2008).

159. Schwarz, S., Grande, A. V., Bujdoso, N., Saedler, H. & Huijser, P. The microRNA regulated SBP-box genes *SPL9* and *SPL15* control shoot maturation in Arabidopsis. *Plant Mol. Biol.* **67,** 183-195 (2008).

160. Jun, L., Saiki, R., Tatsumi, K., Nakagawa, T. & Kawamukai, M. Identification and subcellular localization of two solanesyl diphosphate synthases from *Arabidopsis thaliana*. *Plant Cell Physiol.* **45,** 1882-1888 (2004).

161. Wu, X., Dabi, T. & Weigel, D. Requirement of homeobox gene *STIMPY WOX9* for Arabidopsis meristem growth and maintenance. *Curr. Biol.* **15,** 436-440 (2005).

162. Kwon, C. S., Chen, C. & Wagner, D. *WUSCHEL* is a primary target for transcriptional regulation by *SPLAYED* in dynamic control of stem cell fate in Arabidopsis. *Gene Dev.* **19,** 992-1003 (2005).

163. Taguchi-Shiobara, F., Yuan, Z., Hake, S. & Jackson, D. The *fasciated ear2* gene encodes a leucine-rich repeat receptor-like protein that regulates shoot meristem proliferation in maize. *Gene Dev.* **15,** 2755-2766 (2001).

164. Vollbrecht, E., Springer, P. S., Goh, L., Buckler IV, E. S. & Martienssen, R. Architecture of floral branch systems in maize and related grasses. *Nature* **436,** 1119-1126 (2005).

165. Bortiri, E. *et al*. *ramosa2* encodes a LATERAL ORGAN BOUNDARY domain protein that determines the fate of stem cells in branch meristems of maize. *Plant*

Cell **18,** 574-585 (2006).

166. Satoh-Nagasawa, N., Nagasawa, N., Malcomber, S., Sakai, H. & Jackson, D. A trehalose metabolic enzyme controls inflorescence architecture in maize. *Nature* **441,** 227-230 (2006).
167. Bommert, P. *et al*. *thick tassel dwarf1* encodes a putative maize ortholog of the Arabidopsis *CLAVATA1* leucine-rich repeat receptor-like kinase. *Development* **132,** 1235-1245 (2005).
168. Ikeda, K., Ito, M., Nagasawa, N., Kyozuka, J. & Nagato, Y. Rice *ABERRANT PANICLE ORGANIZATION 1*, encoding an F-box protein, regulates meristem fate. *Plant J.* **51,** 1030-1040 (2007).
169. Ikeda-Kawakatsu, K., Maekawa, M., Izawa, T., Itoh, J. I. & Nagato, Y. *ABERRANT PANICLE ORGANIZATION 2/RFL*, the rice ortholog of Arabidopsis *LEAFY,* suppresses the transition from inflorescence meristem to floral meristem through interaction with *APO1*. *Plant J.* **69,** 168-180 (2012).
170. Huang, X. *et al*. Natural variation at the *DEP1* locus enhances grain yield in rice. *Nat. Genet.* **41,** 494-497 (2009).
171. Suzaki, T. *et al*. The gene *FLORAL ORGAN NUMBER1* regulates floral meristem size in rice and encodes a leucine-rich repeat receptor kinase orthologous to Arabidopsis *CLAVATA1*. *Development* **131,** 5649-5657 (2004).
172. Chu, H. *et al*. The *FLORAL ORGAN NUMBER4* gene encoding a putative ortholog of Arabidopsis *CLAVATA3* regulates apical meristem size in rice. *Plant Physiol.* **142,** 1039-1052 (2006).

173. Komatsu, M., Chujo, A., Nagato, Y., Shimamoto, K. & Kyozuka, J. *FRIZZY PANICLE* is required to prevent the formation of axillary meristems and to establish floral meristem identity in rice spikelets. *Development* **130,** 3841-3850 (2003).

174. Jiao, Y. *et al*. Regulation of *OsSPL14* by *OsmiR156* defines ideal plant architecture in rice. *Nat. Genet.* **42,** 541-544 (2010).

175. Dong, Z. *et al*. Maize *LAZY1* mediates shoot gravitropism and inflorescence development through regulating auxin transport, auxin signaling, and light response. *Plant Physiol.* **163,** 1306-1322 (2013).

176. Wu, X., Tang, D., Li, M., Wang, K. & Cheng, Z. *Loose plant architecture1,* an INDETERMINATE DOMAIN protein involved in shoot gravitropism, regulates plant architecture in rice. *Plant Physiol.* **161,** 317-329 (2013).

177. Morita, R., Sato, Y., Masuda, Y., Nishimura, M. & Kusaba, M. Defect in non-yellow coloring 3, an α/β hydrolase-fold family protein, causes a stay-green phenotype during leaf senescence in rice. *Plant J.* **59,** 940-952 (2009).

178. Kim, S. R. *et al*. Rice *OGR1* encodes a pentatricopeptide repeat-DYW protein and is essential for RNA editing in mitochondria. *Plant J.* **59,** 738-749 (2009).

179. Sato, Y. *et al*. A rice homeobox gene, *OSH1,* is expressed before organ differentiation in a specific region during early embryogenesis. *Proc. Natl. Acad. Sci. U. S. A.* **93,** 8117-8122 (1996).

180. Miura, K. *et al*. *OsSPL14* promotes panicle branching and higher grain productivity in rice. *Nat. Genet.* **42,** 545-549 (2010).

181. Miyata, M. *et al*. Fine scale and physical mapping of *Spk (t)* controlling spreading stub in rice. *Breeding Sci.* **55,** 237-239 (2005).

182. Yu, B. *et al*. *TAC1*, a major quantitative trait locus controlling tiller angle in rice. *Plant J.* **52,** 891-898 (2007).

183. Yoshida, A. *et al*. *TAWAWA1*, a regulator of rice inflorescence architecture, functions through the suppression of meristem phase transition. *Proc. Natl. Acad. Sci. U. S. A.* **110,** 767-772 (2013).

Supplementary Table S7. Physical positions of the SSR and SNP markers used in the HJ-DH population, and the fragments of homoeologous exchange identified in the genetic linkage map.

Supplementary Table S8. Homologous genes for plant architecture in *B. napus*, and their positions in the *B. napus* genetic linkage map.

Supplementary Table S9. Predicted candidate genes for 105 unique QTLs.

Supplementary Table S10. Three pleiotropic QTLs of PY major QTLs, and their candidate genes.

Supplementary Table S11. Detailed information of epistatic interaction loci-pairs for plant yield identified in four environments of the HJ-DH population.

Supplementary Table S12. Detailed information for 183 interaction loci-pairs that were repeatedly detected in two or more environments and their corresponding QTLs.

Supplementary Table S13. Conserved blocks that were located or overlapped on the epistatic interaction loci-pairs for plant yield identified in four environments.

(Because of the large tables, the Supplementary Table S7-S13 were in the Supplementary Dataset)

Supplementary Table S14. Primers used for cloning of the genomic DNA fragments of *Bna.A02.CLV2* (*BnaA02g12070D*), *Bna.C07.AHK4* (*BnaC07g21260D*), and *Bna.A09.SLY2* (*BnaA09g02870D*) gene in *Brassica napus*.

**Gene**	**Primer**	**Sequence (5'—3')**
Bna.A02.CLV2 (*BnaA02g12070D*)	CLV2_F	ATGGTGAAGACTGCATATCT
	CLV2_R	TTAACCTTTGGTCTGGAGAA
Bna.C07.AHK4 (*BnaC07g21260D*)	AHK4_F	ATGTTCAATCCTTTCACAAC
	AHK4_R	TCAACTTTGGCTACATGACG
Bna.A09.SLY2 (*BnaA09g02870D*)	SLY2_F	ATGTCGAAGAAACGAATTGG
	SLY2_R	TCAGACGACGTTGACGGGCA

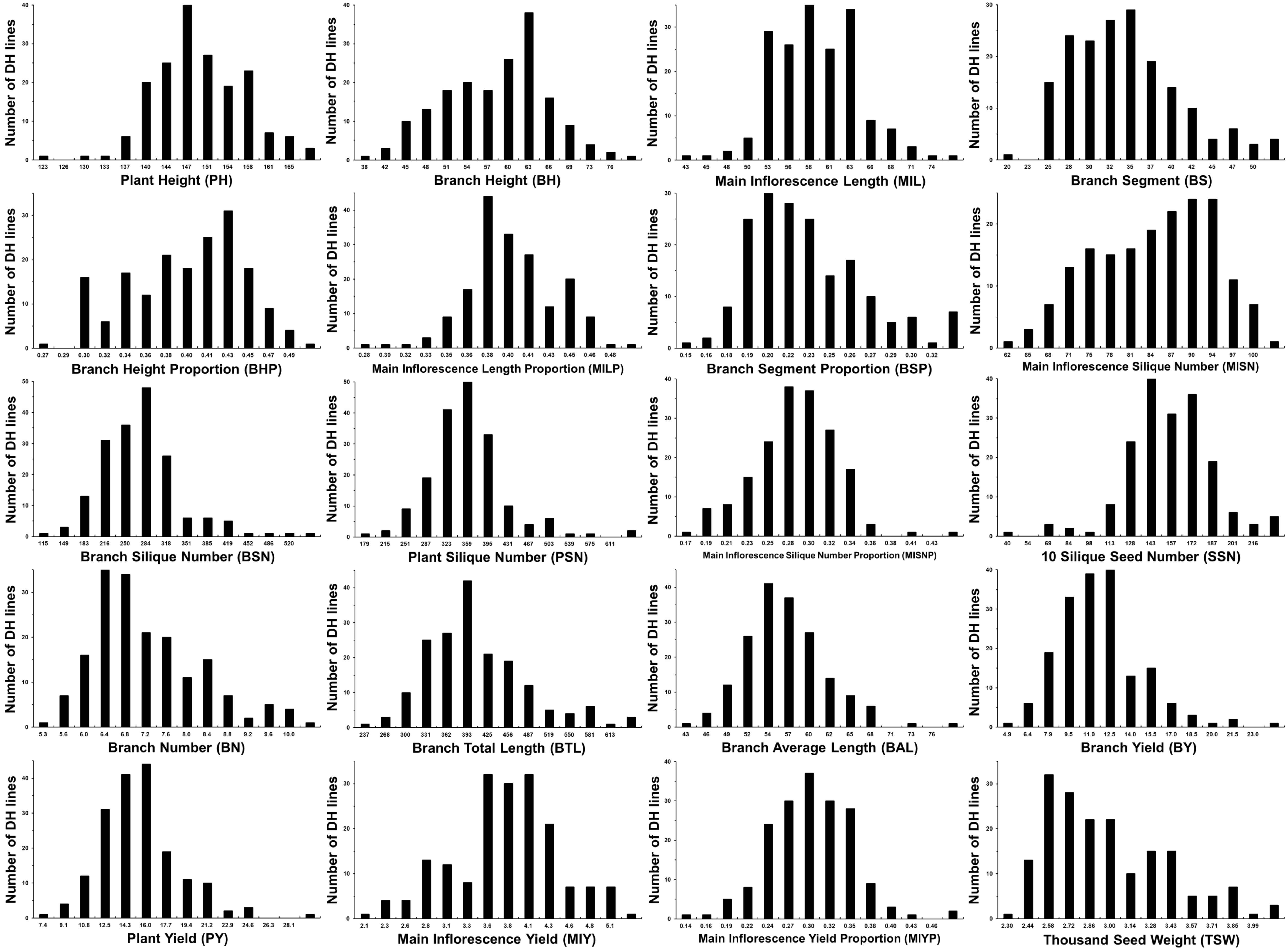

Supplementary Figure S1. Distribution of the 20 plant architecture and yield traits in the HJ_DH population derived from the cross of Hua_5 and J7005 in the year of 2009-2010 in Wuhan (WH09).

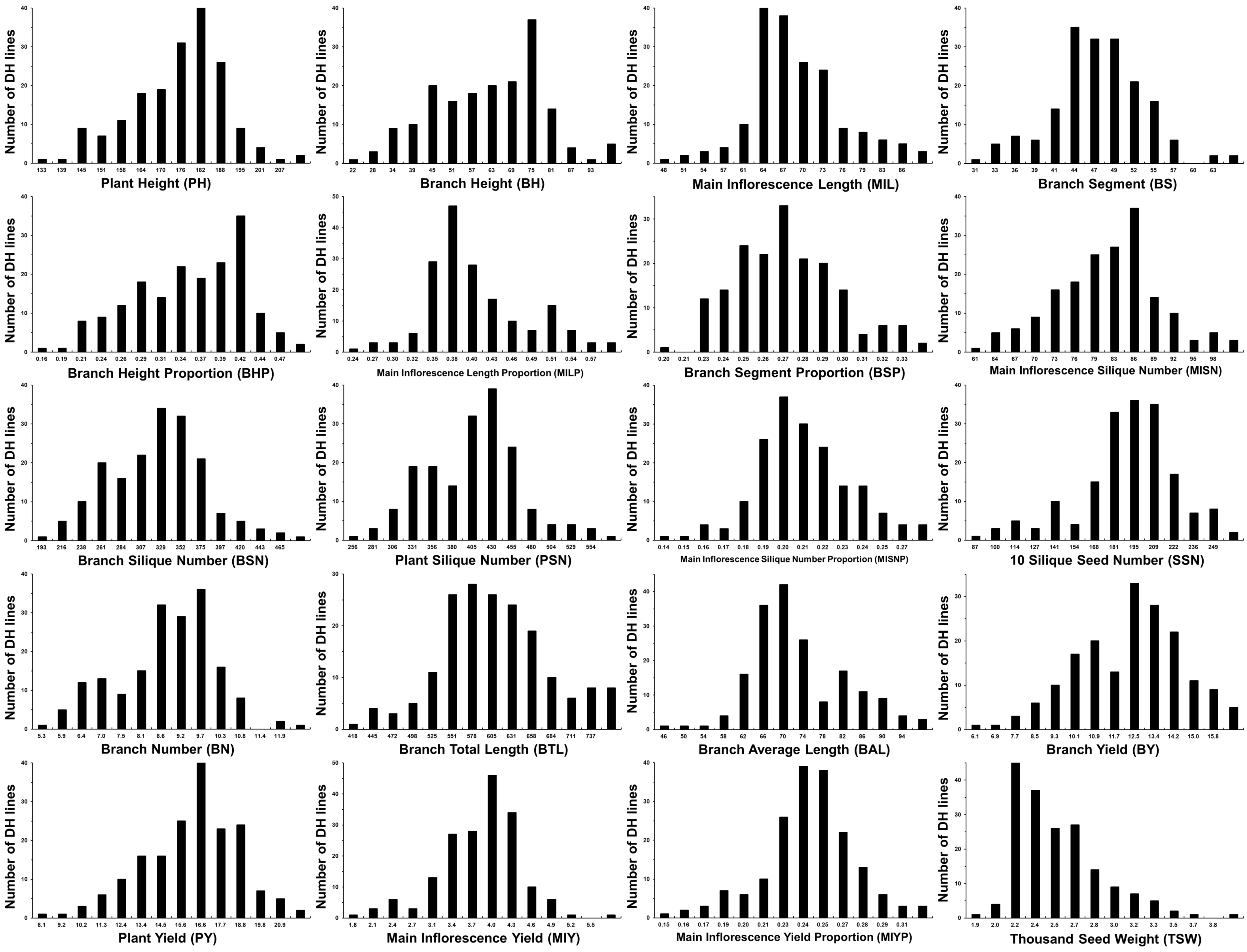

Supplementary Figure S2. Distribution of the 20 plant architecture and yield traits in the HJ_DH population derived from the cross of Hua_5 and J7005 in the year of 2010-2011 in Wuhan (WH10).

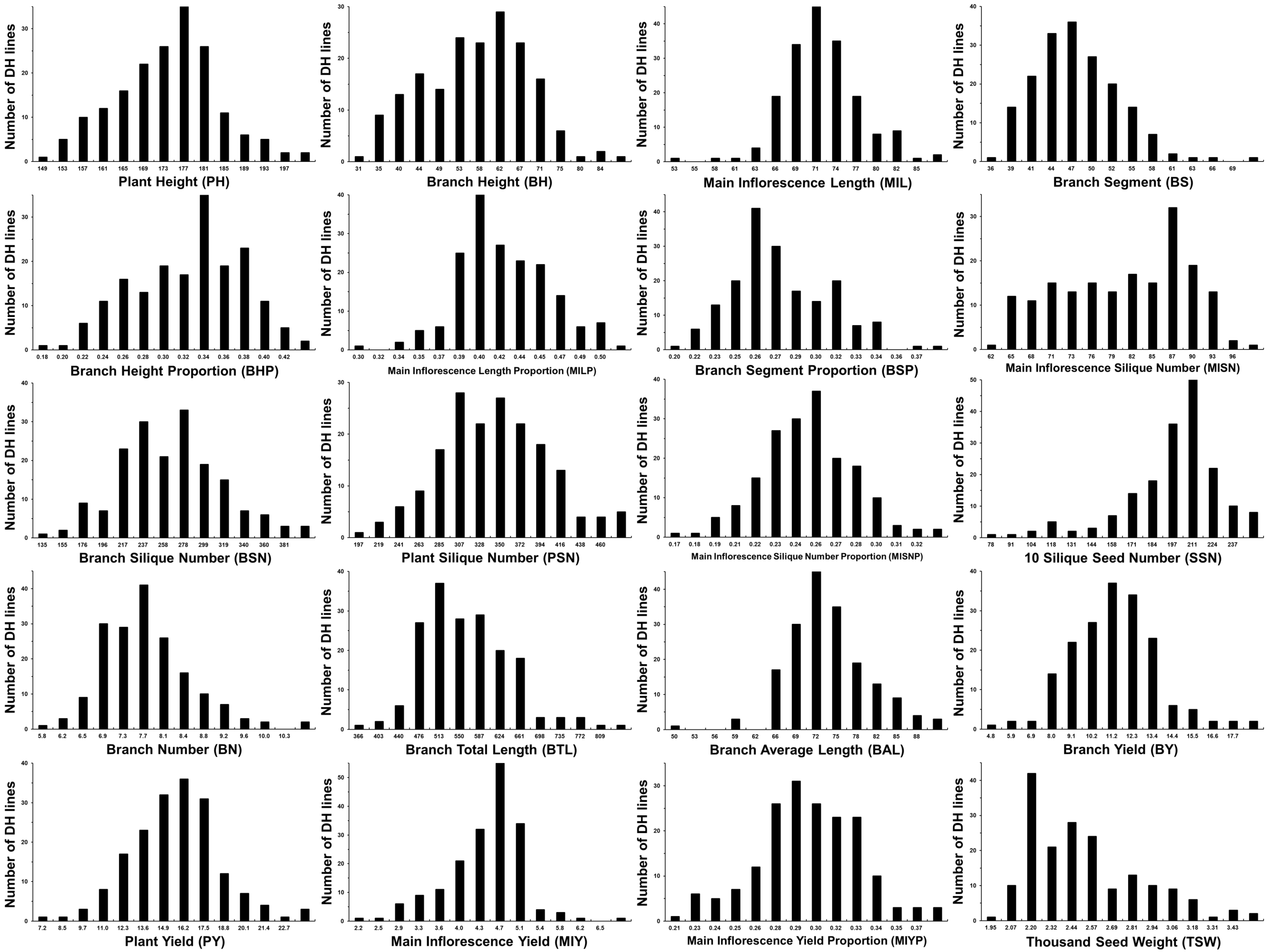

Supplementary Figure S3. Distribution of the 20 plant architecture and yield traits in the HJ_DH population derived from the cross of Hua_5 and J7005 in the year of 2010-2011 in Huanggang (HG10).

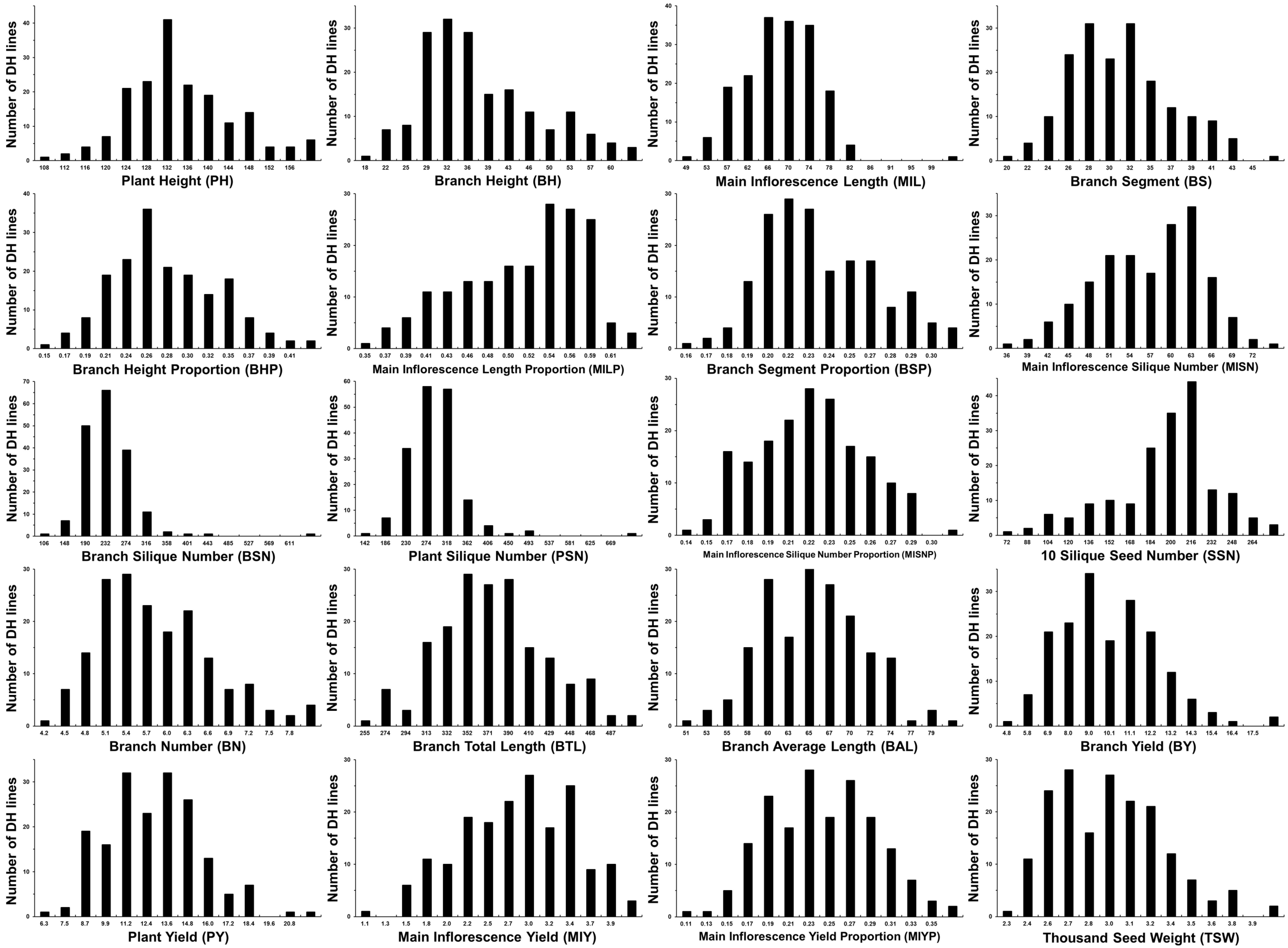

Supplementary Figure S4. Distribution of the 20 plant architecture and yield traits in the HJ_DH population derived from the cross of Hua_5 and J7005 in the year of 2011 in Gansu (GS11).

```
                             10        20        30        40        50        60        70        80        90       100
                    ....|....|....|....|....|....|....|....|....|....|....|....|....|....|....|....|....|....|....|....|
Ref(BnaA02g12070D)  ATGGTGAAGACTGCATATCTTACCCTCTTCTTCT------TCCTCTTCTCTCCTTCCTTGCTTCTAGCTCAATCACAGCCTCCAGAGATTGACCCACAAG
                     M  V  K  T  A  Y  L  T  L  F  F        F  L  F  S  P  S  L  L  L  A  Q  S  Q  P  P  E  I  D  P  Q
Hua_5               ATGGTGAAGACTGCATATCTTACCCTCTTCTTCT------TCCTCTTCTCTCCTTCCTTGCTTCTAGCTCAATCACAGCCTCCAGAGATTGACCCACAAG
                     M  V  K  T  A  Y  L  T  L  F  F        F  L  F  S  P  S  L  L  L  A  Q  S  Q  P  P  E  I  D  P  Q
J7005               ATGGTGAAGACTGCATATCTTACCCTCTTCTTCT------TCCTCTTCTCTCCTTCCTTGCTTCTAGCTCAATCACAGCCTCCAGAGATTGACCCACAAG
                     M  V  K  T  A  Y  L  T  L  F  F        F  L  F  S  P  S  L  L  L  A  Q  S  Q  P  P  E  I  D  P  Q
AtCLV2              ATGATAAAGATTGCAGATTTCACTCTCTTCTTCTTTATCTTCGTCTTCTCTCCCTCGTTGCCTCTAGCTCAATCTCAGCTTCCCGATCTCGACCCACAAG
                     M  I  K  I  A  D  F  T  L  F  F  F  I  F  V  F  S  P  S  L  P  L  A  Q  S  Q  L  P  D  L  D  P  Q
Clustal Consensus   *** * **** **** ** * ** **********      ** ********** ** **** ************ **** *** **  * **********

                            110       120       130       140       150       160       170       180       190       200
                    ....|....|....|....|....|....|....|....|....|....|....|....|....|....|....|....|....|....|....|
Ref(BnaA02g12070D)  ACAAAGCCTCGCTATTGATACTCCGCGTTTCGATTCACGACCCCAATCGAAGCTTATCGACATGGTACGGCTCATCATGTTCCAACTGGACAGGTCTAGC
                    D  K  A  S  L  L  I  L  R  V  S  I  H  D  P  N  R  S  L  S  T  W  Y  G  S  S  C  S  N  W  T  G  L  A
Hua_5               ACAAAGCCTCGCTATTGATACTCCGCGTTTCGATTCACGACCCCAATCGAAGCTTATCGACATGGTACGGCTCATCATGTTCCAACTGGACAGGTCTAGC
                    D  K  A  S  L  L  I  L  R  V  S  I  H  D  P  N  R  S  L  S  T  W  Y  G  S  S  C  S  N  W  T  G  L  A
J7005               ACAAAGCCTCGCTATTGATACTCCGCGTTTCGATTCACGACCCCAATCGAAGCTTATCGACATGGTACGGCTCATCATGTTCCAACTGGACAGGTCTAGC
                    D  K  A  S  L  L  I  L  R  V  S  I  H  D  P  N  R  S  L  S  T  W  Y  G  S  S  C  S  N  W  T  G  L  A
AtCLV2              ACAAAGCTTCTTTATTGATATTCAGAGTTTCGATTCACGACCTAAATCGAAGCTTATCGACATGGTACGGCTCATCGTGTTCTAACTGGACAGGTCTAGC
                    D  K  A  S  L  L  I  F  R  V  S  I  H  D  L  N  R  S  L  S  T  W  Y  G  S  S  C  S  N  W  T  G  L  A
Clustal Consensus   ******* **  ******** ** * ****************  ******************************** ***** *****************

                            210       220       230       240       250       260       270       280       290       300
                    ....|....|....|....|....|....|....|....|....|....|....|....|....|....|....|....|....|....|....|
Ref(BnaA02g12070D)  TTGTCAGAATCCGACCGGAAAAGTCGTCTCCCTCACTATACCCGCTTCGAATTTGTCCGGCCAGATTCACCCGTCTCTCTGTAAGCTCACTTCACTTCAA
                      C  Q  N  P  T  G  K  V  V  S  L  T  I  P  A  S  N  L  S  G  Q  I  H  P  S  L  C  K  L  T  S  L  Q
Hua_5               TTGTCAGAATCCGACCGGAAAAGTCGTCTCCCTCACTATACCCGCTTCGAATTTGTCCGGCCAGATTCACCCGTCTCTCTGTAAGCTCACTTCACTTCAA
                      C  Q  N  P  T  G  K  V  V  S  L  T  I  P  A  S  N  L  S  G  Q  I  H  P  S  L  C  K  L  T  S  L  Q
J7005               TTGTCAGAATCCGACCGGAAAAGTCGTCTCCCTCACTATACCCGCTTCGAATTTGTCCGGCCAGATTCACCCGTCTCTCTGTAAGCTCACTTCACTTCAA
                      C  Q  N  P  T  G  K  V  V  S  L  T  I  P  A  S  N  L  S  G  Q  I  H  P  S  L  C  K  L  T  S  L  Q
AtCLV2              TTGTCAGAATCCGACCGGAAAAGTTCTCTCCCTCACTTTATCCGGTCTGAATCTCTCCAGTCAGATTCACCCATCTCTTTGTAAGCTCTCTTCTCTTCAA
                      C  Q  N  P  T  G  K  V  L  S  L  T  L  S  G  L  N  L  S  S  Q  I  H  P  S  L  C  K  L  S  S  L  Q
Clustal Consensus   ************************  *********** ** *** *  **** * *** * *********** ***** ********* **** ******

                            310       320       330       340       350       360       370       380       390       400
                    ....|....|....|....|....|....|....|....|....|....|....|....|....|....|....|....|....|....|....|
Ref(BnaA02g12070D)  ATCTTGGACCTTTCCGGAAACAATTTCTCCGGAAACATCCCCGCTTGCTTCGGCGCGCTGCGTGATCTCAGAACACTCAATCTTAGCCGGAACGGTTTGG
                     I  L  D  L  S  G  N  N  F  S  G  N  I  P  A  C  F  G  A  L  R  D  L  R  T  L  N  L  S  R  N  G  L
Hua_5               ATCTTGGACCTTTCCGGAAACAATTTCTCCGGAAACATCCCCGCTTGCTTCGGCGCGCTGCGTGATCTCAGAACACTCAATCTTAGCCGGAACGGTTTGG
                     I  L  D  L  S  G  N  N  F  S  G  N  I  P  A  C  F  G  A  L  R  D  L  R  T  L  N  L  S  R  N  G  L
J7005               ATCTTGGACCTTTCCGGAAACAATTTCTCCGGAAACATCCCCGCTTGCTTCGGCGCGCTGCGTGATCTCAGAACACTCAATCTTAGCCGGAACGGTTTGG
                     I  L  D  L  S  G  N  N  F  S  G  N  I  P  A  C  F  G  A  L  R  D  L  R  T  L  N  L  S  R  N  G  L
AtCLV2              AGCTTGGACCTTTCTCACAACAACTTCTCTGGAAACATTCCTTCTTGCTTCGGTTCGTTGCGTAATCTTAGGACACTTAATCTTAGTCGGAATAGATTCG
                     S  L  D  L  S  H  N  N  F  S  G  N  I  P  S  C  F  G  S  L  R  N  L  R  T  L  N  L  S  R  N  R  F
Clustal Consensus   * ************    ***** ***** ******** **  ********** ** ***** **** ** ***** ******** *****  * ** *

                            410       420       430       440       450       460       470       480       490       500
                    ....|....|....|....|....|....|....|....|....|....|....|....|....|....|....|....|....|....|....|
Ref(BnaA02g12070D)  TTGGTTCGGTTCCCGGAACGTTCGTTAACCTTAAGGAGCTTAGAGAATTAGTTCTAAGCGAGAATAGAGGTTTAAGAGGATCGGTTCCTCTCTGGTTTGG
                    V  G  S  V  P  G  T  F  V  N  L  K  E  L  R  E  L  V  L  S  E  N  R  G  L  R  G  S  V  P  L  W  F  G
Hua_5               TTGGTTCGGTTCCCGGAACGTTCGTTAACCTTAAGGAGCTTAGAGAATTAGTTCTAAGCGAGAATAGAGGTTTAAGAGGATCGGTTCCTCTCTGGTTTGG
                    V  G  S  V  P  G  T  F  V  N  L  K  E  L  R  E  L  V  L  S  E  N  R  G  L  R  G  S  V  P  L  W  F  G
J7005               TTGGTTCGGTTCCCGGAACGTTCGTTAACCTTAAGGAGCTTAGAGAATTAGTTCTAAGCGAGAATAGAGGTTTAAGAGGATCGGTTCCTCTCTGGTTTGG
                    V  G  S  V  P  G  T  F  V  N  L  K  E  L  R  E  L  V  L  S  E  N  R  G  L  R  G  S  V  P  L  W  F  G
AtCLV2              TTGGTTCGATACCCGCCACATTTGTAAGTCTTAAGGAGCTTAGAGAAGTTGTTCTAAGTGAGAATAGAGATTTAGGCGGTGTGGTCCCTCATTGGTTTGG
                    V  G  S  I  P  A  T  F  V  S  L  K  E  L  R  E  V  V  L  S  E  N  R  D  L  G  G  V  V  P  H  W  F  G
Clustal Consensus   ******** * ****  ** ** ** *  ****************** * ******** ********** **** * **   *** ****  ********

                            510       520       530       540       550       560       570       580       590       600
                    ....|....|....|....|....|....|....|....|....|....|....|....|....|....|....|....|....|....|....|
Ref(BnaA02g12070D)  TGATTT---GATGAATCTCGAGAGAATTGATCTTAGCTTCTGCTTGTTTAGTGGAGAGATACCTGAAACCTTGCTTTATTTAAAATCTCTAAAGGTTTTG
                      D  L     M  N  L  E  R  I  D  L  S  F  C  L  F  S  G  E  I  P  E  T  L  L  Y  L  K  S  L  K  V  L
Hua_5               TGATTT---GATGAATCTCGAGAGAATTGATCTTAGCTTCTGCTTGTTTAGTGGAGAGATACCTGAAACCTTGCTTTATTTAAAATCTCTAAAGGTTTTG
                      D  L     M  N  L  E  R  I  D  L  S  F  C  L  F  S  G  E  I  P  E  T  L  L  Y  L  K  S  L  K  V  L
J7005               TGATTT---GATGAATCTCGAGAGAATTGATCTTAGCTTCTGCTTGTTTAGTGGAGAGATACCTGAAACCTTGCTTTATTTAAAATCTCTAAAGGTTTTG
                      D  L     M  N  L  E  R  I  D  L  S  F  C  L  F  S  G  E  I  P  E  T  L  L  Y  L  K  S  L  K  V  L
AtCLV2              TAATTTCTCAATGAATCTTGAGAGAGTTGATTTTAGTTTCTGCTCGTTTGTTGGAGAACTACCTGAGAGTTTACTGTATTTGAAGTCTCTCAAGTACTTG
                      N  F  S  M  N  L  E  R  V  D  F  S  F  C  S  F  V  G  E  L  P  E  S  L  L  Y  L  K  S  L  K  Y  L
Clustal Consensus   * ****    ******** ****** ***** **** ******* ****  ******  ******* *  ** ** ***** ** ***** ***   ***
```

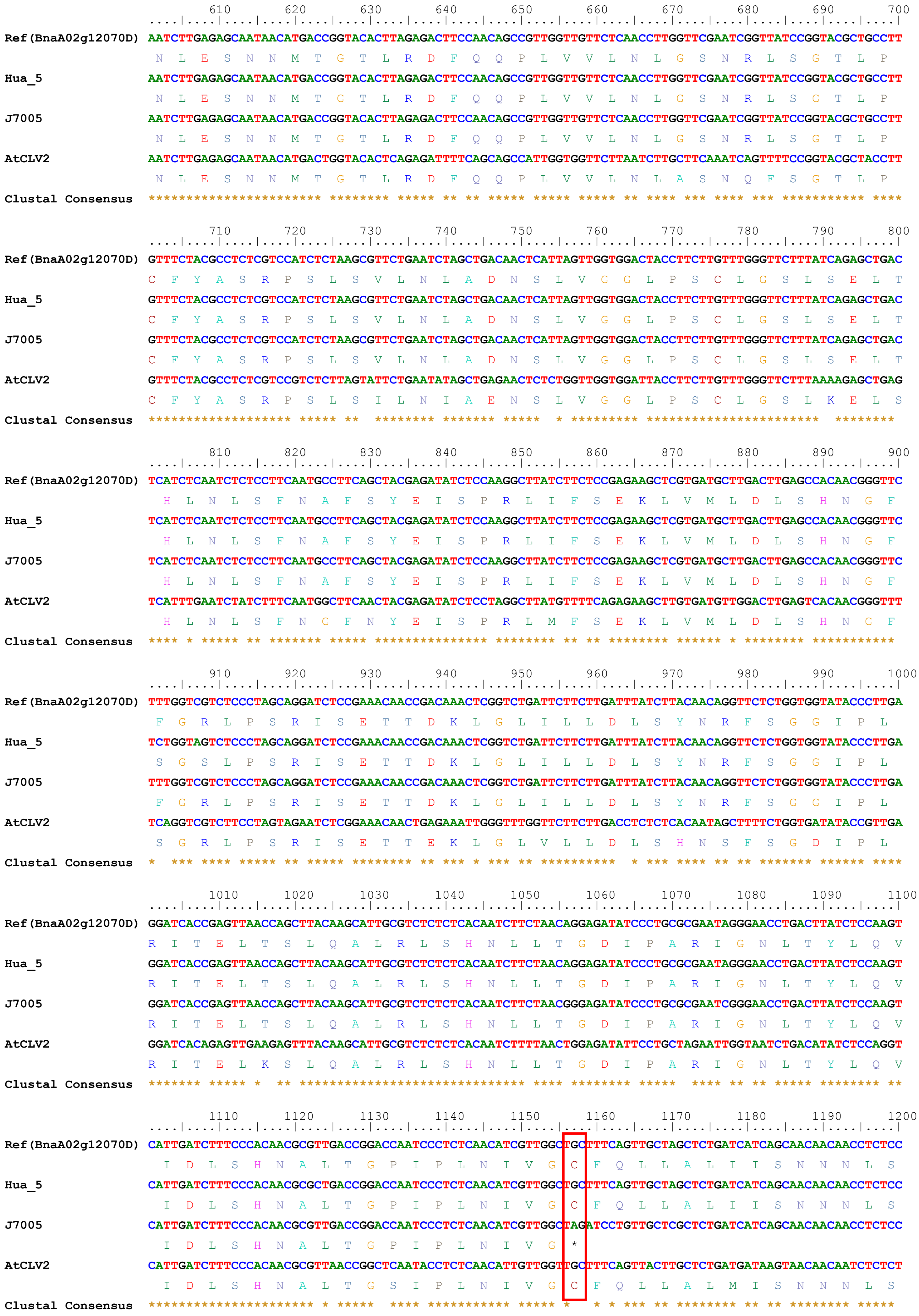

610 620 630 640 650 660 670 680 690 700
Ref(BnaA02g12070D)
Hua_5
J7005
AtCLV2
Clustal Consensus
710 720 730 740 750 760 770 780 790 800
810 820 830 840 850 860 870 880 890 900
910 920 930 940 950 960 970 980 990 1000
1010 1020 1030 1040 1050 1060 1070 1080 1090 1100
1110 1120 1130 1140 1150 1160 1170 1180 1190 1200

```
                          1210      1220      1230      1240      1250      1260      1270      1280      1290      1300
                    ....|....|....|....|....|....|....|....|....|....|....|....|....|....|....|....|....|....|....|....|
Ref(BnaA02g12070D)  GGCGAAATCCAGCCGGAGCTCGACGCGTTGGACAGCCTGAAGATACTTGACATAAGCAGCAACAGGATCTCTGGCGAGATCCCGCTGACTCTAGCCGGCC
                     G  E  I  Q  P  E  L  D  A  L  D  S  L  K  I  L  D  I  S  S  N  R  I  S  G  E  I  P  L  T  L  A  G
Hua_5               GGCGAAATCCAGCCGGAGCTCGACGCGTTGGACAGCCTGAAGATACTTGACATAAGCAGCAACAGGATCTCTGGCGAGATCCCGCTGACTCTAGCCGGCC
                     G  E  I  Q  P  E  L  D  A  L  D  S  L  K  I  L  D  I  S  S  N  R  I  S  G  E  I  P  L  T  L  A  G
J7005               GGCGAAATCCAGCCGGAGCTCGACGCGTTGGACAGCCTGAAGATACTTGACATAAGCAGCAACAGGATCTCTGGCGAGATCCCGCTGACTCTAGCCGGCC

AtCLV2              GGCGAAATACAACCCGAGCTTGATGCGTTGGATAGTCTAAAGATACTGGACATAAGCAACAACCATATATCCGGTGAGATCCCGCTTACTTTAGCTGGTC
                     G  E  I  Q  P  E  L  D  A  L  D  S  L  K  I  L  D  I  S  N  N  H  I  S  G  E  I  P  L  T  L  A  G
Clustal Consensus   ******** ** ** ***** ** ******** ** ** ******** ********** ****   ** ** ** *********** *** **** ** *

                          1310      1320      1330      1340      1350      1360      1370      1380      1390      1400
                    ....|....|....|....|....|....|....|....|....|....|....|....|....|....|....|....|....|....|....|....|
Ref(BnaA02g12070D)  TGAAGTCGCTAGAGATTGTGGACATAAGCTCCAACAACCTCTCGGGAAGCCTTAACGAGGCCATTACCAAATGGTCCAACCTCAAGTACCTCTCTCTCGC
                    L  K  S  L  E  I  V  D  I  S  S  N  N  L  S  G  S  L  N  E  A  I  T  K  W  S  N  L  K  Y  L  S  L  A
Hua_5               TGAAGTCGCTAGAGATTGTGGACATAAGCTCCAACAACCTCTCGGGAAGCCTTAACGAGGCCATTACCAAATGGTCCAACCTCAAGTACCTCTCTCTCGC
                    L  K  S  L  E  I  V  D  I  S  S  N  N  L  S  G  S  L  N  E  A  I  T  K  W  S  N  L  K  Y  L  S  L  A
J7005               TGAAGTCGCTAGAGATTGTGGACATAAGCTCCAACAACCTCTCGGGAAGCCTTAACGAGGCCATTACCAAATGGTCCAACCTCAAGTACCTCTCTCTCGC

AtCLV2              TGAAGTCTCTAGAGATTGTGGATATAAGTTCCAACAACCTCTCGGGGAACCTTAATGAAGCCATTACCAAATGGTCCAACCTCAAGTATCTTTCTCTTGC
                    L  K  S  L  E  I  V  D  I  S  S  N  N  L  S  G  N  L  N  E  A  I  T  K  W  S  N  L  K  Y  L  S  L  A
Clustal Consensus   ******* ************** ***** ***************** * ****** ** ***************************** ** ***** **

                          1410      1420      1430      1440      1450      1460      1470      1480      1490      1500
                    ....|....|....|....|....|....|....|....|....|....|....|....|....|....|....|....|....|....|....|....|
Ref(BnaA02g12070D)  AAGGAACAAGTTCAGCGGGACGCTTCCTTCTTGGCTGTTCAAGTTCGAAAAGATCCAAATGATGGACTACTCCAGCAACAGATTCTCATGGTTCATACCT
                      R  N  K  F  S  G  T  L  P  S  W  L  F  K  F  E  K  I  Q  M  M  D  Y  S  S  N  R  F  S  W  F  I  P
Hua_5               AAGGAACAAGTTCAGCGGGACGCTTCCTTCTTGGCTGTTCAAGTTCGACAAGATCCAAATGATGGACTACTCCAGCAACAGATTCTCATGGTTCATACCT
                      R  N  K  F  S  G  T  L  P  S  W  L  F  K  F  D  K  I  Q  M  M  D  Y  S  S  N  R  F  S  W  F  I  P
J7005               AAGGAACAAGTTCAGCGGGACGCTTCCTTCTTGGCTGTTCAAGTTCGAAAAGATCCAAATGATGGACTACTCCAGCAACAGATTCTCATGGTTCATACCT

AtCLV2              CCGGAACAAGTTTAGTGGAACACTTCCTTCTTGGTTGTTCAAGTTTGACAAAATCCAAATGATTGACTACTCCAGCAACAGATTCTCTTGGTTCATACCA
                      R  N  K  F  S  G  T  L  P  S  W  L  F  K  F  D  K  I  Q  M  I  D  Y  S  S  N  R  F  S  W  F  I  P
Clustal Consensus     ********** ** ** ** ************ ********** ** ** *********** *********************** ***********

                          1510      1520      1530      1540      1550      1560      1570      1580      1590      1600
                    ....|....|....|....|....|....|....|....|....|....|....|....|....|....|....|....|....|....|....|....|
Ref(BnaA02g12070D)  GACGATAACTTGAACAGCACACGCTTCAAGGATTTTCGGACCGCTGGA---------TCTTCAGTGCCACCGGGGAAAGTGGAGATCAAGATATCAGCAA
                     D  D  N  L  N  S  T  R  F  K  D  F  R  T  A  G           S  S  V  P  P  G  K  V  E  I  K  I  S  A
Hua_5               GACGATAACTTGAACAGCACACGCTTCAAGGATTTTCGGACCGCTGGA---------TCTTCAGTGCCACCGGGGAAAGTGGAGATCAAGATATCAGCAA
                     D  D  N  L  N  S  T  R  F  K  D  F  R  T  A  G           S  S  V  P  P  G  K  V  E  I  K  I  S  A
J7005               GACGATAACTTGAACAGCACACGCTTCAAGGATTTTCGGACCGCTGGA---------TCTTCAGTGCCACCGGGGAAAGTGGAGATCAAGATATCAGCAA

AtCLV2              GACGATAACTTAAACAGCACACGCTTCAAAGATTTTCAGACCGGTGGAGGTGAAGGATTTGCTGAGCCACCAGGAAAAGTAGAGATCAAAATATCAGCAG
                     D  D  N  L  N  S  T  R  F  K  D  F  Q  T  G  G  G  E  G  F  A  E  P  P  G  K  V  E  I  K  I  S  A
Clustal Consensus   *********** ***************** ******* ***** ****         * * * * ****** ** ***** ******** *********

                          1610      1620      1630      1640      1650      1660      1670      1680      1690      1700
                    ....|....|....|....|....|....|....|....|....|....|....|....|....|....|....|....|....|....|....|....|
Ref(BnaA02g12070D)  ATGTGGTTGCTAAAGAGGAACTAGCCTTCAGCTACGATCTCTTGTCAATGGTTGGGATCGATCTTTCCGACAATCTGTTACACGGAGAAATACCTGAAGC
                    N  V  V  A  K  E  E  L  A  F  S  Y  D  L  L  S  M  V  G  I  D  L  S  D  N  L  L  H  G  E  I  P  E  A
Hua_5               ATGTGGTTGCTAAAGAGGAACTAGCCTTCAGCTACGATCTCTTGTCAATGGTTGGGATCGATCTTTCCGACAATCTGTTACACGGAGAAATACCTGAAGC
                    N  V  V  A  K  E  E  L  A  F  S  Y  D  L  L  S  M  V  G  I  D  L  S  D  N  L  L  H  G  E  I  P  E  A
J7005               ATGTGGTTGCTAAAGAGGAACTAGCCTTCAGCTACGATCTCTTGTCAATGGTTGGGATCGATCTTTCCGACAATCTGTTACACGGAGAAATACCTGAAGC

AtCLV2              CTGTTGTTGCTAAAGATGAATTAAGTTTCAGCTACAATCTATTGTCTATGGTTGGGATCGATCTTTCTGACAATCTATTACATGGAGAGATCCCAGAAGC
                    A  V  V  A  K  D  E  L  S  F  S  Y  N  L  L  S  M  V  G  I  D  L  S  D  N  L  L  H  G  E  I  P  E  A
Clustal Consensus    *** *********** *** **   ********* **** ***** ******************** ******** ***** ***** ** ** *****

                          1710      1720      1730      1740      1750      1760      1770      1780      1790      1800
                    ....|....|....|....|....|....|....|....|....|....|....|....|....|....|....|....|....|....|....|....|
Ref(BnaA02g12070D)  TTTGTTCAGACAGAAGAACATCGAGTACTTGAACTTGTCGTACAACTTCCTTGAAGGCCAGCTTCCACGTTTAGAGAAGCTGCCGAGGCTAAAGGCCTTA
                      L  F  R  Q  K  N  I  E  Y  L  N  L  S  Y  N  F  L  E  G  Q  L  P  R  L  E  K  L  P  R  L  K  A  L
Hua_5               TTTGTTCAGACAGAAGAACATCGAGTACTTGAACTTGTCGTACAACTTCCTTGAAGGCCAGCTTCCACGTTTAGAGAAGCTGCCGAGGCTAAAGGCCTTA
                      L  F  R  Q  K  N  I  E  Y  L  N  L  S  Y  N  F  L  E  G  Q  L  P  R  L  E  K  L  P  R  L  K  A  L
J7005               TTTGTTCAGACAGAAGAACATCGAGTACTTGAACTTGTCGTACAACTTCCTCGAAGGCCAGCTTCCACGTTTAGAGAAGCTGCCGAGGCTAAAGGCCTTA

AtCLV2              TTTGTTCAGACAGAAGAATATTGAGTACTTGAACTTGTCCTACAACTTCCTTGAAGGTCAGCTTCCACGTCTAGAGAAGCTGCCAAGGTTAAAGGCCTTA
                      L  F  R  Q  K  N  I  E  Y  L  N  L  S  Y  N  F  L  E  G  Q  L  P  R  L  E  K  L  P  R  L  K  A  L
Clustal Consensus   ****************** ** ***************** *********** ***** ************ ************* *** ***********
```

```
                         1810      1820      1830      1840      1850      1860      1870      1880      1890      1900
Ref(BnaA02g12070D)  GATCTTTCACACAACTCTCTGTCAGGTCAAGTCACTGGAAATGTCTCAACTCCTCCAGGACTGACTCTTCTGAATCTGTCTCACAACTGTCTCTCTGGAA
                     D  L  S  H  N  S  L  S  G  Q  V  T  G  N  V  S  T  P  P  G  L  T  L  L  N  L  S  H  N  C  L  S  G
Hua_5               GATCTTTCACACAACTCTCTCTCAGGTCAAGTCACTGGAAATGTCTCAACTCCTCCAGGACTGACTCTTCTGAATCTGTCTCACAACTGTCTCTCTGGAA
                     D  L  S  H  N  S  L  S  G  Q  V  T  G  N  V  S  T  P  P  G  L  T  L  L  N  L  S  H  N  C  L  S  G
J7005               GATCTTTCACACAACTCTCTGTCAGGTCAAGTCACTGGAAATGTCTCAACTCCTCCAGGACTGACTCTTCTGAATCTGTCTCACAACTGTTTCTCTGGAA

AtCLV2              GATCTTTCACACAACTCTCTGTCAGGTCAAGTCATTGGAAACATCTCAGCTCCTCCAGGACTAACCCTACTGAACCTATCGCACAACTGTTTCTCTGGTA
                     D  L  S  H  N  S  L  S  G  Q  V  I  G  N  I  S  A  P  P  G  L  T  L  L  N  L  S  H  N  C  F  S  G
Clustal Consensus   ******************** ************* ******  ***** ************* ** ** ***** ** ** ********* ******* *

                         1910      1920      1930      1940      1950      1960      1970      1980      1990      2000
Ref(BnaA02g12070D)  TCGTTACTGAGAAAGAAGGGCTTGGGAAGTTTCCAGGGGCTTTGGTTGGGAACCCGGAACTTTGTGTGGAATCTTCAGGGAACAAGTGTGATCCTGCAAA
                    I  V  T  E  K  E  G  L  G  K  F  P  G  A  L  V  G  N  P  E  L  C  V  E  S  S  G  N  K  C  D  P  A  N
Hua_5               TCGTTACTGAGAAAGAAGGGCTTGGGAAGTTTCCAGGGGCTTTGGTTGGGAACCCGGAACTTTGTGTGGAATCTTCAGGGAACAAGTGTGATCCTGCAAA
                    I  V  T  E  K  E  G  L  G  K  F  P  G  A  L  V  G  N  P  E  L  C  V  E  S  S  G  N  K  C  D  P  A  N
J7005               TCGTTACTGAGAAAGAAGGGCTTGGGAAGTTTCCAGGGGCTTTGGTTGGGAACCCGGAACTTTGTGTGGAATCTTCAGGGAACAAGTGTGATCCTGCAAA

AtCLV2              TCATTACCGAGAAAGAAGGGCTCGGGAAGTTTCCAGGGGCGTTGGCCGGAAATCCGGAACTGTGTGTGGAAACTCCCGGAAGCAAGTGTGACCCGGCAAA
                    I  I  T  E  K  E  G  L  G  K  F  P  G  A  L  A  G  N  P  E  L  C  V  E  T  P  G  S  K  C  D  P  A  N
Clustal Consensus   ** **** ************** ***************** ****  ** ** ******** ********* ** * ** * ********* ** *****

                         2010      2020      2030      2040      2050      2060      2070      2080      2090      2100
Ref(BnaA02g12070D)  CATTGATGCATCACAAGAGGAGATATATCAAAACGAGTTGGTGGAAGGACCGATATCGATTTGGATATTCTGCTTAAGCGCCTTTATTAGCTTTGATTTT
                      I  D  A  S  Q  E  E  I  Y  Q  N  E  L  V  E  G  P  I  S  I  W  I  F  C  L  S  A  F  I  S  F  D  F
Hua_5               CATTGATGCATCACAAGAGGAGATATATCAAAACGAGTTGGTGGAAGGACCGATATCGATTTGGATATTCTGCTTAAGCGCCTTTATTAGCTTTGATTTT
                      I  D  A  S  Q  E  E  I  Y  Q  N  E  L  V  E  G  P  I  S  I  W  I  F  C  L  S  A  F  I  S  F  D  F
J7005               CATTGATGCATCACAAGAGGAGATATATCAAAACGAGTTGGTGGAAGGACCGATATCGATTTGGATATTCTGCTTAAGCGCCTTTATTAGCTTTGATTTT

AtCLV2              CATTGATGCATCACAAGAGGAAATATATCAAAACGAATTGGTGGAAGGACCGATATCTATTTGGATATTCTGCTTAAGTGCGTTTATTAGCTTCGATTTT
                      I  D  A  S  Q  E  E  I  Y  Q  N  E  L  V  E  G  P  I  S  I  W  I  F  C  L  S  A  F  I  S  F  D  F
Clustal Consensus   ********************* *************** ******************** ******************** ** *********** ******

                         2110      2120      2130      2140      2150      2160
Ref(BnaA02g12070D)  GGAGTGTTGGGTATCTTCTGCTCATCTCGTGCTCGGAGTTATATTCTCCAGACCAAAGGTTAA
                     G  V  L  G  I  F  C  S  S  R  A  R  S  Y  I  L  Q  T  K  G  *
Hua_5               GGAGTGTTGGGTATCTTCTGCTCATCTCGTGCTCGGAGTTATATTCTCCAGACCAAAGGTTAA
                     G  V  L  G  I  F  C  S  S  R  A  R  S  Y  I  L  Q  T  K  G  *
J7005               GGAGTGTTGGGTATCTTCTGCTCATCTCGTGCTCGGAGTTATATTCTCCAGACCAAAGGTTAA

AtCLV2              GGAGTGTTAGGTATCTTTTGCTCAGCTCGTGCTCGTAGTTATATTCTTCAGACCAAAGCTTAA
                     G  V  L  G  I  F  C  S  A  R  A  R  S  Y  I  L  Q  T  K  A  *
Clustal Consensus   ******** ******** ****** ********** *********** ********** ****
```

Supplementary Figure S5. Alignment of the nucleotide sequences of *CLV2* genes (BnaA02g12070D; no intron) and predicated CLV2 amino acid sequences from *Brassica napus* reference genome (Darmor-*bzh*), the two parents (Hua_5 and J7005), and Arabidopsis (AT1G65380.1). The red box refers to the premature termination of amino acid translation caused by point mutation in J7005.

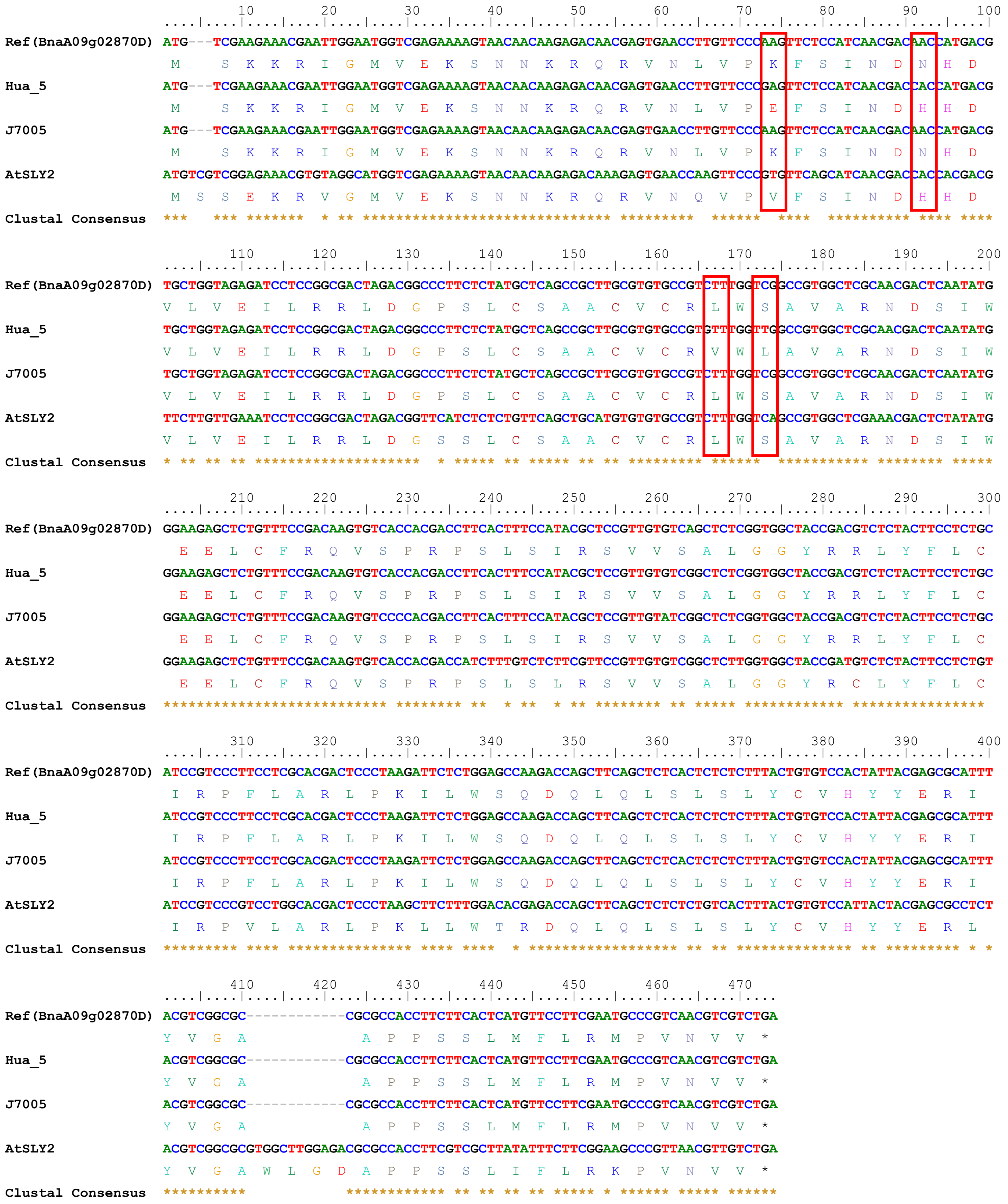

Supplementary Figure S6. Alignment of the nucleotide sequences of *SLY2* genes (BnaA09g02870D; no intron) and predicated SLY2 amino acid sequences from *Brassica napus* reference genome (Darmor-*bzh*), the two parents (Hua_5 and J7005), and Arabidopsis (AT5G48170.1). The red boxes refer to the different amino acids between Hua_5 and J7005 caused by point mutation.

```
                            10        20        30        40        50        60        70        80        90       100
                   ....|....|....|....|....|....|....|....|....|....|....|....|....|....|....|....|....|....|....|....|
Ref_Genome         ATGTTCAATCCTTTCACAACTCATCACAGGTAACCGAAAATCAATTATTTTTCTGTTAGTTAAAAAAAATTGCAGCAACTCTCTGTCTTTCTCTTCCATG
Ref_CDS            ATGTTCAATCCTTTCACAACTCATCACAG-----------------------------------------------------------------------
                    M  F  N  P  F  T  T  H  H  S
Hua_5_Genome       ATGTTCAATCCTTTCACAACTCATCACAGGTAACCGAAAATCAATTATTTTTCTGTTAGTTAAAAAAAATTGCAGCAACTCTCTGTCTTTCTCTTCCATG
Hua_5_CDS          ATGTTCAATCCTTTCACAACTCATCACAG-----------------------------------------------------------------------
                    M  F  N  P  F  T  T  H  H  S
J7005_Genome       ATGTTCAATCCTTTCACAACTCATCACAGGTAACCGAAAATCAATTATTTTTCTGTTAGTTAAAAAAAATTGCAGCAACTCTCTGTCTTTCTCTTCCATG
J7005_CDS          ATGTTCAATCCTTTCACAACTCATCACAG-----------------------------------------------------------------------
                    M  F  N  P  F  T  T  H  H  S
AtAHK4             ATGTTCAATCCTCTCACAACTCATTACAG-----------------------------------------------------------------------
                    M  F  N  P  L  T  T  H  Y  S
Clustal Consensus  ************ *********** ****

                           110       120       130       140       150       160       170       180       190       200
                   ....|....|....|....|....|....|....|....|....|....|....|....|....|....|....|....|....|....|....|....|
Ref_Genome         TGCGCCTCTCGAAAATTTCGGCTTTATTTTTATTTTTATTATGCTTCGTTGTTTTTGTGCTGTTGTGTTTTCTTTGTTTGGTCAAAGCTTTGTTTTTTTG
Ref_CDS            ----------------------------------------------------------------------------------------------------

Hua_5_Genome       TGCGCCTCTCGAAAATTTCGGCTTTATTTTTATTTTTATTATGCTTCGTTGTTTTTGTGCTGTTGTGTTTTCTTTGTTTGGTCAAAGCTTTGTTTTTTTG
Hua_5_CDS          ----------------------------------------------------------------------------------------------------

J7005_Genome       TGCGCCTCTCGAAAATTTCGGCTTTATTTTTATTTTTATTATGCTTCGTTGTTTTTGTGCTGTTGTGTGTTCTTTGTTTGGTCAAAGCTTTGTTTTTTTG
J7005_CDS          ----------------------------------------------------------------------------------------------------

AtAHK4             ----------------------------------------------------------------------------------------------------

Clustal Consensus

                           210       220       230       240       250       260       270       280       290       300
                   ....|....|....|....|....|....|....|....|....|....|....|....|....|....|....|....|....|....|....|....|
Ref_Genome         TTTGTTTGGATTTAAGACAGTGAACATAGCTGTGAACTTGTTCCGAGAAGAAACATCAACTAAACATTTCACGCCTGTAAAGAACCTTATGATTAGTGTC
Ref_CDS            ----------------------------------------------------------------------------------------------------

Hua_5_Genome       TTTGTTTGGATTTAAGACAGTGAACATAGCTGTGAACTTGTTCCGAGAAGAAACATCAACTAAACATTTCACGCCTGTAAAGAACCTTATGATTAGTGTC
Hua_5_CDS          ----------------------------------------------------------------------------------------------------

J7005_Genome       TTTGTTTGGATTTAAGACAGTGAACATAGCTGTGAACTTGTTCCGAGAAGAAACATCAACTAAACATTTCACGCCTGTAAAGAACCTTATGATTAGTGTC
J7005_CDS          ----------------------------------------------------------------------------------------------------

AtAHK4             ----------------------------------------------------------------------------------------------------

Clustal Consensus

                           310       320       330       340       350       360       370       380       390       400
                   ....|....|....|....|....|....|....|....|....|....|....|....|....|....|....|....|....|....|....|....|
Ref_Genome         TTGCTTAATCATTCCTCTGACAATTAATTAACCGAAACCCCCATTTCTTTAATTTATTTTAAACTAATGCTCACCTCTGTAGCAAACCTTCGGAAACTGA
Ref_CDS            ----------------------------------------------------------------------------------------------------

Hua_5_Genome       TTGCTTAATCATTCCTCTGACAATTAATTAACCGAAACCCCCATTTCTTTAATTTATTTTAAACTAATGCTCACCTCTGTAGCAAACCTTCGGAAACTGA
Hua_5_CDS          ----------------------------------------------------------------------------------------------------

J7005_Genome       TTGCTTAATCATTCCTCTGACAATTAATTAACCGAAACCCCCATTTCTTTAATTTATTTTAAACTAATGCTCACCTCTGTAGCAAACCTTCGGAAACTGA
J7005_CDS          ----------------------------------------------------------------------------------------------------

AtAHK4             ----------------------------------------------------------------------------------------------------

Clustal Consensus

                           410       420       430       440       450       460       470       480       490       500
                   ....|....|....|....|....|....|....|....|....|....|....|....|....|....|....|....|....|....|....|....|
Ref_Genome         GCAAAGTTCCTTCTTTTGAGAGTAGTCCCATGCGTTGAGAAAAAGTTTCTTGAATTTGCCAAAAAAAAAACCTCTCTTTTTGGTTTATGCTTTGTTAATG
Ref_CDS            ----------------------------------------------------------------------------------------------------

Hua_5_Genome       GCAAAGTTCCTTCTTTTGAGAGTAGTCCCATGCGTTGAGAAAAAGTTTCTTGAATTTGCCAAAAAAAAAACCTCTCTTTTTGGTTTATGCTTTGTTAATG
Hua_5_CDS          ----------------------------------------------------------------------------------------------------

J7005_Genome       GCAAAGTTCCTTCTTTTGAGAGTAGTCCCATGCGTTGAGAAAAAGTTTCTTGAATTTGCCAAAAAAAAAACCTCTCTTTTTGGTTTATGCTTTGTTAATG
J7005_CDS          ----------------------------------------------------------------------------------------------------
```

```
AtAHK4             ----------------------------------------------------------------------------------------------------

Clustal Consensus

                            510       520       530       540       550       560       570       580       590       600
                   ....|....|....|....|....|....|....|....|....|....|....|....|....|....|....|....|....|....|....|....|
Ref_Genome         TATTGATAAACACGCAAAACAATGTTTATGGTAAGCTCTAATCATACCACTAGGCATGCTTGTTATGTTGTTATATGAATGCGTAAGTCACACACACACT
Ref_CDS            ----------------------------------------------------------------------------------------------------

Hua_5_Genome       TATTGATAAACACGCAAAACAATGTTTATGGTAAGCTCTAATCATACCACTAGGCATGCTTGTTATGTTGTTATATGAATGCGTAAGTCACACACACACT
Hua_5_CDS          ----------------------------------------------------------------------------------------------------

J7005_Genome       TATTGATAAACACGCAAAACAATGTTTATGGTAAGCTCTAATCATACCACTAGGCATGCTTGTTATGTTGTTATATGAATGCGTAAGTCACACACACACT
J7005_CDS          ----------------------------------------------------------------------------------------------------

AtAHK4             ----------------------------------------------------------------------------------------------------

Clustal Consensus

                            610       620       630       640       650       660       670       680       690       700
                   ....|....|....|....|....|....|....|....|....|....|....|....|....|....|....|....|....|....|....|....|
Ref_Genome         AACATTCCTCTGTCTCTCCATTGTTTTGTAGCTCAGATATGAACTGGGCACTCAACAACCCAAATCCTGAAGAAAAGGAGCCAAGAACAACA--------
Ref_CDS            -------------------------------CTCAGATATGAACTGGGCACTCAACAACCCAAATCCTGAAGAAAAGGAGCCAAGAACAACA--------
                                                   S  D  M  N  W  A  L  N  N  P  N  P  E  E  K  E  P  R  T  T
Hua_5_Genome       AACATTCCTCTGTCTCTCCATTGTTTTGTAGCTCAGATATGAACTGGGCACTCAACAACCCAAATCCTGAAGAAAAGGAGCCAAGAACAACA--------
Hua_5_CDS          -------------------------------CTCAGATATGAACTGGGCACTCAACAACCCAAATCCTGAAGAAAAGGAGCCAAGAACAACA--------
                                                   S  D  M  N  W  A  L  N  N  P  N  P  E  E  K  E  P  R  T  T
J7005_Genome       AACATTCCTCTGTCTCTCCATTGTTTTGTAGCTCAGATATGAACTGGGCACTCAACAACCCAAATCCTGAAGGAAAGGAGCCAAGAACAACA--------
J7005_CDS          -------------------------------CTCAGATATGAACTGGGCACTCAACAACCCAAATCCTGAAGGAAAGGAGCCAAGAACAACA--------
                                                   S  D  M  N  W  A  L  N  N  P  N  P  E  G  K  E  P  R  T  T
AtAHK4             -------------------------------CTCAGATATGAACTGGGCACTCAACAATCATCAAGAAGAAGAAGAAGAGCCACGAAGAATTGAAATTTC
                                                   S  D  M  N  W  A  L  N  N  H  Q  E  E  E  E  E  P  R  R  I  E  I  S
Clustal Consensus                                  *************************** *   *    **** * * ****** *** **

                            710       720       730       740       750       760       770       780       790       800
                   ....|....|....|....|....|....|....|....|....|....|....|....|....|....|....|....|....|....|....|....|
Ref_Genome         ----------------------ACCCAAAGCAGCGATTTTTATCATCTGGGCG---------CTAAAGATTCGCCACAAAAGTCCAGAAAAATCGATTTC
Ref_CDS            ----------------------ACCCAAAGCAGCGATTTTTATCATCTGGGCG---------CTAAAGATTCGCCACAAAAGTCCAGAAAAATCGATTTC
                                          T  Q  S  S  D  F  Y  H  L  G            A  K  D  S  P  Q  K  S  R  K  I  D  F
Hua_5_Genome       ----------------------ACCCAAAGCAGCGATTTTTATCATCTGGGCG---------CTAAAGATTCGCCACAAAAGTCCAGAAAAATCGATTTC
Hua_5_CDS          ----------------------ACCCAAAGCAGCGATTTTTATCATCTGGGCG---------CTAAAGATTCGCCACAAAAGTCCAGAAAAATCGATTTC
                                          T  Q  S  S  D  F  Y  H  L  G            A  K  D  S  P  Q  K  S  R  K  I  D  F
J7005_Genome       ----------------------ACCCAAAGCAGCGATTTTTATCATCTGGGCG---------CTAAAGATTCGCCACAAAAGTTCAGAAAAATCGATTTC
J7005_CDS          ----------------------ACCCAAAGCAGCGATTTTTATCATCTGGGCG---------CTAAAGATTCGCCACAAAAGTTCAGAAAAATCGATTTC
                                          T  Q  S  S  D  F  Y  H  L  G            A  K  D  S  P  Q  K  F  R  K  I  D  F
AtAHK4             TGATTCCGAGTCACTAGAAAACTTGAAAAGCAGCGATTTTTATCAACTGGGTGGTGGTGGTGCTCTGAATTCGTCAGAAAAGCCGAGAAAGATCGATTTT
                    D  S  E  S  L  E  N  L  K  S  S  D  F  Y  Q  L  G  G  G  G  A  L  N  S  S  E  K  P  R  K  I  D  F
Clustal Consensus                            ******************* ***** *         **    ***** ** *****   ***** ********

                            810       820       830       840       850       860       870       880       890       900
                   ....|....|....|....|....|....|....|....|....|....|....|....|....|....|....|....|....|....|....|....|
Ref_Genome         TGGCGTTCTGGGTTGATGGGTTTCGCCAAGATGCA------------------GCATTCAGTAGCGGTGAAGATGAACAACGGTAATAATAACGACCAAG
Ref_CDS            TGGCGTTCTGGGTTGATGGGTTTCGCCAAGATGCA------------------GCATTCAGTAGCGGTGAAGATGAACAACGGTAATAATAACGACCAAG
                    W  R  S  G  L  M  G  F  A  K  M  Q                  H  S  V  A  V  K  M  N  N  G  N  N  N  D  Q
Hua_5_Genome       TGGCGTTCTGGGTTGATGGGTTTCGCCAAGATGCA------------------GCATTCAGTAGCGGTGAAGATGAACAACGGTAATAATAACGACCAAG
Hua_5_CDS          TGGCGTTCTGGGTTGATGGGTTTCGCCAAGATGCA------------------GCATTCAGTAGCGGTGAAGATGAACAACGGTAATAATAACGACCAAG
                    W  R  S  G  L  M  G  F  A  K  M  Q                  H  S  V  A  V  K  M  N  N  G  N  N  N  D  Q
J7005_Genome       TGGCGTTCTGGGTTGATGGGTTTCGCCAAGATGCA------------------GCATTCAGTGGCGGTGAAGATGAACAACGGTAATAATAACGACCAAG
J7005_CDS          TGGCGTTCTGGGTTGATGGGTTTCGCCAAGATGCA------------------GCATTCAGTGGCGGTGAAGATGAACAACGGTAATAATAACGACCAAG
                    W  R  S  G  L  M  G  F  A  K  M  Q                  H  S  V  A  V  K  M  N  N  G  N  N  N  D  Q
AtAHK4             TGGCGTTCGGGGTTGATGGGTTTTGCGAAGATGCAGCAGCAGCAACAGCTTCAGCATTCAGTGGCGGTGAAGATGAACAATAATAATAATAACGATCTAA
                    W  R  S  G  L  M  G  F  A  K  M  Q  Q  Q  Q  Q  L  Q  H  S  V  A  V  K  M  N  N  N  N  N  N  D  L
Clustal Consensus  ******** ************** ** ********                  ********* ***************** ************ * *

                            910       920       930       940       950       960       970       980       990      1000
                   ....|....|....|....|....|....|....|....|....|....|....|....|....|....|....|....|....|....|....|....|
Ref_Genome         TGGGTAACAAAAAGGGGTCGACTTTCATACAGGAACACAGAGCTTTATTACCAAAGGGCTTGATTCTGTGGACCATCATCGTTGGGTTTATAAGCAGAGG
Ref_CDS            TGGGTAACAAAAAGGGGTCGACTTTCATACAGGAACACAGAGCTTTATTACCAAAGGGCTTGATTCTGTGGACCATCATCGTTGGGTTTATAAGCAGAGG
                   V  G  N  K  K  G  S  T  F  I  Q  E  H  R  A  L  L  P  K  G  L  I  L  W  T  I  I  V  G  F  I  S  R  G
Hua_5_Genome       TGGGTAACAAAAAGGGGTCGACTTTCATACAGGAACACAGAGCTTTATTACCAAAGGGCTTGATTCTGTGGACCATCATCGTTGGGTTTATAAGCAGAGG
Hua_5_CDS          TGGGTAACAAAAAGGGGTCGACTTTCATACAGGAACACAGAGCTTTATTACCAAAGGGCTTGATTCTGTGGACCATCATCGTTGGGTTTATAAGCAGAGG
```

```
                    V  G  N  K  K  G  S  T  F  I  Q  E  H  R  A  L  L  P  K  G  L  I  L  W  T  I  I  V  G  F  I  S  R  G
J7005_Genome        TGGGTAACAAAAAGGGGTCGACTTTCATACAGGAACACAGAGCTTTATTACCAAAGGGCTTGATTCTGTGGACCATCATCGTTGGGTTTATAAGCAGAGG
J7005_CDS           TGGGTAACAAAAAGGGGTCGACTTTCATACAGGAACACAGAGCTTTATTACCAAAGGGCTTGATTCTGTGGACCATCATCGTTGGGTTTATAAGCAGAGG
                    V  G  N  K  K  G  S  T  F  I  Q  E  H  R  A  L  L  P  K  G  L  I  L  W  T  I  I  V  G  F  I  S  R  G
AtAHK4              TGGGTAATAAAAAAGGGTCAACTTTCATACAAGAACATCGAGCATTGTTACCAAAAGCTTTGATTCTGTGGATCATCATTGTTGGGTTTATAAGCAGTGG
                    M  G  N  K  K  G  S  T  F  I  Q  E  H  R  A  L  L  P  K  A  L  I  L  W  I  I  I  V  G  F  I  S  S  G
Clustal Consensus   ******* ***** ***** *********** *****  **** ** ******** *  ************* ****** ***************** **

                             1010      1020      1030      1040      1050      1060      1070      1080      1090      1100
                    ....|....|....|....|....|....|....|....|....|....|....|....|....|....|....|....|....|....|....|....|
Ref_Genome          GATTTACCGGTGGATGGATGATACCAGCAAGGTCAGAAGAGAAGAGGTTTTGGTTAGTATGTGTGATCAGAGAGCGAGGATGTTGCAGGATCAGTTTAGT
Ref_CDS             GATTTACCGGTGGATGGATGATACCAGCAAGGTCAGAAGAGAAGAGGTTTTGGTTAGTATGTGTGATCAGAGAGCGAGGATGTTGCAGGATCAGTTTAGT
                      I  Y  R  W  M  D  D  T  S  K  V  R  R  E  E  V  L  V  S  M  C  D  Q  R  A  R  M  L  Q  D  Q  F  S
Hua_5_Genome        GATTTACCGGTGGATGGATGATACCAGCAAGGTCAGAAGAGAAGAGGTTTTGGTTAGTATGTGTGATCAGAGAGCGAGGATGTTGCAGGATCAGTTTAGT
Hua_5_CDS           GATTTACCGGTGGATGGATGATACCAGCAAGGTCAGAAGAGAAGAGGTTTTGGTTAGTATGTGTGATCAGAGAGCGAGGATGTTGCAGGATCAGTTTAGT
                      I  Y  R  W  M  D  D  T  S  K  V  R  R  E  E  V  L  V  S  M  C  D  Q  R  A  R  M  L  Q  D  Q  F  S
J7005_Genome        GATTTACCGGTGGATGGATGATACCAGCAAGGTCAGAAGAGAAGAGGTTTTGGTTAGTATGTGTGATCAGAGAGCGAGGATGTTGCAGGATCAGTTTAGT
J7005_CDS           GATTTACCGGTGGATGGATGATACCAGCAAGGTCAGAAGAGAAGAGGTTTTGGTTAGTATGTGTGATCAGAGAGCGAGGATGTTGCAGGATCAGTTTAGT
                      I  Y  R  W  M  D  D  T  S  K  V  R  R  E  E  V  L  V  S  M  C  D  Q  R  A  R  M  L  Q  D  Q  F  S
AtAHK4              GATTTATCAGTGGATGGATGATGCTAATAAGATTAGAAGGGAAGAGGTTTTGGTCAGCATGTGTGATCAAAGAGCTAGAATGTTGCAGGATCAATTTAGT
                      I  Y  Q  W  M  D  D  A  N  K  I  R  R  E  E  V  L  V  S  M  C  D  Q  R  A  R  M  L  Q  D  Q  F  S
Clustal Consensus   ****** * ************* * *  *** * ***** ************** ** *********** ***** ** ************** ******

                             1110      1120      1130      1140      1150      1160      1170      1180      1190      1200
                    ....|....|....|....|....|....|....|....|....|....|....|....|....|....|....|....|....|....|....|....|
Ref_Genome          GTTAGTGTTAACCATGTCCATGCTTTGGCTATTCTTGTCTCAACGTTTCATTACCACAAGAATCCATCTGCAATTGATCAGGTCAGTGTCTGCCTTATTA
Ref_CDS             GTTAGTGTTAACCATGTCCATGCTTTGGCTATTCTTGTCTCAACGTTTCATTACCACAAGAATCCATCTGCAATTGATCA--------------------
                     V  S  V  N  H  V  H  A  L  A  I  L  V  S  T  F  H  Y  H  K  N  P  S  A  I  D  Q
Hua_5_Genome        GTTAGTGTTAACCATGTCCATGCTTTGGCTATTCTCGTCTCAACGTTTCATTACCACAAGAATCCATCTGCAATTGATCAGGTCAGTGTCTGCCTTATTA
Hua_5_CDS           GTTAGTGTTAACCATGTCCATGCTTTGGCTATTCTCGTCTCAACGTTTCATTACCACAAGAATCCATCTGCAATTGATCA--------------------
                     V  S  V  N  H  V  H  A  L  A  I  L  V  S  T  F  H  Y  H  K  N  P  S  A  I  D  Q
J7005_Genome        GTTAGTGTTAACCATGTCCATGCTTTGGCTATTCTTGTCTCAACGTTTCATTACCACAAGAATCCATCTGCAATTGATCAGGTCAGTGTCTGCCTTATTA
J7005_CDS           GTTAGTGTTAACCATGTCCATGCTTTGGCTATTCTTGTCTCAACGTTTCATTACCACAAGAATCCATCTGCAATTGATCA--------------------
                     V  S  V  N  H  V  H  A  L  A  I  L  V  S  T  F  H  Y  H  K  N  P  S  A  I  D  Q
AtAHK4              GTTAGTGTTAATCATGTTCATGCTTTGGCTATTCTCGTCTCCACTTTTCATTACCACAAGAACCCTTCTGCAATTGATCA--------------------
                     V  S  V  N  H  V  H  A  L  A  I  L  V  S  T  F  H  Y  H  K  N  P  S  A  I  D  Q
Clustal Consensus   *********** ***** ***************** ***** ** ***************** ** **************

                             1210      1220      1230      1240      1250      1260      1270      1280      1290      1300
                    ....|....|....|....|....|....|....|....|....|....|....|....|....|....|....|....|....|....|....|....|
Ref_Genome          AAGGTTAAAGCTTTAGAGAAAAAGATCAAGATTTGTTCATATTTGATTTATAATTGAATAGTTGACTTTTCTTGAAATTCTTTTAATTACTAGTCTAGTT
Ref_CDS             ----------------------------------------------------------------------------------------------------

Hua_5_Genome        AAGGTTAAAGCTTTAGAGAAAAAGATCAAGATTTGTTCATATTTGATTTATAATTGAATAGTTGACTTTTCTTGAATTTTTTTTTATTACTAGTCTAGTT
Hua_5_CDS           ----------------------------------------------------------------------------------------------------

J7005_Genome        AAGGTTAAAGCTTTAGAGAAAAAGATCAAGATTTGTTCATATTTGATTTATAATTGAATAGTTGACTTTTCTTGAAATTCTTTTAATTACTAGTCTAGTT
J7005_CDS           ----------------------------------------------------------------------------------------------------

AtAHK4              ----------------------------------------------------------------------------------------------------

Clustal Consensus

                             1310      1320      1330      1340      1350      1360      1370      1380      1390      1400
                    ....|....|....|....|....|....|....|....|....|....|....|....|....|....|....|....|....|....|....|....|
Ref_Genome          CTGTTTCTGAATTAAAGTTTTACATTCATTGTAAAAATTGTTTTGCATCGAATAAATTTAACATTTCATTCCAAAAAATATAAAAAATTTGCAGGGGACA
Ref_CDS             ---------------------------------------------------------------------------------------------GGGGACA
                                                                                                               G  T
Hua_5_Genome        CTGTTTCTGAATTAAAGTTTCACATTCATTGTAAAAA-TGTTTTGCATCGAATAAATTTAACATTTCATTCCAAAAAATATAAAAAATTTGCAGGGGACA
Hua_5_CDS           ---------------------------------------------------------------------------------------------GGGGACA
                                                                                                               G  T
J7005_Genome        CTGTTTCTGAATTAAAGTTTTACATTCATTGTAAAAATTGTTTTGCATCGAATAAATTTAACATTTCATTCCAAAAAATATAAAAAATTTGCAGGGGACA
J7005_CDS           ---------------------------------------------------------------------------------------------GGGGACA
                                                                                                               G  T
AtAHK4              ---------------------------------------------------------------------------------------------GGAGACA
                                                                                                               E  T
Clustal Consensus                                                                                                ** ****

                             1410      1420      1430      1440      1450      1460      1470      1480      1490      1500
                    ....|....|....|....|....|....|....|....|....|....|....|....|....|....|....|....|....|....|....|....|
Ref_Genome          TTTGCGGACTACACAGCAAGAACAGCGTTTGAGAGACCGTTGCTAAGTGGAGTGGCATACGCAGAGAAAGTTGTGAATGCTGAGAGGGAGATGTTTGAGA
```

```
Ref_CDS             TTTGCGGACTACACAGCAAGAACAGCGTTTGAGAGACCGTTGCTAAGTGGAGTGGCATACGCAGAGAAAGTTGTGAATGCTGAGAGGGAGATGTTTGAGA
                     F  A  D  Y  T  A  R  T  A  F  E  R  P  L  L  S  G  V  A  Y  A  E  K  V  V  N  A  E  R  E  M  F  E
Hua_5_Genome        TTTGCGGACTACACAGCAAGAACAGCGTTTGAGAGACCGTTGCTGAGCGGAGTGGCTTACGCAGAGAAAGTTGTGAATGCTGAGAGGGAGATGTTTGAGA
Hua_5_CDS           TTTGCGGACTACACAGCAAGAACAGCGTTTGAGAGACCGTTGCTGAGCGGAGTGGCTTACGCAGAGAAAGTTGTGAATGCTGAGAGGGAGATGTTTGAGA
                     F  A  D  Y  T  A  R  T  A  F  E  R  P  L  L  S  G  V  A  Y  A  E  K  V  V  N  A  E  R  E  M  F  E
J7005_Genome        TTTGCGGACTACACAGCAAGAACAGCGTTTGAGAGACCGTTGCTAAGTGGAGTGGCATACGCAGAGAAAGTTGTGAATGCTGAGAGGGAGATGTTTGAGA
J7005_CDS           TTTGCGGACTACACAGCAAGAACAGCGTTTGAGAGACCGTTGCTAAGTGGAGTGGCATACGCAGAGAAAGTTGTGAATGCTGAGAGGGAGATGTTTGAGA
                     F  A  D  Y  T  A  R  T  A  F  E  R  P  L  L  S  G  V  A  Y  A  E  K  V  V  N  A  E  R  E  M  F  E
AtAHK4              TTTGCGGAGTACACGGCAAGAACAGCATTTGAGAGACCGTTGCTAAGTGGAGTGGCTTATGCTGAAAAAGTTGTGAATTTTGAGAGGGAGATGTTTGAGC
                     F  A  E  Y  T  A  R  T  A  F  E  R  P  L  L  S  G  V  A  Y  A  E  K  V  V  N  F  E  R  E  M  F  E
Clustal Consensus   ******** ***** *********** ***************** ** ******** ** ** ** ************  ******************* 

                             1510      1520      1530      1540      1550      1560      1570      1580      1590      1600
                    ....|....|....|....|....|....|....|....|....|....|....|....|....|....|....|....|....|....|....|....|
Ref_Genome          GTCAGCACAATTGGGTTATAAAGACAATGGACACAGGAGAGCCTTCACCTGTTAGGGACGAGTATGCTCCCGTCATCTTCTCTCAAGACAGTGTCTCATA
Ref_CDS             GTCAGCACAATTGGGTTATAAAGACAATGGACACAGGAGAGCCTTCACCTGTTAGGGACGAGTATGCTCCCGTCATCTTCTCTCAAGACAGTGTCTCATA
                    S  Q  H  N  W  V  I  K  T  M  D  T  G  E  P  S  P  V  R  D  E  Y  A  P  V  I  F  S  Q  D  S  V  S  Y
Hua_5_Genome        GTCAGCACAATTGGGTTATAAAGACAATGGACACAGGAGAGCCTTCACCTGTTAGGGACGAGTATGCTCCCGTCATCTTCTCTCAAGACAGTGTCTCATA
Hua_5_CDS           GTCAGCACAATTGGGTTATAAAGACAATGGACACAGGAGAGCCTTCACCTGTTAGGGACGAGTATGCTCCCGTCATCTTCTCTCAAGACAGTGTCTCATA
                    S  Q  H  N  W  V  I  K  T  M  D  T  G  E  P  S  P  V  R  D  E  Y  A  P  V  I  F  S  Q  D  S  V  S  Y
J7005_Genome        GTCAGCACAATTGGGTTATAAAGACAATGGACACAGGAGAGCCTTCACCTGTTAGGGACGAGTATGCTCCCGTCATCTTCTCTCAAGACAGTGTCTCATA
J7005_CDS           GTCAGCACAATTGGGTTATAAAGACAATGGACACAGGAGAGCCTTCACCTGTTAGGGACGAGTATGCTCCCGTCATCTTCTCTCAAGACAGTGTCTCATA
                    S  Q  H  N  W  V  I  K  T  M  D  T  G  E  P  S  P  V  R  D  E  Y  A  P  V  I  F  S  Q  D  S  V  S  Y
AtAHK4              GGCAGCACAATTGGGTTATAAAGACAATGGATAGAGGAGAGCCTTCACCGGTTAGGGATGAGTATGCTCCTGTTATATTCTCTCAAGATAGTGTCTCTTA
                    R  Q  H  N  W  V  I  K  T  M  D  R  G  E  P  S  P  V  R  D  E  Y  A  P  V  I  F  S  Q  D  S  V  S  Y
Clustal Consensus   * ***************************** * *************** ******** *********** ** ** *********** ******** **

                             1610      1620      1630      1640      1650      1660      1670      1680      1690      1700
                    ....|....|....|....|....|....|....|....|....|....|....|....|....|....|....|....|....|....|....|....|
Ref_Genome          CCTCGAGTTACTAGACATGATGTCAGGAGAGGTAATTATTTACAAGTACTTCTTGTTTTTGGATATATCTTAGTTCTGAAAACTTTTGCTTACAGGAGGA
Ref_CDS             CCTCGAGTTACTAGACATGATGTCAGGAGA----------------------------------------------------------------GGAGGA
                     L  E  L  L  D  M  M  S  G  E                                                                E  D
Hua_5_Genome        CCTCGAGTCACTAGACATGATGTCAGGAGAGGTAATTATTGACAGATACTTCTTGTTTTTGGATATATCTTAGTTCTGAAAACTTTTGCTTACAGGAGGA
Hua_5_CDS           CCTCGAGTCACTAGACATGATGTCAGGAGA----------------------------------------------------------------GGAGGA
                     L  E  S  L  D  M  M  S  G  E                                                                E  D
J7005_Genome        CCTCGAGTTACTAGACATGATGTCAGGAGAGGTAATTATTTACAAGTACTTCTTGTTTTTGGATATATCTTAGTTCTGAAAACTTTTGCTTACAGGAGGA
J7005_CDS           CCTCGAGTTACTAGACATGATGTCAGGAGA----------------------------------------------------------------GGAGGA
                     L  E  L  L  D  M  M  S  G  E                                                                E  D
AtAHK4              CCTTGAGTCACTCGATATGATGTCAGGCGA----------------------------------------------------------------GGAGGA
                     L  E  S  L  D  M  M  S  G  E                                                                E  D
Clustal Consensus   *** **** *** ** *********** **                                                                ******

                             1710      1720      1730      1740      1750      1760      1770      1780      1790      1800
                    ....|....|....|....|....|....|....|....|....|....|....|....|....|....|....|....|....|....|....|....|
Ref_Genome          TAGAGAGAACATTTTGAGAGCTAGAGAGACAGGGAAAGCTGTCTTGACAAGCCCTTTTAGACTACTGGCTTCTCACCATCTAGGCGTTGTGTTGACCTTC
Ref_CDS             TAGAGAGAACATTTTGAGAGCTAGAGAGACAGGGAAAGCTGTCTTGACAAGCCCTTTTAGACTACTGGCTTCTCACCATCTAGGCGTTGTGTTGACCTTC
                      R  E  N  I  L  R  A  R  E  T  G  K  A  V  L  T  S  P  F  R  L  L  A  S  H  H  L  G  V  V  L  T  F
Hua_5_Genome        TAGAGAGAACATTTTGAGAGCTAGAGAGACAGGGAAAGCTGTCTTGACAAGCCCTTTTAGGCTACTGGCTTCTCACCATCTAGGAGTCGTGTTGACCTTC
Hua_5_CDS           TAGAGAGAACATTTTGAGAGCTAGAGAGACAGGGAAAGCTGTCTTGACAAGCCCTTTTAGGCTACTGGCTTCTCACCATCTAGGAGTCGTGTTGACCTTC
                      R  E  N  I  L  R  A  R  E  T  G  K  A  V  L  T  S  P  F  R  L  L  A  S  H  H  L  G  V  V  L  T  F
J7005_Genome        TAGAGAGAACATTTTGAGAGCTAGAGAGACAGGGAAAGCTGTCTTGACAAGCCCTTTTAGACTACTGGCTTCTCACCATCTAGGCGTTGTGTTGACCTTC
J7005_CDS           TAGAGAGAACATTTTGAGAGCTAGAGAGACAGGGAAAGCTGTCTTGACAAGCCCTTTTAGACTACTGGCTTCTCACCATCTAGGCGTTGTGTTGACCTTC
                      R  E  N  I  L  R  A  R  E  T  G  K  A  V  L  T  S  P  F  R  L  L  A  S  H  H  L  G  V  V  L  T  F
AtAHK4              TCGTGAGAATATTTTGCGAGCTAGAGAAACCGGAAAAGCTGTCTTGACTAGCCCTTTTAGGTTGTTGGAAACTCACCATCTCGGAGTTGTGTTGACATTC
                      R  E  N  I  L  R  A  R  E  T  G  K  A  V  L  T  S  P  F  R  L  L  E  T  H  H  L  G  V  V  L  T  F
Clustal Consensus   * * ***** ****** ********** ** ** ************** ***********  *  ***   ********** ** ** ******** ***

                             1810      1820      1830      1840      1850      1860      1870      1880      1890      1900
                    ....|....|....|....|....|....|....|....|....|....|....|....|....|....|....|....|....|....|....|....|
Ref_Genome          CCTGTGTACAAAGCCTCTCTTCCTAAAAACCCCACCGTCCAAGAGCGTATAGCAGCCACCGCAGGGTACCTCGGCGGCGCGTTTGACGTTGAGTCTCTCG
Ref_CDS             CCTGTGTACAAAGCCTCTCTTCCTAAAAACCCCACCGTCCAAGAGCGTATAGCAGCCACCGCAGGGTACCTCGGCGGCGCGTTTGACGTTGAGTCTCTCG
                      P  V  Y  K  A  S  L  P  K  N  P  T  V  Q  E  R  I  A  A  T  A  G  Y  L  G  G  A  F  D  V  E  S  L
Hua_5_Genome        CCTGTGTACAAAGCCTCTCTTCCTAAAAACCCCACCGTCCAAGAGCGTATAGCAGCCACCGCAGGGTACCTCGGCGGCGCGTTTGACGTTGAGTCTCTCG
Hua_5_CDS           CCTGTGTACAAAGCCTCTCTTCCTAAAAACCCCACCGTCCAAGAGCGTATAGCAGCCACCGCAGGGTACCTCGGCGGCGCGTTTGACGTTGAGTCTCTCG
                      P  V  Y  K  A  S  L  P  K  N  P  T  V  Q  E  R  I  A  A  T  A  G  Y  L  G  G  A  F  D  V  E  S  L
J7005_Genome        CCTGTGTACAAAGCCTCTCTTCCTAAAAACCCCACCGTCCAAGAGCGTATAGCAGCCACCGCAGGGTACCTCGGCGGCGCGTTTGACGTTGAGTCTCTCG
J7005_CDS           CCTGTGTACAAAGCCTCTCTTCCTAAAAACCCCACCGTCCAAGAGCGTATAGCAGCCACCGCAGGGTACCTCGGCGGCGCGTTTGACGTTGAGTCTCTCG
                      P  V  Y  K  A  S  L  P  K  N  P  T  V  Q  E  R  I  A  A  T  A  G  Y  L  G  G  A  F  D  V  E  S  L
AtAHK4              CCTGTCTACAAGTCTTCTCTTCCTGAAAATCCGACTGTCGAAGAGCGTATTGCAGCCACTGCAGGGTACCTTGGTGGTGCGTTTGATGTGGAGTCTCTAG
                      P  V  Y  K  S  S  L  P  E  N  P  T  V  E  E  R  I  A  A  T  A  G  Y  L  G  G  A  F  D  V  E  S  L
```

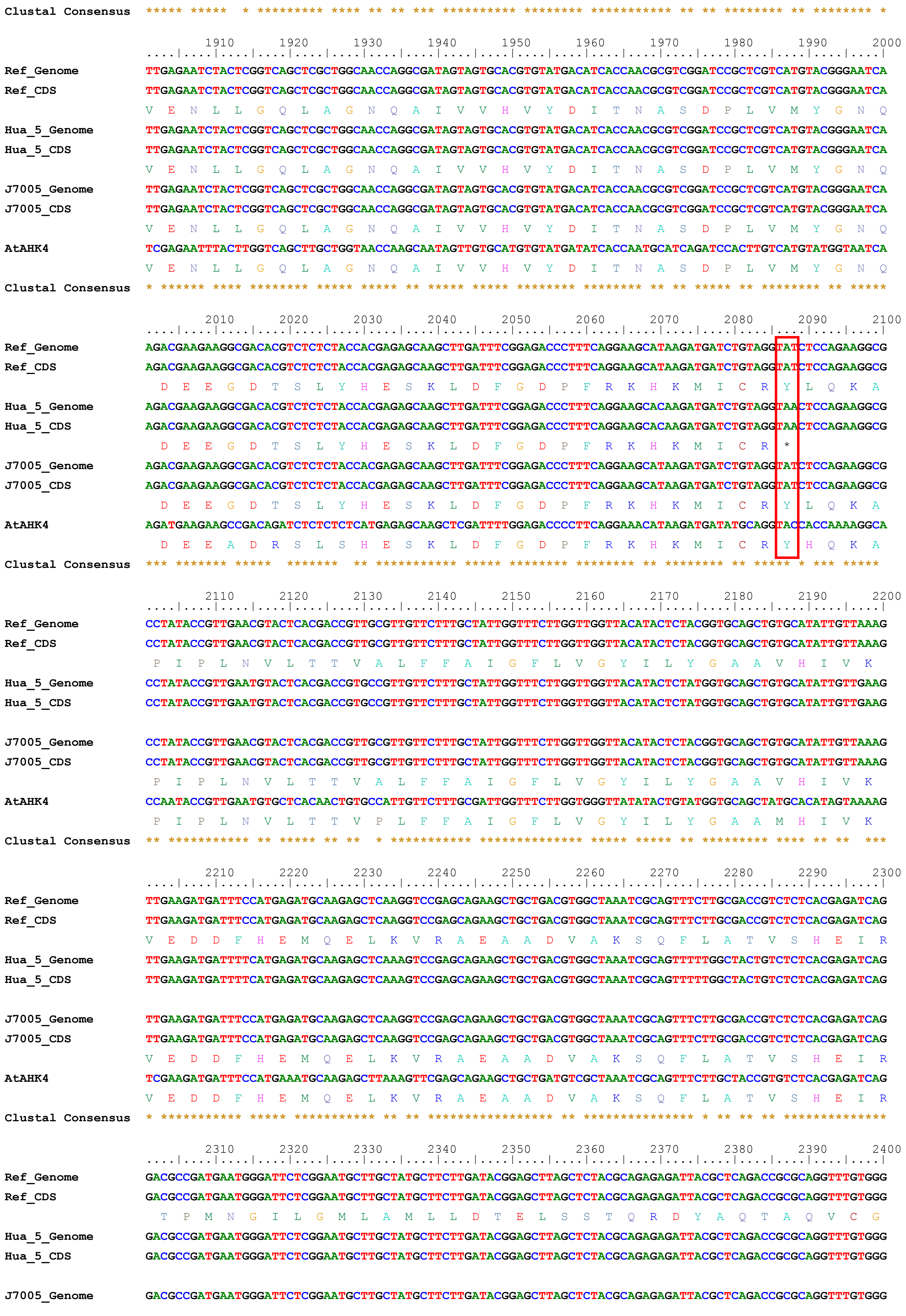

```
J7005_CDS           GACGCCGATGAATGGGATTCTCGGAATGCTTGCTATGCTTCTTGATACGGAGCTTAGCTCTACGCAGAGAGATTACGCTCAGACCGCGCAGGTTTGTGGG
                      T  P  M  N  G  I  L  G  M  L  A  M  L  L  D  T  E  L  S  S  T  Q  R  D  Y  A  Q  T  A  Q  V  C  G
AtAHK4              GACACCAATGAATGGCATTCTCGGAATGCTTGCTATGCTCCTAGATACAGAACTAAGCTCGACACAGAGAGATTACGCTCAAACCGCTCAAGTATGTGGT
                      T  P  M  N  G  I  L  G  M  L  A  M  L  L  D  T  E  L  S  S  T  Q  R  D  Y  A  Q  T  A  Q  V  C  G
Clustal Consensus   *** ** ******** *********************** ** ***** ** ** ***** ** ***************** ***** ** ** *****

                          2410      2420      2430      2440      2450      2460      2470      2480      2490      2500
                    ....|....|....|....|....|....|....|....|....|....|....|....|....|....|....|....|....|....|....|....|
Ref_Genome          AAAGCTTTGATTGCGTTGATAAACGAGGTCCTTGACCGTGCCAAGATCGAAGCTGGCAAGCTGGAGTTGGAGTCTGTGCCTTTTGATATCCGTTCGATAC
Ref_CDS             AAAGCTTTGATTGCGTTGATAAACGAGGTCCTTGACCGTGCCAAGATCGAAGCTGGCAAGCTGGAGTTGGAGTCTGTGCCTTTTGATATCCGTTCGATAC
                     K  A  L  I  A  L  I  N  E  V  L  D  R  A  K  I  E  A  G  K  L  E  L  E  S  V  P  F  D  I  R  S  I
Hua_5_Genome        AAAGCTTTGATTGCGTTGATAAACGAGGTTCTTGACCGTGCCAAGATCGAAGCTGGCAAGCTGGAGTTGGAGTCTGTGCCTTTTGATATCCGTTCGATAC
Hua_5_CDS           AAAGCTTTGATTGCGTTGATAAACGAGGTTCTTGACCGTGCCAAGATCGAAGCTGGCAAGCTGGAGTTGGAGTCTGTGCCTTTTGATATCCGTTCGATAC

J7005_Genome        AAAGCTTTGATTGCGTTGATAAACGAGGTCCTTGACCGTGCCAAGATCGAAGCTGGCAAGCTGGAGTTGGAGTCTGTGCCTTTTGATATCCGTTCGATAC
J7005_CDS           AAAGCTTTGATTGCGTTGATAAACGAGGTCCTTGACCGTGCCAAGATCGAAGCTGGCAAGCTGGAGTTGGAGTCTGTGCCTTTTGATATCCGTTCGATAC
                     K  A  L  I  A  L  I  N  E  V  L  D  R  A  K  I  E  A  G  K  L  E  L  E  S  V  P  F  D  I  R  S  I
AtAHK4              AAAGCTTTGATTGCATTGATAAATGAGGTTCTTGATCGCGCCAAGATTGAAGCTGGAAAGCTGGAGTTGGAATCAGTACCATTTGATATCCGTTCAATAT
                     K  A  L  I  A  L  I  N  E  V  L  D  R  A  K  I  E  A  G  K  L  E  L  E  S  V  P  F  D  I  R  S  I
Clustal Consensus   ************** ******** ***** ***** ** ******** ******** ************** ** ** ** ************** ***

                          2510      2520      2530      2540      2550      2560      2570      2580      2590      2600
                    ....|....|....|....|....|....|....|....|....|....|....|....|....|....|....|....|....|....|....|....|
Ref_Genome          TGGATGATGTTCTTTCTCTCTTCTCTGAGGAGTCAAGGAACAAAGGCATTGAGGTAAAAGAGTAGTGTATAAAGAAAATTACTTTTTTTTTGTAGTTTGC
Ref_CDS             TGGATGATGTTCTTTCTCTCTTCTCTGAGGAGTCAAGGAACAAAGGCATTGA------------------------------------------------
                    L  D  D  V  L  S  L  F  S  E  E  S  R  N  K  G  I  E
Hua_5_Genome        TGGATGATGTTCTTTCTCTCTTCTCTGAGGAGTCAAGGAACAAAGGCATTGAGGTAAAAGAGTAGTGTATAAGGAAAAGTCCTTT-TTTTTGTAGTTTGC
Hua_5_CDS           TGGATGATGTTCTTTCTCTCTTCTCTGAGGAGTCAAGGAACAAAGGCATTGA------------------------------------------------

J7005_Genome        TGGATGATGTTCTTTCTCTCTTCTCTGAGGAGTCAAGGAACAAAGGCATTGAGGTAAAAGAGTAGTGTATAAAGAAAATTACTTTTTTTTTGTAGTTTGC
J7005_CDS           TGGATGATGTTCTTTCTCTCTTCTCTGAGGAGTCAAGGAACAAAGGCATTGA------------------------------------------------
                    L  D  D  V  L  S  L  F  S  E  E  S  R  N  K  G  I  E
AtAHK4              TGGATGATGTCCTTTCTCTATTCTCTGAGGAGTCAAGGAACAAAAGCATTGA------------------------------------------------
                    L  D  D  V  L  S  L  F  S  E  E  S  R  N  K  S  I  E
Clustal Consensus   ********** ******** ************************ *******

                          2610      2620      2630      2640      2650      2660      2670      2680      2690      2700
                    ....|....|....|....|....|....|....|....|....|....|....|....|....|....|....|....|....|....|....|....|
Ref_Genome          TGCTACTTACTAACTTCTTGATTTTTTTGAATTTTTTGGCAGCTTGCGGTTTTCGTTTCAGACAAGGTACCTGAGATAGTCAAAGGAGATTCAGGGAGAT
Ref_CDS             -----------------------------------------GCTTGCGGTTTTCGTTTCAGACAAGGTACCTGAGATAGTCAAAGGAGATTCAGGGAGAT
                                                               L  A  V  F  V  S  D  K  V  P  E  I  V  K  G  D  S  G  R
Hua_5_Genome        TGCTACTTACTAACTTCTTGATTTTTTTGAATTTTTTGGCAGCTTGCGGTTTTCGTTTCAGACAAGGTACCTGAGATAGTCAAAGGAGATTCAGGGAGAT
Hua_5_CDS           -----------------------------------------GCTTGCGGTTTTCGTTTCAGACAAGGTACCTGAGATAGTCAAAGGAGATTCAGGGAGAT

J7005_Genome        TGCTACTTACTAACTTCTTGATTTTTTTGAATTTTTTGGCAGCTTGCGGTTTTCGTTTCAGACAAGGTACCTGAGATAGTCAAAGGAGATTCAGGGAGAT
J7005_CDS           -----------------------------------------GCTTGCGGTTTTCGTTTCAGACAAGGTACCTGAGATAGTCAAAGGAGATTCAGGGAGAT
                                                               L  A  V  F  V  S  D  K  V  P  E  I  V  K  G  D  S  G  R
AtAHK4              -----------------------------------------GCTCGCGGTTTTCGTTTCAGACAAAGTACCAGAGATAGTCAAAGGAGATTCAGGGAGAT
                                                               L  A  V  F  V  S  D  K  V  P  E  I  V  K  G  D  S  G  R
Clustal Consensus                                            *** ******************** ***** ****************************

                          2710      2720      2730      2740      2750      2760      2770      2780      2790      2800
                    ....|....|....|....|....|....|....|....|....|....|....|....|....|....|....|....|....|....|....|....|
Ref_Genome          TCAGACAGATCATCATAAACCTCGTCGGAAACTCTGTTAAAGTTAGTTCCTGTCCTCCTATATTCTTGTCTAGGCCTGCGGGTTCAGGTTGTTCAGTTTT
Ref_CDS             TCAGACAGATCATCATAAACCTCGTCGGAAACTCTGTTAAA-----------------------------------------------------------
                    F  R  Q  I  I  I  N  L  V  G  N  S  V  K
Hua_5_Genome        TCAGACAGATCATCATAAACCTCGTCGGAAACTCTGTTAAAGTTAGTTCCTTTCCTCCTATATTCTTGTCTAGGCCTGCGGGTTCAGGTTGTTCAGTTTT
Hua_5_CDS           TCAGACAGATCATCATAAACCTCGTCGGAAACTCTGTTAAA-----------------------------------------------------------

J7005_Genome        TCAGACAGATCATCATAAACCTCGTCGGAAACTCTGTTAAAGTTAGTTCCTGTCCTCCTATATTCTTGTCTAGGCCTGCGGGTTCAGGTTGTTCAGTTTT
J7005_CDS           TCAGACAGATCATCATAAACCTCGTCGGAAACTCTGTTAAA-----------------------------------------------------------
                    F  R  Q  I  I  I  N  L  V  G  N  S  V  K
AtAHK4              TTAGACAGATAATCATAAACCTTGTTGGAAATTCGGTTAAA-----------------------------------------------------------
                    F  R  Q  I  I  I  N  L  V  G  N  S  V  K
Clustal Consensus   * ******** *********** ** ***** ** ******

                          2810      2820      2830      2840      2850      2860      2870      2880      2890      2900
                    ....|....|....|....|....|....|....|....|....|....|....|....|....|....|....|....|....|....|....|....|
Ref_Genome          CGTTTAGTTTGGTTCAATACAAATCTTGAAGAATTAATCCGAAATATGTTCAGTTCGGTATTCGGTTGGTTGGGTTTTTGAAGATCCTACCGATGTTTTT
Ref_CDS             ----------------------------------------------------------------------------------------------------
```

```
Hua_5_Genome        CGTTTAGTTTGGTTCAATACAAATCTTGAAGAATTAACCCGAAATAAGTTCAGTTCGGTATTCGGTTGGTTGGGTTTTTGAAGATCCTACCGATGTTTTT
Hua_5_CDS           ----------------------------------------------------------------------------------------------------

J7005_Genome        CGTTTAGTTTGGTTCAATACAAATCTTGAAGAATTAATCCGAAATATGTTCAGTTCGGTATTCGGTTGGTTGGGTTTTTGAAGATCCTACCGATGTTTTT
J7005_CDS           ----------------------------------------------------------------------------------------------------

AtAHK4              ----------------------------------------------------------------------------------------------------

Clustal Consensus

                          2910      2920      2930      2940      2950      2960      2970      2980      2990      3000
                    ....|....|....|....|....|....|....|....|....|....|....|....|....|....|....|....|....|....|....|....|
Ref_Genome          GGCTTTGGTTATATTTTGGTTTAATTTTGTTAAAATTTAAAATAAGTTCTGTTAACTTCAGTTAGTTCGGTTTGAAATTTTGTTAGTTCAGTACAGATTT
Ref_CDS             ----------------------------------------------------------------------------------------------------

Hua_5_Genome        GGCTTTGGTTATATTTTGGTTTAATTTTGTTAAAATTTAAAATAAGTTTCGTTAACTTCAGTTAGTTCGGTTTGAAATTTTGTTAGTTCAGTACAGATTT
Hua_5_CDS           ----------------------------------------------------------------------------------------------------

J7005_Genome        GGCTTTGGTTATATTTTGGTTTAATTTTGTTAAAATTTAAAATAAGTTCTGTTAACTTCAGTTAGTTCGGTTTGAAATTTTGTTAGTTCAGTACAGATTT
J7005_CDS           ----------------------------------------------------------------------------------------------------

AtAHK4              ----------------------------------------------------------------------------------------------------

Clustal Consensus

                          3010      3020      3030      3040      3050      3060      3070      3080      3090      3100
                    ....|....|....|....|....|....|....|....|....|....|....|....|....|....|....|....|....|....|....|....|
Ref_Genome          TTGGTATGGTTTGGCTGTTATTTTTCAATTCTTTTAAAGAAAATTAAAGTAACCGACTGCCGAACCAAAAACCAAACTTTTTAAATAACCTACCAAATCG
Ref_CDS             ----------------------------------------------------------------------------------------------------

Hua_5_Genome        TTGGTATGGTTTGGCTGTTATTTTTCAATTCTTTTAAAGAAAATTAAAGTAACCGACTGCCGAACCAAAAACCAAACTTTTTAAATAACCTACCAAATCG
Hua_5_CDS           ----------------------------------------------------------------------------------------------------

J7005_Genome        TTGGTATGGTTTGGCTGTTATTTTTCAATTCTTTTAAAGAAAATTAAAGTAACCGACTGCCGAACCAAAAACCAAACTTTTTAAATAACCTACCAAATCG
J7005_CDS           ----------------------------------------------------------------------------------------------------

AtAHK4              ----------------------------------------------------------------------------------------------------

Clustal Consensus

                          3110      3120      3130      3140      3150      3160      3170      3180      3190      3200
                    ....|....|....|....|....|....|....|....|....|....|....|....|....|....|....|....|....|....|....|....|
Ref_Genome          AACCGAACTCCTAACCAAAATTTTGGTTCAGTCTAATTTAAAATCCCACCCTTATTCTTGTCCCCCTGTGGAAGTAGATTGTTGTTTGATCAAGATGTTG
Ref_CDS             ----------------------------------------------------------------------------------------------------

Hua_5_Genome        AACCGAACTCCTAACCAAAATTTTGGTTCAGTCTAATTTAAAATCCCTGCCCTATTGTTGTCCCCCTGTGGAAGTAGATTGTTGTTTGAACAAGATGATG
Hua_5_CDS           ----------------------------------------------------------------------------------------------------

J7005_Genome        AACCGAACTCCTAACCAAAATTTTGGTTCAGTCTAATTTAAAATCCCACCCTTATTCTTGTCCCCCTGTGGAAGTAGATTGTTGTTTGATCAAGATGTTG
J7005_CDS           ----------------------------------------------------------------------------------------------------

AtAHK4              ----------------------------------------------------------------------------------------------------

Clustal Consensus

                          3210      3220      3230      3240      3250      3260      3270      3280      3290      3300
                    ....|....|....|....|....|....|....|....|....|....|....|....|....|....|....|....|....|....|....|....|
Ref_Genome          TCTTTTTTCTTTTGACACAGTTCACAGAGAAAGGACATATCTTTGTCAAAGTCCATCTCGCGGAACAATCAAAAGACGGAGCTGAATCCAAACCCGCATT
Ref_CDS             --------------------TTCACAGAGAAAGGACATATCTTTGTCAAAGTCCATCTCGCGGAACAATCAAAAGACGGAGCTGAATCCAAACCCGCATT
                                         F  T  E  K  G  H  I  F  V  K  V  H  L  A  E  Q  S  K  D  G  A  E  S  K  P  A  L
Hua_5_Genome        TCTTTTTTTTTTTGACACAGTTCACAGAGAAAGGACATATCTTTGTCAAAGTCCATCTCGCGGAACAATCAAAAGACGGAGCTGAATCCAAACCCGCATT
Hua_5_CDS           --------------------TTCACAGAGAAAGGACATATCTTTGTCAAAGTCCATCTCGCGGAACAATCAAAAGACGGAGCTGAATCCAAACCCGCATT

J7005_Genome        TCTTTTTTCTTTTGACACAGTTCACAGAGAAAGGACATATCTTTGTCAAAGTCCATCTCGCGGAACAATCAAAAGACGGAGCTGAATCCAAACCCGCATT
J7005_CDS           --------------------TTCACAGAGAAAGGACATATCTTTGTCAAAGTCCATCTCGCGGAACAATCAAAAGACGGAGCTGAATCCAAACCCGCATT
                                         F  T  E  K  G  H  I  F  V  K  V  H  L  A  E  Q  S  K  D  G  A  E  S  K  P  A  L
AtAHK4              --------------------TTCACAGAGAAAGGACATATCTTTGTTAAAGTCCATCTTGCGGAACAATCAAAAGATGAATCTGAACCGAAAAATGCATT
                                         F  T  E  K  G  H  I  F  V  K  V  H  L  A  E  Q  S  K  D  E  S  E  P  K  N  A  L
Clustal Consensus                       ************************** *********** ***************** * * ***** * ***   *****
```

```
                          3310      3320      3330      3340      3350      3360      3370      3380      3390      3400
                    ....|....|....|....|....|....|....|....|....|....|....|....|....|....|....|....|....|....|....|....|
Ref_Genome          GAACGGAGGAGTAGCCTCTGAAGACATAACCGCCGCTTCCAAACCGTCGAGTTACAACACACTGAGCGGCTACGAAGCTGCTGACGGTCGAAACAGCTGG
Ref_CDS             GAACGGAGGAGTAGCCTCTGAAGACATAACCGCCGCTTCCAAACCGTCGAGTTACAACACACTGAGCGGCTACGAAGCTGCTGACGGTCGAAACAGCTGG
                      N  G  G  V  A  S  E  D  I  T  A  A  S  K  P  S  S  Y  N  T  L  S  G  Y  E  A  A  D  G  R  N  S  W
Hua_5_Genome        GAACGGAGGAGTAGCCTCTGAAGACATAACCGCCGCTTCCAACCCGTCGAGTTACAACACACTGAGCGGTTACGAAGCTGCTGACGGTCGAAACAGCTGG
Hua_5_CDS           GAACGGAGGAGTAGCCTCTGAAGACATAACCGCCGCTTCCAACCCGTCGAGTTACAACACACTGAGCGGTTACGAAGCTGCTGACGGTCGAAACAGCTGG

J7005_Genome        GAACGGAGGAGTAGCCTCTGAAGACATAACCGCCGCTTCCAAACCGTCGAGTTACAACACACTGAGCGGCTACGAAGCTGCTGACGGTCGAAACAGCTGG
J7005_CDS           GAACGGAGGAGTAGCCTCTGAAGACATAACCGCCGCTTCCAAACCGTCGAGTTACAACACACTGAGCGGCTACGAAGCTGCTGACGGTCGAAACAGCTGG
                      N  G  G  V  A  S  E  D  I  T  A  A  S  K  P  S  S  Y  N  T  L  S  G  Y  E  A  A  D  G  R  N  S  W
AtAHK4              GAATGGTGGAGT---GTCTGAAGAAATGATCGTTGTTTCCAAACAGTCAAGTTACAACACATTGAGCGGTTACGAAGCTGCTGATGGTCGGAATAGCTGG
                      N  G  G  V     S  E  E  M  I  V  V  S  K  Q  S  S  Y  N  T  L  S  G  Y  E  A  A  D  G  R  N  S  W
Clustal Consensus   *** ** *****    ******** ** * **  * ****** * *** ************ ******* ************** ***** ** ******

                          3410      3420      3430      3440      3450      3460      3470      3480      3490      3500
                    ....|....|....|....|....|....|....|....|....|....|....|....|....|....|....|....|....|....|....|....|
Ref_Genome          GACTCATTCAAACACTTGGTCTCGTCGGAGGAGCTGTTGACATCATCAGAGTTCGACGCTTCCAGTAACGTCAGGCTTATGGTTTCTATCGAAGACACAG
Ref_CDS             GACTCATTCAAACACTTGGTCTCGTCGGAGGAGCTGTTGACATCATCAGAGTTCGACGCTTCCAGTAACGTCAGGCTTATGGTTTCTATCGAAGACACAG
                     D  S  F  K  H  L  V  S  S  E  E  L  L  T  S  S  E  F  D  A  S  S  N  V  R  L  M  V  S  I  E  D  T
Hua_5_Genome        GACTCATTCAAACACTTAGTCTCGTCGGAGGAGCTGTTGACATCATCAGAGTTCGACGCTTCCAGTAACGTCAGGCTTATGGTTTCTATCGAAGACACAG
Hua_5_CDS           GACTCATTCAAACACTTAGTCTCGTCGGAGGAGCTGTTGACATCATCAGAGTTCGACGCTTCCAGTAACGTCAGGCTTATGGTTTCTATCGAAGACACAG

J7005_Genome        GACTCATTCAAACACTTGGTCTCGTCGGAGGAGCTGTTGACATCATCAGAGTTCGACGCTTCCAGTAACGTCAGGCTTATGGTTTCTATCGAAGACACAG
J7005_CDS           GACTCATTCAAACACTTGGTCTCGTCGGAGGAGCTGTTGACATCATCAGAGTTCGACGCTTCCAGTAACGTCAGGCTTATGGTTTCTATCGAAGACACAG
                     D  S  F  K  H  L  V  S  S  E  E  L  L  T  S  S  E  F  D  A  S  S  N  V  R  L  M  V  S  I  E  D  T
AtAHK4              GATTCATTCAAGCATTTGGTCTCTGAGGAGCA------GTCATTATCGGAGTTTGATATTTCTAGCAATGTTAGGCTTATGGTTTCAATCGAAGACACGG
                     D  S  F  K  H  L  V  S  E  E  Q        S  L  S  E  F  D  I  S  S  N  V  R  L  M  V  S  I  E  D  T
Clustal Consensus   ** ******** ** ** *****   **** *      * *** *** ***** **   *** ** ** ** ************** *********** *

                          3510      3520      3530      3540      3550      3560      3570      3580      3590      3600
                    ....|....|....|....|....|....|....|....|....|....|....|....|....|....|....|....|....|....|....|....|
Ref_Genome          GTATTGGGATCCCTTTAGCCGCGCAAGGACGTGTCTTCATGCCGTTTATGCAAGCGGATAGCTCCACTTCGAGAACTTACGGAGGTACTGGGATTGGTTT
Ref_CDS             GTATTGGGATCCCTTTAGCCGCGCAAGGACGTGTCTTCATGCCGTTTATGCAAGCGGATAGCTCCACTTCGAGAACTTACGGAGGTACTGGGATTGGTTT
                    G  I  G  I  P  L  A  A  Q  G  R  V  F  M  P  F  M  Q  A  D  S  S  T  S  R  T  Y  G  G  T  G  I  G  L
Hua_5_Genome        GCATTGGAATCCCTTTAACCGCGCAAGGACGTGTCTTCATGCCGTTTATGCAAGCGGATAGCTCCACTTCGAGAACTTACGGAGGTACTGGGATTGGTTT
Hua_5_CDS           GCATTGGAATCCCTTTAACCGCGCAAGGACGTGTCTTCATGCCGTTTATGCAAGCGGATAGCTCCACTTCGAGAACTTACGGAGGTACTGGGATTGGTTT

J7005_Genome        GTATTGGGATCCCTTTAGCCGCGCAAGGACGTGTCTTCATGCCGTTTATGCAAGCGGATAGCTCCACTTCGAGAACTTACGGAGGTACTGGGATTGGTTT
J7005_CDS           GTATTGGGATCCCTTTAGCCGCGCAAGGACGTGTCTTCATGCCGTTTATGCAAGCGGATAGCTCCACTTCGAGAACTTACGGAGGTACTGGGATTGGTTT
                    G  I  G  I  P  L  A  A  Q  G  R  V  F  M  P  F  M  Q  A  D  S  S  T  S  R  T  Y  G  G  T  G  I  G  L
AtAHK4              GTATTGGAATCCCTTTAGTTGCGCAAGGCCGTGTGTTTATGCCGTTTATGCAAGCAGATAGCTCGACTTCAAGAAACTATGGAGGTACTGGTATTGGTTT
                    G  I  G  I  P  L  V  A  Q  G  R  V  F  M  P  F  M  Q  A  D  S  S  T  S  R  N  Y  G  G  T  G  I  G  L
Clustal Consensus   * ***** *********   ******** ***** ** ***************** ******** ***** ****  ** *********** ********

                          3610      3620      3630      3640      3650      3660      3670      3680      3690      3700
                    ....|....|....|....|....|....|....|....|....|....|....|....|....|....|....|....|....|....|....|....|
Ref_Genome          GAGTATAAGCAAGTGTCTCGTCGAGCTTATGCGCGGTCAGATAAGTTTCGTGAGCAGGCCTCGCGTTGGTAGCACGTTCTGGTTCACCGCTGTGTTTGAG
Ref_CDS             GAGTATAAGCAAGTGTCTCGTCGAGCTTATGCGCGGTCAGATAAGTTTCGTGAGCAGGCCTCGCGTTGGTAGCACGTTCTGGTTCACCGCTGTGTTTGAG
                      S  I  S  K  C  L  V  E  L  M  R  G  Q  I  S  F  V  S  R  P  R  V  G  S  T  F  W  F  T  A  V  F  E
Hua_5_Genome        GAGTATAAGCAAGTGTCTCGTCGAGCTTATGCGCGGTCAGATAAGTTTCGTGAGCAGGCCTCGCGTTGGAAGCACGTTCTGGTTCACCGCTGTGTTTGAG
Hua_5_CDS           GAGTATAAGCAAGTGTCTCGTCGAGCTTATGCGCGGTCAGATAAGTTTCGTGAGCAGGCCTCGCGTTGGAAGCACGTTCTGGTTCACCGCTGTGTTTGAG

J7005_Genome        GAGTATAAGCAAGTGTCTCGTCGAGCTTATGCGCGGTCAGATAAGTTTCGTGAGCAGGCCTCGCGTTGGTAGCACGTTCTGGTTCACCGCTGTGTTTGAG
J7005_CDS           GAGTATAAGCAAGTGTCTCGTCGAGCTTATGCGCGGTCAGATAAGTTTCGTGAGCAGGCCTCGCGTTGGTAGCACGTTCTGGTTCACCGCTGTGTTTGAG
                      S  I  S  K  C  L  V  E  L  M  R  G  Q  I  S  F  V  S  R  P  R  V  G  S  T  F  W  F  T  A  V  F  E
AtAHK4              GAGTATAAGCAAGTGTCTTGTTGAACTTATGCGTGGTCAGATAAATTTCATAAGCCGGCCTCATATTGGAAGCACGTTCTGGTTCACGGCTGTTTTAGAG
                      S  I  S  K  C  L  V  E  L  M  R  G  Q  I  N  F  I  S  R  P  H  I  G  S  T  F  W  F  T  A  V  L  E
Clustal Consensus   ****************** ** ** ******** ********** **** * *** ******   **** ***************** ***** ** ***

                          3710      3720      3730      3740      3750      3760      3770      3780      3790      3800
                    ....|....|....|....|....|....|....|....|....|....|....|....|....|....|....|....|....|....|....|....|
Ref_Genome          AGGTGTGATAAATGCAGTC------------TGAAGAAGCCTACGGTTGAGAATCTGCCTTCTAGTTTTAGAGGGATGAGAGCTATTGTTGTTGATGCTA
Ref_CDS             AGGTGTGATAAATGCAGTC------------TGAAGAAGCCTACGGTTGAGAATCTGCCTTCTAGTTTTAGAGGGATGAGAGCTATTGTTGTTGATGCTA
                     R  C  D  K  C  S              L  K  K  P  T  V  E  N  L  P  S  S  F  R  G  M  R  A  I  V  V  D  A
Hua_5_Genome        AGGTGTGATAAATGTAGTC------------TGAAGAAGCCTACGGTTGAGAATTTGCCTTCTAGTTTTAGAGGGATGAGAGCTATTGTTGTTGATGCTA
Hua_5_CDS           AGGTGTGATAAATGTAGTC------------TGAAGAAGCCTACGGTTGAGAATTTGCCTTCTAGTTTTAGAGGGATGAGAGCTATTGTTGTTGATGCTA

J7005_Genome        AGGTGTGATAAATGCAGTC------------TGAAGAAGCCTACGGTTGAGAATCTGCCTTCTAGTTTTAGAGGGATGAGAGCTATTGTTGTTGATGCTA
J7005_CDS           AGGTGTGATAAATGCAGTC------------TGAAGAAGCCTACGGTTGAGAATCTGCCTTCTAGTTTTAGAGGGATGAGAGCTATTGTTGTTGATGCTA
                     R  C  D  K  C  S              L  K  K  P  T  V  E  N  L  P  S  S  F  R  G  M  R  A  I  V  V  D  A
```

```
AtAHK4             AAATGCGATAAATGCAGTGCGATTAACCATATGAAGAAACCTAATGTGGAACACTTGCCTTCTACTTTTAAAGGAATGAAAGCTATAGTTGTTGATGCTA
                    K  C  D  K  C  S  A  I  N  H  M  K  K  P  N  V  E  H  L  P  S  T  F  K  G  M  K  A  I  V  V  D  A
Clustal Consensus  *  ** ******** ***              ******* ****  ** **  *  ********* ***** *** **** ****** *************

                           3810      3820      3830      3840      3850      3860      3870      3880      3890      3900
                   ....|....|....|....|....|....|....|....|....|....|....|....|....|....|....|....|....|....|....|....|
Ref_Genome         AGCCTGTTCGAGCTGCGGTGACTAGGTATCATATGAAAAGACTTGGGATCAGTGTAGATGTCATGACAAGTCTCAGAACCGCTGTTT------CTACTGC
Ref_CDS            AGCCTGTTCGAGCTGCGGTGACTAGGTATCATATGAAAAGACTTGGGATCAGTGTAGATGTCATGACAAGTCTCAGAACCGCTGTTT------CTACTGC
                   K  P  V  R  A  A  V  T  R  Y  H  M  K  R  L  G  I  S  V  D  V  M  T  S  L  R  T  A  V        S  T  A
Hua_5_Genome       AGCCTGTTCGAGCTGCGGTGACTAGGTACCATATGAAAAGACTTGGGATCAGTGTTGATGTCATGACAAGTCTCAGAACCGCTGTTT------CTACTGC
Hua_5_CDS          AGCCTGTTCGAGCTGCGGTGACTAGGTACCATATGAAAAGACTTGGGATCAGTGTTGATGTCATGACAAGTCTCAGAACCGCTGTTT------CTACTGC

J7005_Genome       AGCCTGTTCGAGCTGCGGTGACTAGGTATCATATGAAAAGACTTGGGATCAGTGTAGATGTCATGACAAGTCTCAGAACCGCTGTTT------CTACTGC
J7005_CDS          AGCCTGTTCGAGCTGCGGTGACTAGGTATCATATGAAAAGACTTGGGATCAGTGTAGATGTCATGACAAGTCTCAGAACCGCTGTTT------CTACTGC
                   K  P  V  R  A  A  V  T  R  Y  H  M  K  R  L  G  I  S  V  D  V  M  T  S  L  R  T  A  V        S  T  A
AtAHK4             AGCCTGTTAGAGCTGCTGTGACTAGATACCATATGAAAAGACTCGGAATCAATGTTGATGTCGTGACAAGTCTCAAAACCGCTGTTGTTGCAGCTGCTGC
                   K  P  V  R  A  A  V  T  R  Y  H  M  K  R  L  G  I  N  V  D  V  V  T  S  L  K  T  A  V  V  A  A  A  A
Clustal Consensus  ******** ******* ******** ** ************** ** **** *** ****** ************ **********       ** ****

                           3910      3920      3930      3940      3950      3960      3970      3980      3990      4000
                   ....|....|....|....|....|....|....|....|....|....|....|....|....|....|....|....|....|....|....|....|
Ref_Genome         GTCTGGAAGAAACGGTTCTCCTCTTCCTTCAGGGTTAGTAAAGATTCAATCTTTTATGTTTATATGTGTTGTTTCCATCTAAAGATTGAATCTTTTTTTT
Ref_CDS            GTCTGGAAGAAACGGTTCTCCTCTTCCTTCAG--------------------------------------------------------------------
                     S  G  R  N  G  S  P  L  P  S
Hua_5_Genome       GTCTGGAAGAAACGGTTCTCCTCTTCCTTCAGGGTTAGTAAAGATTCAATCTTTTATGTTTATATGTGTTGTTTCCATCTAAAGATTGAATCTTTTTTTT
Hua_5_CDS          GTCTGGAAGAAACGGTTCTCCTCTTCCTTCAG--------------------------------------------------------------------

J7005_Genome       GTCTGGAAGAAACGGTTCTCCTCTTCCTTCAGGGTTAGTAAAGATTCAATCTTTTATGTTTATATGTGTTGTTTCCATCTAAAGATTGAATCTTTTTTTT
J7005_CDS          GTCTGGAAGAAACGGTTCTCCTCTTCCTTCAG--------------------------------------------------------------------
                     S  G  R  N  G  S  P  L  P  S
AtAHK4             GTTTGAAAGAAACGGTTCTCCTCTCCCAA-----------------------------------------------------------------------
                     F  E  R  N  G  S  P  L  P
Clustal Consensus  ** ** ****************** **

                           4010      4020      4030      4040      4050      4060      4070      4080      4090      4100
                   ....|....|....|....|....|....|....|....|....|....|....|....|....|....|....|....|....|....|....|....|
Ref_Genome         GCAAGAACAACGAAACTGGATATGATCTTGGTGGAGAAAGATTCATGGATATCAACCGAAGATATAGACGCAGAGATACGTCAAATGAACTCAAGAACCA
Ref_CDS            ----GAACAACGAAACTGGATATGATCTTGGTGGAGAAAGATTCATGGATATCAACCGAAGATATAGACGCAGAGATACGTCAAATGAACTCAAGAACCA
                       G  T  T  K  L  D  M  I  L  V  E  K  D  S  W  I  S  T  E  D  I  D  A  E  I  R  Q  M  N  S  R  T
Hua_5_Genome       TGCAGAACAACGAAACTGGATATGATCTTGGTGGAGAAAGATTCATGGATATCAACCGAAGATATAGACGCGGAGATACGTCAGATGAACTCAAGAACCA
Hua_5_CDS          ----GAACAACGAAACTGGATATGATCTTGGTGGAGAAAGATTCATGGATATCAACCGAAGATATAGACGCGGAGATACGTCAGATGAACTCAAGAACCA

J7005_Genome       GCAAGAACAACGAAACTGGATATGATCTTGGTGGAGAAAGATTCATGGATATCAACCGAAGATATAGACGCAGAGATACGTCAAATGAACTCAAGAACCA
J7005_CDS          ----GAACAACGAAACTGGATATGATCTTGGTGGAGAAAGATTCATGGATATCAACCGAAGATATAGACGCAGAGATACGTCAAATGAACTCAAGAACCA
                       G  T  T  K  L  D  M  I  L  V  E  K  D  S  W  I  S  T  E  D  I  D  A  E  I  R  Q  M  N  S  R  T
AtAHK4             ----CAAAACCGCAACTTGATATGATCTTAGTAGAGAAAGATTCATGGATTTCAACTGAAGATAATGACTCAGAGATTCGTTTATTGAATTCAAGAACCA
                       T  K  P  Q  L  D  M  I  L  V  E  K  D  S  W  I  S  T  E  D  N  D  S  E  I  R  L  L  N  S  R  T
Clustal Consensus       ** * ** **** *********** ** ***************** ***** *******  *** * ***** ***     **** **********

                           4110      4120      4130      4140      4150      4160      4170      4180      4190      4200
                   ....|....|....|....|....|....|....|....|....|....|....|....|....|....|....|....|....|....|....|....|
Ref_Genome         ACGGAAACGTGCATCACAAGACACCGAAACTCGCTCTCTTCGCAACGAACATCACGAACTCAGAATTCGACAGAGCTAAATCAGCAGGGTTTGCGGATAC
Ref_CDS            ACGGAAACGTGCATCACAAGACACCGAAACTCGCTCTCTTCGCAACGAACATCACGAACTCAGAATTCGACAGAGCTAAATCAGCAGGGTTTGCGGATAC
                   N  G  N  V  H  H  K  T  P  K  L  A  L  F  A  T  N  I  T  N  S  E  F  D  R  A  K  S  A  G  F  A  D  T
Hua_5_Genome       ACGGAAACGTGCATCGCAAGACACCGAAACTCGCTCTGTTCGCAACGAACATTACGAATTCAGAATTCGACAGAGCTAAATCAGCAGGGTTTGCGGATAC
Hua_5_CDS          ACGGAAACGTGCATCGCAAGACACCGAAACTCGCTCTGTTCGCAACGAACATTACGAATTCAGAATTCGACAGAGCTAAATCAGCAGGGTTTGCGGATAC

J7005_Genome       ACGGAAACGTGCATCACAAGACACCGAAACTCGCTCTCTTCGCAACGAACATCACGAACTCAGAATTCGACAGAGCTAAATCAGCAGGGTTTGCGGATAC
J7005_CDS          ACGGAAACGTGCATCACAAGACACCGAAACTCGCTCTCTTCGCAACGAACATCACGAACTCAGAATTCGACAGAGCTAAATCAGCAGGGTTTGCGGATAC
                   N  G  N  V  H  H  K  T  P  K  L  A  L  F  A  T  N  I  T  N  S  E  F  D  R  A  K  S  A  G  F  A  D  T
AtAHK4             ACGGAAACGTTCATCACAAGTCTCCGAAACTAGCTCTATTCGCAACAAACATCACAAATTCGGAGTTCGACAGAGCTAAATCCGCAGGATTTGCAGATAC
                   N  G  N  V  H  H  K  S  P  K  L  A  L  F  A  T  N  I  T  N  S  E  F  D  R  A  K  S  A  G  F  A  D  T
Clustal Consensus  ********** **** **** * ******** ***** ******** ***** ** ** ** ** ***************** ***** ***** *****

                           4210      4220      4230      4240      4250      4260      4270      4280      4290      4300
                   ....|....|....|....|....|....|....|....|....|....|....|....|....|....|....|....|....|....|....|....|
Ref_Genome         GGTGATAATGAAGCCGTTGAGAGCAAGCATGATCGGCGCGTGTTTACAGCAAGTTCTCGAGCTGAGAAAGGCGAGACAGCAGCATCCTGAGGGATCATCA
Ref_CDS            GGTGATAATGAAGCCGTTGAGAGCAAGCATGATCGGCGCGTGTTTACAGCAAGTTCTCGAGCTGAGAAAGGCGAGACAGCAGCATCCTGAGGGATCATCA
                     V  I  M  K  P  L  R  A  S  M  I  G  A  C  L  Q  Q  V  L  E  L  R  K  A  R  Q  Q  H  P  E  G  S  S
Hua_5_Genome       GGTGATAATGAAGCCGTTGAGAGCAAGCATGATCGGCGCGTGTTTACAGCAAGTTCTCGAGCTGAGAAAGGCGAGACAGCAGCATCCTGAGGGATCATCA
Hua_5_CDS          GGTGATAATGAAGCCGTTGAGAGCAAGCATGATCGGCGCGTGTTTACAGCAAGTTCTCGAGCTGAGAAAGGCGAGACAGCAGCATCCTGAGGGATCATCA
```

```
J7005_Genome        GGTGATAATGAAGCCGTTGAGAGCAAGCATGATCGGCGCGTGTTTACAGCAAGTTCTCGAGCTGAGAAAGGCGAGACAGCAGCATCCTGAGGGATCATCA
J7005_CDS           GGTGATAATGAAGCCGTTGAGAGCAAGCATGATCGGCGCGTGTTTACAGCAAGTTCTCGAGCTGAGAAAGGCGAGACAGCAGCATCCTGAGGGATCATCA
                     V  I  M  K  P  L  R  A  S  M  I  G  A  C  L  Q  Q  V  L  E  L  R  K  A  R  Q  Q  H  P  E  G  S  S
AtAHK4              GGTAATAATGAAACCGTTAAGAGCAAGCATGATTGGGGCGTGTCTGCAACAAGTTCTCGAGCTGAGAAAAACAAGACAACAACATCCAGAAGGATCATCA
                     V  I  M  K  P  L  R  A  S  M  I  G  A  C  L  Q  Q  V  L  E  L  R  K  T  R  Q  Q  H  P  E  G  S  S
Clustal Consensus   *** ******** ***** ************** ** ****** * ** ********************  * ***** ** ***** ** *********

                             4310      4320      4330      4340      4350      4360      4370      4380      4390      4400
                    ....|....|....|....|....|....|....|....|....|....|....|....|....|....|....|....|....|....|....|....|
Ref_Genome          CCAGCAACGCTCAAGAGTTTGCTTACAGGGAAGAAGATTCTGGTGGTTGATGATAATATGGTGAACAGAAGAGTAGCTGCAGGAGCTCTGAAGAAGTTTG
Ref_CDS             CCAGCAACGCTCAAGAGTTTGCTTACAGGGAAGAAGATTCTGGTGGTTGATGATAATATGGTGAACAGAAGAGTAGCTGCAGGAGCTCTGAAGAAGTTTG
                     P  A  T  L  K  S  L  L  T  G  K  K  I  L  V  V  D  D  N  M  V  N  R  R  V  A  A  G  A  L  K  K  F
Hua_5_Genome        CCAGCAACGCTCAAGAGTTTGCTTACAGGGAAGAAGATTCTGGTGGTTGATGATAATATGGTGAACAGAAGAGTAGCTGCAGGAGCTCTGAAGAAGTTTG
Hua_5_CDS           CCAGCAACGCTCAAGAGTTTGCTTACAGGGAAGAAGATTCTGGTGGTTGATGATAATATGGTGAACAGAAGAGTAGCTGCAGGAGCTCTGAAGAAGTTTG

J7005_Genome        CCAGCAACGCTCAAGAGTTTGCTTACAGGGAAGAAGATTCTGGTGGTTGATGATAATATGGTGAACAGAAGAGTAGCTGCAGGAGCTCTGAAGAAGTTTG
J7005_CDS           CCAGCAACGCTCAAGAGTTTGCTTACAGGGAAGAAGATTCTGGTGGTTGATGATAATATGGTGAACAGAAGAGTAGCTGCAGGAGCTCTGAAGAAGTTTG
                     P  A  T  L  K  S  L  L  T  G  K  K  I  L  V  V  D  D  N  M  V  N  R  R  V  A  A  G  A  L  K  K  F
AtAHK4              CCCGCAACTCTCAAGAGCTTGCTTACAGGGAAGAAGATTCTTGTGGTTGATGATAATATAGTTAACAGGAGAGTAGCTGCAGGAGCTCTCAAGAAATTTG
                     P  A  T  L  K  S  L  L  T  G  K  K  I  L  V  V  D  D  N  I  V  N  R  R  V  A  A  G  A  L  K  K  F
Clustal Consensus   ** ***** ******** *********************** ***************** ** ***** ******************** ***** ****

                             4410      4420      4430      4440      4450      4460      4470      4480      4490      4500
                    ....|....|....|....|....|....|....|....|....|....|....|....|....|....|....|....|....|....|....|....|
Ref_Genome          GAGCAGAGGTGGTGTGTGCAGAGAGTGGTCAAGTTGCTTTGGGTTTGCTTCAGATTCCACACAGTTTCGATGCTTGCTTCATGGATATTCAAATGCCTCA
Ref_CDS             GAGCAGAGGTGGTGTGTGCAGAGAGTGGTCAAGTTGCTTTGGGTTTGCTTCAGATTCCACACAGTTTCGATGCTTGCTTCATGGATATTCAAATGCCTCA
                    G  A  E  V  V  C  A  E  S  G  Q  V  A  L  G  L  L  Q  I  P  H  S  F  D  A  C  F  M  D  I  Q  M  P  Q
Hua_5_Genome        GAGCAGAGGTGGTGTGTGCAGAGAGTGGTCAAGTTGCTTTGGGTTTGCTTCAGATTCCACACAGTTTCGATGCTTGCTTCATGGATATTCAAATGCCTCA
Hua_5_CDS           GAGCAGAGGTGGTGTGTGCAGAGAGTGGTCAAGTTGCTTTGGGTTTGCTTCAGATTCCACACAGTTTCGATGCTTGCTTCATGGATATTCAAATGCCTCA

J7005_Genome        GAGCAGAGGTGGTGTGTGCAGAGAGTGGTCAAGTTGCTTTGGGTTTGCTTCAGATTCCACACAGTTTCGATGCTTGCTTCATGGATATTCAAATGCCTCA
J7005_CDS           GAGCAGAGGTGGTGTGTGCAGAGAGTGGTCAAGTTGCTTTGGGTTTGCTTCAGATTCCACACAGTTTCGATGCTTGCTTCATGGATATTCAAATGCCTCA
                    G  A  E  V  V  C  A  E  S  G  Q  V  A  L  G  L  L  Q  I  P  H  S  F  D  A  C  F  M  D  I  Q  M  P  Q
AtAHK4              GAGCAGAAGTGGTTTGTGCAGAGAGTGGTCAAGTTGCTTTGGGTTTGCTTCAGATTCCACACACTTTCGATGCTTGCTTCATGGATATTCAAATGCCACA
                    G  A  E  V  V  C  A  E  S  G  Q  V  A  L  G  L  L  Q  I  P  H  T  F  D  A  C  F  M  D  I  Q  M  P  Q
Clustal Consensus   ******* ***** ************************************************* ********************************* **

                             4510      4520      4530      4540      4550      4560      4570      4580      4590      4600
                    ....|....|....|....|....|....|....|....|....|....|....|....|....|....|....|....|....|....|....|....|
Ref_Genome          GATGGACGGGTAAGCCTTTAAACTTGGCCTGCTGTTCGGTAGCTCGATTTTCGGTTAGTTCGGCTCAACATAAATCTTACCGAATTA-ATCCAAAATAAA
Ref_CDS             GATGGACGGG-------------------------------------------------------------------------------------------
                     M  D  G
Hua_5_Genome        GATGGACGGGTAAGCCTTTAAACTTGGCCTGCTGTTCGGTAGCTCGATTTTCGGTTAGTTCGGCTCAACATAAATCTTACCGAATTAAATCCAAAATAAA
Hua_5_CDS           GATGGACGGG-------------------------------------------------------------------------------------------

J7005_Genome        GATGGACGGGTAAGCCTTTAAACTTGGCCTGCTGTTCGGTAGCTCGATTTTCGGTTAGTTCGGCTCAACATAAATCTTACCGAATTA-ATCCAAAATAAA
J7005_CDS           GATGGACGGG-------------------------------------------------------------------------------------------
                     M  D  G
AtAHK4              GATGGACGGA-------------------------------------------------------------------------------------------
                     M  D  G
Clustal Consensus   *********

                             4610      4620      4630      4640      4650      4660      4670      4680      4690      4700
                    ....|....|....|....|....|....|....|....|....|....|....|....|....|....|....|....|....|....|....|....|
Ref_Genome          GTTTGGTTCGGTATTCGGTTAGTTTGGTTTTTGAAAATCGTACATAAGTTTTTGATTTGGGTTTATATTTTGATTAATTTTGTTAAAATTTCGGATGAGT
Ref_CDS             ----------------------------------------------------------------------------------------------------

Hua_5_Genome        GTTTGGTTCGGTATTCGGTTAGTTTGGTTTTTGAAAATCGTACATAAGTTTTTGATTTGGGTTTATATTTTGATTAATTTTGTTAAAATTTCGGATGAGT
Hua_5_CDS           ----------------------------------------------------------------------------------------------------

J7005_Genome        GTTTGGTTCGGTATTCGGTTAGTTTGGTTTTTGAAAATCGTACATAAGTTTTTGATTTGGGTTTATATTTTGATTAATTTTGTTAAAATTTCGGATGAGT
J7005_CDS           ----------------------------------------------------------------------------------------------------

AtAHK4              ----------------------------------------------------------------------------------------------------

Clustal Consensus

                             4710      4720      4730      4740      4750      4760      4770      4780      4790      4800
                    ....|....|....|....|....|....|....|....|....|....|....|....|....|....|....|....|....|....|....|....|
Ref_Genome          TCGGTTTGTTCGATTTGGGTTTTTGATATTGTTTGGTTATTTTTTTTTAAAGAAAACCAAAGTAACCGATTGCCAAACCAAACCTTTTCAAACCTACAAA
```

```
Ref_CDS             ----------------------------------------------------------------------------------------------------

Hua_5_Genome        TCGGTTGGTTCGGTTTGGGTTTTTGATATTGTTTGGTGATTTTTTTTTAAAGAAAACCAAAGTAACCGATTGCCAAACCAAACCTTTTCAAACCTACAAA
Hua_5_CDS           ----------------------------------------------------------------------------------------------------

J7005_Genome        TCGGTTTGTTCGATTTGGGTTTTTGATATTGTTTGGTTATTTTTTTTTAAAGAAAACCAAAGTAACCGATTGCCAAACCAAACCTTTTCAAACCTACAAA
J7005_CDS           ----------------------------------------------------------------------------------------------------

AtAHK4              ----------------------------------------------------------------------------------------------------

Clustal Consensus

                             4810      4820      4830      4840      4850      4860      4870      4880      4890      4900
                    ....|....|....|....|....|....|....|....|....|....|....|....|....|....|....|....|....|....|....|....|
Ref_Genome          ATCGAACCAAACTCTCAACTTTTTTGGTTCGGTTCAAAATCCCAGCCCTACTTAAACTCATTAAGTTTGGTCTTGTACTACTTGGTTTACGTTCTGGTTT
Ref_CDS             ----------------------------------------------------------------------------------------------------

Hua_5_Genome        ATCGAACCAAACTCTCAACTTTTTTGGTTCGGTTCAAAATCCCAGCCCTACTTAAACTCATTAGGTTTGGTCTTGTACTACTTGGTTTACGTTCTGGTTT
Hua_5_CDS           ----------------------------------------------------------------------------------------------------

J7005_Genome        ATCGAACCAAACTCTCAACTTTTTTGGTTCGGTTCAAAATCCCAGCCCTACTTAAACTCATTAAGTTTGGTCTTGTACTACTTGGTTTACGTTCTGGTTT
J7005_CDS           ----------------------------------------------------------------------------------------------------

AtAHK4              ----------------------------------------------------------------------------------------------------

Clustal Consensus

                             4910      4920      4930      4940      4950      4960      4970      4980      4990      5000
                    ....|....|....|....|....|....|....|....|....|....|....|....|....|....|....|....|....|....|....|....|
Ref_Genome          TGTTCCTCTCAGGTTTGAAGCGACTCGTCAGATAAGGATGATGGAGAAGGAAGCTAAAGAGAAGACGAAGCTGGAATGGCATTTACCGATTCTAGCCATG
Ref_CDS             -------------TTTGAAGCGACTCGTCAGATAAGGATGATGGAGAAGGAAGCTAAAGAGAAGACGAAGCTGGAATGGCATTTACCGATTCTAGCCATG
                                  F  E  A  T  R  Q  I  R  M  M  E  K  E  A  K  E  K  T  K  L  E  W  H  L  P  I  L  A  M
Hua_5_Genome        TGTTCCTCTCAGGTTTGAAGCGACTCGTCAGATAAGGATGATGGAGAAGGAAGCTAAAGAGAAGACGAAGCTGGAATGGCATTTACCGATTCTAGCCATG
Hua_5_CDS           -------------TTTGAAGCGACTCGTCAGATAAGGATGATGGAGAAGGAAGCTAAAGAGAAGACGAAGCTGGAATGGCATTTACCGATTCTAGCCATG

J7005_Genome        TGTTCCTCTCAGGTTTGAAGCGACTCGTCAGATAAGGATGATGGAGAAGGAAGCTAAAGAGAAGACGAAGCTGGAATGGCATTTACCGATTCTAGCCATG
J7005_CDS           -------------TTTGAAGCGACTCGTCAGATAAGGATGATGGAGAAGGAAGCTAAAGAGAAGACGAAGCTGGAATGGCATTTACCGATTCTAGCCATG
                                  F  E  A  T  R  Q  I  R  M  M  E  K  E  A  K  E  K  T  K  L  E  W  H  L  P  I  L  A  M
AtAHK4              -------------TTTGAAGCAACTCGTCAGATAAGAATGATGGAGAAGGAAACTAAAGAGAAGACAAATCTCGAATGGCATTTACCGATTCTAGCGATG
                                  F  E  A  T  R  Q  I  R  M  M  E  K  E  T  K  E  K  T  N  L  E  W  H  L  P  I  L  A  M
Clustal Consensus                ******** ************** *************** ************* ** ** *********************** ***

                             5010      5020      5030      5040      5050      5060      5070      5080      5090      5100
                    ....|....|....|....|....|....|....|....|....|....|....|....|....|....|....|....|....|....|....|....|
Ref_Genome          ACAGCTGATGTGATCCACGCGACATACGAGGAGTGTCTGAAAAGTGGAATGGATGGTTATGTCTCTAAACCATTTGAAGAAGAGAATCTCTACAAGTCTG
Ref_CDS             ACAGCTGATGTGATCCACGCGACATACGAGGAGTGTCTGAAAAGTGGAATGGATGGTTATGTCTCTAAACCATTTGAAGAAGAGAATCTCTACAAGTCTG
                     T  A  D  V  I  H  A  T  Y  E  E  C  L  K  S  G  M  D  G  Y  V  S  K  P  F  E  E  E  N  L  Y  K  S
Hua_5_Genome        ACAGCTGATGTGATCCACGCGACATACGAGGAGTGTCTGAAAAGTGGAATGGATGGTTATGTCTCTAAACCATTTGAAGAAGAGAATCTCTACAAGTCTG
Hua_5_CDS           ACAGCTGATGTGATCCACGCGACATACGAGGAGTGTCTGAAAAGTGGAATGGATGGTTATGTCTCTAAACCATTTGAAGAAGAGAATCTCTACAAGTCTG

J7005_Genome        ACAGCTGATGTGATCCACGCGACATACGAGGAGTGTCTGAAAAGTGGAATGGATGGTTATGTCTCTAAACCATTTGAAGAAGAGAATCTCTACAAGTCTG
J7005_CDS           ACAGCTGATGTGATCCACGCGACATACGAGGAGTGTCTGAAAAGTGGAATGGATGGTTATGTCTCTAAACCATTTGAAGAAGAGAATCTCTACAAGTCTG
                     T  A  D  V  I  H  A  T  Y  E  E  C  L  K  S  G  M  D  G  Y  V  S  K  P  F  E  E  E  N  L  Y  K  S
AtAHK4              ACTGCGGATGTGATACACGCGACCTACGAGGAATGTCTGAAAAGTGGGATGGATGGTTACGTCTCCAAACCTTTTGAAGAAGAGAATCTCTATAAATCCG
                     T  A  D  V  I  H  A  T  Y  E  E  C  L  K  S  G  M  D  G  Y  V  S  K  P  F  E  E  E  N  L  Y  K  S
Clustal Consensus   ** ** ******** ******** ******** ************** *********** ***** ***** ******************** ** ** *

                             5110      5120      5130      5140      5150
                    ....|....|....|....|....|....|....|....|....|....|....|.
Ref_Genome          TTGCCAAATCATTCAAAGCTAACCCAATCTCAGATTCGTCATGTAGCCAAAGTTGA
Ref_CDS             TTGCCAAATCATTCAAAGCTAACCCAATCTCAGATTCGTCATGTAGCCAAAGTTGA
                    V  A  K  S  F  K  A  N  P  I  S  D  S  S  C  S  Q  S  *
Hua_5_Genome        TTGCCAAATCATTCAAAGCTAACCCAATCTCTGATTCGTCATGTAGCCAAAGTTGA
Hua_5_CDS           TTGCCAAATCATTCAAAGCTAACCCAATCTCTGATTCGTCATGTAGCCAAAGTTGA

J7005_Genome        TTGCCAAATCATTCAAAGCTAACCCAATCTCAGATTCGTCATGTAGCCAAAGTTGA
J7005_CDS           TTGCCAAATCATTCAAAGCTAACCCAATCTCAGATTCGTCATGTAGCCAAAGTTGA
                    V  A  K  S  F  K  A  N  P  I  S  D  S  S  C  S  Q  S  *
AtAHK4              TTGCCAAATCATTCAAACCTAATCCTATCTCACCTTCGTCGTAA------------
                    V  A  K  S  F  K  P  N  P  I  S  P  S  S  *
```

Clustal Consensus ***************** **** ** *****   ****** *

Supplementary Figure S7. Alignment of the nucleotide sequences of *AHK4* genes (genomic and CDS sequences of BnaC07g21260D) and predicated AHK4 amino acid sequences from *Brassica napus* reference genome (Darmor-*bzh*), the two parents (Hua_5 and J7005), and Arabidopsis (AT2G01830.1). The red box refers to the premature termination of amino acid translation caused by point mutation in Hua_5.
